# A computational model of reward learning and habits on social media

**DOI:** 10.1038/s41467-026-73547-6

**Published:** 2026-06-04

**Authors:** Georgia Turner, Lukas J. Gunschera, Shashanka Subrahmanya, Aadesh Salecha, Johannes C. Eichstaedt, Stefano Palminteri, Amy Orben

**Affiliations:** 1 MRC Cognition and Brain Sciences Unit, University of Cambridge, Cambridge, UK; 2 Department of Psychology, Stanford University, Stanford, CA USA; 3 Institute for Human-Centered AI, Stanford University, Stanford, CA USA; 4 Decision Sciences, INSEAD, Fontainebleau, France; 5 Département d’Études Cognitives, École Normale Supérieure, Paris, France; 6 Laboratoire de Neurosciences Cognitives et Computationnelles, INSERM, Paris, France

**Keywords:** Human behaviour, Cognitive neuroscience, Learning algorithms, Human behaviour

## Abstract

Social media have fundamentally transformed how we live and communicate. However, the methods to study how our cognitive systems interact with technology platforms are very limited. Computational modelling represents a new avenue to uncover the finegrained cognitive processes driving social media behaviour. Here, we develop a computational model of real-world social media posting data, adapted from the animal reward learning literature. Using a Twitter (currently X) dataset (*n* = 2696 users), including a preregistered replication, we show that a hybrid reinforcement learning and habitual cognitive process underlies social media posting behaviour. More frequent posters show more signs of habitual behaviour. Further, younger people and women are more driven by reinforcement learning – updating their strategy more adaptively to maximise social media rewards – while older users and men are more habitual.

## Abstract (web summary)

The cognitive processes driving social media use could be key to understanding social media’s impact. Here, the authors develop a computational model of real-world social media behaviour, identifying multiple cognitive processes underlying posting.

## Introduction

The advent of social media has radically transformed both our personal and political worlds. UK adults now spend, on average, over a whole day a week online^1^, while large social media platforms have been accused of influencing wellbeing, political polarization and even election outcomes^2,3^. Such changes have triggered concerns about the role of social media in wellbeing and political crises—yet the ensuing search for scientific evidence has been inconclusive^4–6^.

This lack of scientific progress can largely be attributed to methodological issues with existing research^4,7^. Traditional research characterizes social media use as a new and unprecedented phenomenon, often searching for a dose-response relationship between time spent on social media and outcomes of interest, such as wellbeing or polarization^5,8,9^. However, this line of research has yielded mixed and inconsistent results^5,10^. Critically, the dose-response approach cannot account for the diversity of social media users and behaviour^7^. Further, social media fluctuate in platform and algorithm design on a much faster timescale than typical scientific research^4,10–12^. Therefore, dose-response approaches which only test “doses” of specific social media iterations will not generate findings that are robust to these changes.

An alternative perspective applies a cognitive lens to understand social media use. On this view, social media behaviour is not a rootless, isolated phenomenon: instead, it is driven by a cognitive process that has been well-characterized by decades of behavioural science research. What is unprecedented is the novel environmental context with which this cognitive process interacts^4^.

Taking this cognitive perspective, recent theoretical work suggests that social media might hijack human learning processes developed across evolution^13–17^. For example, features such as Likes change how we are rewarded, potentially altering reward-seeking behaviour and even desires^18–20^. Such features could therefore produce a behaviour-environment “mismatch”, wherein human behavioural tendencies which were beneficial in the environments we evolved in become vulnerabilities in evolutionarily novel environments. For example, the human preference for low-effort rewards might have originally evolved to help conserve energy, but could become a vulnerability when online rewards, such as Likes are significantly lower effort to give and receive than many offline rewards^13^. This behaviour-environment mismatch could therefore drive excessive social media use, with downstream impacts on individuals and society.

If social media use is an inherently cognitive phenomenon, it can be studied using cognitive computational models from psychology and neuroscience. These models are sets of mathematical equations which specify the latent cognitive processes converting information from input (e.g., perception) to output (e.g., actions)^13^. We can use computational models to infer the underlying cognitive processes which produce observable behaviour: for example, whether actions are more repetitive, fixed and habitual, or more adaptable in pursuit of rewards. This could provide new ways to tackle open questions about social media use, by formalising psychological theories into precise equations which make quantitative and qualitative predictions about observable data.

One such open question regards what drives frequent social media use. Cognitive research has established that human reward-seeking behaviour arises from the interaction of multiple cognitive systems. These cognitive systems include a model-free reinforcement learning (RL) system, which learns by trial and error to maximise long-term reward^22^, as well as a habitual system, which simply persists with actions similar to previous actions^23–25^. Habitual behaviour occurs when an originally rewarded action is repeated so many times that it becomes automatic, persisting even when in conflict with explicit goals^25–28^. Features of social media, such as intermittent rewards and the “infinite scroll”, might disproportionately increase reliance on the habitual system^13,29^. Previous research suggests that some social media users are more habitual, but has not, to our knowledge, extracted the relative contribution of the habitual system within each user^18,30^. In contrast, computational modelling could continuously quantify the contribution of the habit system for each individual, allowing more granular analysis of how habits change across time and environments.

A cognitive approach might also help answer questions about why social media use and its impacts are not uniform across people. For example, older people are more susceptible to sharing obviously false news^31^, adolescents are more likely to experience lower life satisfaction after social media use^32^, and those with lower wellbeing use social media more often^32^. One possibility is that aspects of cognitive processes, such as reliance on habits, which also vary with demographics and wellbeing, underlie these population differences in social media use^33–35^. To address this question with computational modelling, we could extract parameters summarizing each individual’s social media behavioural phenotype, and compare these phenotypes across demographic populations^36,37^.

While a better understanding of cognitive processes on social media has enormous potential, there is a lack of effective methods to characterize them in real-world behaviour. Most current work investigates reward learning via observable statistical signatures^20,38,39^. For instance, previous research revealed that receiving more Likes on one’s recent posts predicts an increased number of posts on the subsequent day, consistent with a reward learning process whereby social media rewards invigorate subsequent behaviour^20,38^. However, there are many ways in which this statistical signature might be operationalized, making it difficult to establish which signatures truly reflect the hypothesized generative process. For example, “rewards received” could be quantified using absolute rewards on a previous post^30^, or the average number of rewards received across posts on a previous day^20^. Simulating cognitive computational models can overcome this limitation by identifying which specific statistical signatures are produced (or not) by a given underlying process^40^.

However, computational models are typically used to describe tailored experimental data, making it difficult to translate insights to real-world behaviour. Although less ecologically valid, modelling of experimental data is made possible by careful experimental designs with controlled, well-differentiated experimental conditions. In contrast, real-world data is generated in complex environments, wherein researchers cannot control the amount or format of data, nor the environmental conditions.

Despite the challenge of adapting models to such messy real-world contexts, recent studies have begun to fit cognitive computational models directly to naturalistic social media data for the first time^21,36,41^. A pioneering study demonstrated that Instagram posting behaviour was well-characterized by model-free RL, a strategy by which humans and animals learn by trial and error to maximise long-term reward^21^. However, other research suggests that this reward-sensitive learning process does not fully explain social media posting. Indeed, more frequent social media posters, who also self-report more habitual social media use, are less likely to respond to changes in rewards via RL^30^. This is consistent with the influence of a perseverative, habitual behavioural system underlying frequent social media use. Thus, social media posting may be better characterised by the interaction of multiple behavioural systems.

**Fig. 1:**
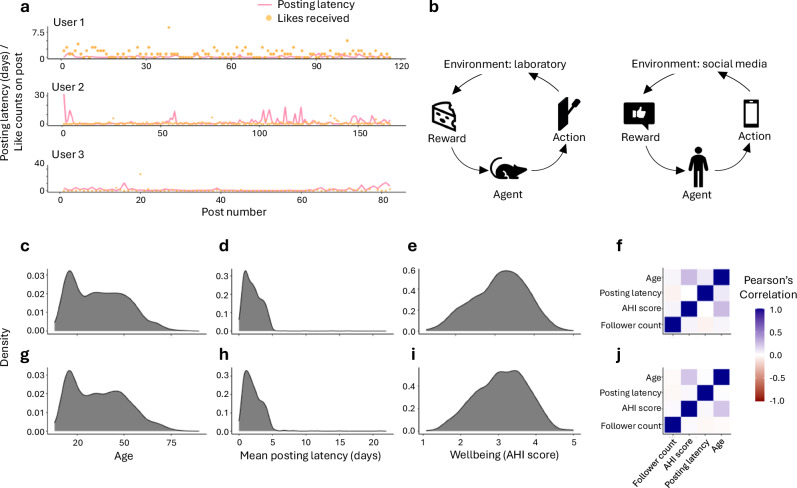
Descriptive statistics and modelling procedure. AHI Authentic Happiness Inventory. **a** Time course for posting latencies and Likes received per post, for three example users from the confirmatory sample. The *y*-axis reports both posting latency and Like counts for each post, while the *x*-axis reports the consecutive post number in the dataset (e.g., “1” on the *x*-axis is a user’s first post, “2” is their second post, etc.). **b** Schematic diagram showing how the basic modelling procedure is adapted from the case of laboratory animal reward experiments to social media. In traditional experiments, reward learning models describe how a rat (agent) adjusts its speed of lever pressing (action) in the laboratory (environment), in order to maximize the food it receives (reward). In contrast, our reward learning models of social media use characterize how a social media user (agent) adjusts their speed of posting (action) on social media (environment) in order to maximize social media Likes (reward). **c** Distribution of age in the discovery sample after removing outliers for the age analyses (see “Methods”). **d** Distribution of mean posting latency in the discovery sample (*n* = 1138). **e** Distribution of wellbeing scores in the discovery sample. **f** Correlations between variables in the discovery sample after removing outliers for age analyses. **g** Distribution of age in the confirmatory sample after removing outliers for the age analyses (see “Methods”). **h** Distribution of mean posting latency in the confirmatory sample (*n* = 1558). **i** Distribution of wellbeing scores in the confirmatory sample. **j** Correlations between variables in the confirmatory sample after removing outliers for age analyses.

To capture this multi-system process and study how it varies across users, here we build a dual-system generative model of real-world social media behaviour, with both model-free RL and habits. We adapt this model directly from classical psychology models of how laboratory rats adjust lever-pressing speed to maximize rewards such as food consumption^42,43^ (Fig. 1b).

By fitting this model instead to real-world social media data, wherein social media “rewards” such as Likes modulate the speed of posting, we demonstrate how evolutionarily-old learning processes drive social media use. Importantly, this computational model of real-world data allows us to quantify the relative contributions of the model-free RL vs. habitual system for each individual, as well as to test for the presence of a “positivity bias”, the well-documented propensity to learn at a higher rate from information which is more rewarding relative to expectations^44^.

To demonstrate the potential of this model to address open questions about social media use, we analyse a large dataset of Twitter (currently X) posting behaviour, divided into a discovery sample (*n* = 1138), and then a preregistered confirmatory sample (*n* = 1558). We first compare seven alternative models to establish the underlying cognitive process driving social media posting across users. We then examine how parameters of the winning model relate to individual differences in frequency of posting, as we hypothesize that habit drives more frequent use. Finally, we relate the modelled cognitive process to age, gender and wellbeing across individuals, to test the hypothesis that reward learning strategies relate to these populations’ differences in modes of social media use.

## Results

### A hybrid reward-sensitive reinforcement learning and perseverative habitual process underlies Twitter (X) posting behaviour

Our first aim was to establish the cognitive process driving Twitter (currently X) posting. Specifically, we were interested in whether and how the reward received on each post (i.e., the number of Likes) moderated the latency between posts (i.e., the time taken to post after the preceding post). We obtained a Twitter (currently X) dataset with *n* = 2696 users, with linked questionnaire data detailing self-reported date of birth, gender and wellbeing (as indicated by scores on the Authentic Happiness Inventory (AHI)^45^; see “Methods”). We selected a random sample as the discovery sample for exploratory analyses (*N* = 1138 after pre-processing). We then preregistered our analysis pipeline^46^, and subsequently repeated our analyses on the confirmatory sample (*N* = 1558 after pre-processing). Figure 1c–i and Table 1 show descriptive statistics for the final discovery and confirmatory samples (after exclusions; see “Methods”).

**Table 1:** Descriptive statistics for the discovery sample and confirmatory sample

Variable	Sample	Minimum	Maximum	Mean	Median	Standard deviation
Age	Discovery	15	82	36	35	14
	Confirmatory	13	80	36	35	14
Mean posting latency (days)	Discovery	0.04	16.21	2.10	1.92	1.35
	Confirmatory	0.07	21.03	2.12	1.86	1.42
Standard deviation of posting latency (days)	Discovery	0.06	110.82	3.98	3.00	6.02
	Confirmatory	0.13	231.84	4.76	2.94	11.25
Follower number	Discovery	0	40,485,990	38,143	390	1,200,326
	Confirmatory	0	17,240,910	32,638	350	607,397
Mean Likes per post within person	Discovery	0	11,495	16.77	1.18	350.78
	Confirmatory	0	32,239	37.18	1.03	885.43
Standard deviation of Likes per post within person	Discovery	0	19,075.57	36.48	2.21	632.69
	Confirmatory	0	24,036.64	55.11	1.97	961.69
Number of posts per person	Discovery	80	2437	260.18	185.00	216.55
	Confirmatory	80	1950	249.59	185.50	199.51

**Fig. 2:**
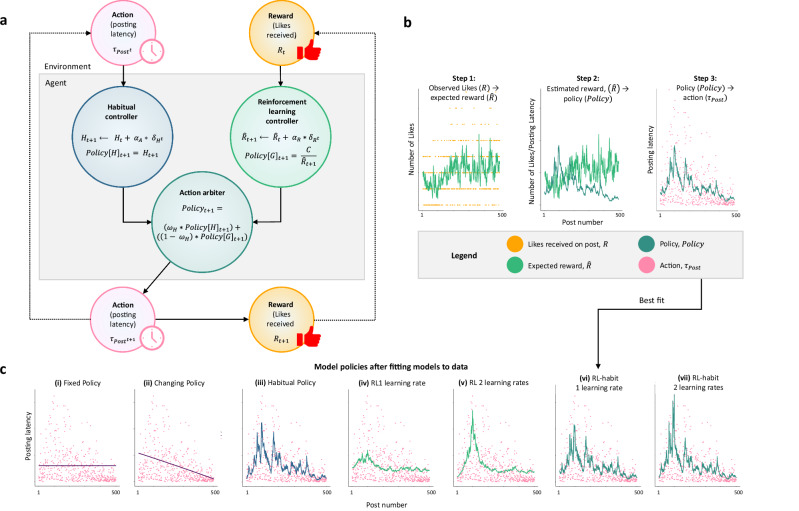
Schematic diagram and simulations to explain the computational models. **a** A schematic diagram of the RL-habit model, which is adapted from animal reward learning models in references^44,48,71^. Details of the model derivation are contained in the Supplementary Material (Supplementary Methods). The “habitual controller”, which is equivalent to the Habitual Policy model, calculates a policy based on the history of previous actions. The “reinforcement learning controller”, which is equivalent to the pure RL model, calculates a policy based on the history of previous rewards. The “action arbiter” calculates a final policy based on a paramaterised weighting of the habitual and reinforcement learning policies, which probabilistically determines the subsequent action (posting latency). A reward is received for this action, and this action and reward then feed into the calculation of the next policy. **b** In a dataset of 500 posts simulated for a single user using the RLH1 model, the conversion from observed rewards, to latent variables of estimated reward and policy, and finally to observable actions based on the policy, is shown. At Step 1, the expected reward is estimated from the history of observed Likes. At Step 2, this is integrated with the habitual controller and converted into a final policy, which qualitatively looks like a smoothed inverse version of the expected reward, where the habitual component smooths the policy across time. The policy is higher (longer latency) when expected reward is lower. Finally at Step 3, actions are drawn probabilistically from the policy to produce the observable data. **c** All seven models were fitted back to the RLH1 simulated dataset. With each model’s best-fitting parameters, the latent policy variable inferred using that model is shown. The RLH1 model provides the best fit to the data (which is as expected, given that this was the model used to simulate the observable data). We see that the recovered hypothesised policy for RLH1 is comparable to the underlying simulated policy shown in b.

Figure 1a shows the observable data from three example users. To establish the process underlying users’ posting, we constructed seven computational models, each representing a candidate underlying process producing the observable data (Fig. 2c, “Methods”). Each model describes an alternative underlying generative process determining the posting latency for each post. The models include baseline models, which summarize data trends without modelling generative cognitive processes; habit models, which describe how past behavioural context predicts each action; and reward learning models, which describe how rewards received in the past (e.g., Likes) modulate subsequent actions. Hybrid RL-habit models include both habit and reward learning components.

The two baseline models, Fixed Policy (Model 1: FP) and Changing Policy (Model 2: CP), represent either a constant posting latency or a gradually changing posting latency, respectively, with no hypothesised associated cognitive mechanism. Social media users following these models will post at a specific rate that is either always the same (FP) or gradually increasing or decreasing over time (CP) (Fig. 2c(i-ii)). In contrast, the Habitual Policy model (Model 3: HP) formalises the hypothesis that social media posting latency is governed by a habitual process. The habitual process takes the form of perseveration or autocorrelation across average previous actions^47,48^. The model repeats an average posting latency similar to the average posting latency in the recent past, and, unlike RL models, does not change posting latency policy in response to changes in reward (Fig. 2c(iii)).

A pure RL model (Model 4: RL1) instantiates a reward-sensitive process, where posting latency policy is determined by the expected reward rate, and the agent posts more frequently when expected reward is higher. In practice, this means that posting latency on average will be lower when more rewards (Likes) have been received in the recent past (Fig. 2(iv)). A dual RL model (Model 5: RL2) uses the same baseline model as RL1 but estimates the expected reward with a valence bias (i.e., learning at different reward learning rates when the reward is more vs. less than expected) (Fig. 2(v)). Finally, two hybrid RL-Habit models (Model 6: RLH1; Model 7: RLH2) constitute multi-process models wherein a model-free RL system and a habitual system jointly contribute to behaviour (Fig. 2a–c(vi-vii)). Similar to RL2, RLH2 additionally has a valence bias in the RL component.

We preregistered that our effects of interest would be considered true if either (a) the effect is significant in the confirmatory sample, or (b) the effect is significant in a fixed-effects meta-analysis across discovery and confirmatory samples (https://osf.io/jybhx)^46^. We therefore report all statistics for the confirmatory sample and additional meta-analyses where appropriate.

We used both model comparison and model falsification to select the winning model^40^. Model comparison assesses the “predictive performance” of the model—its ability to predict the observed data^49^. However, this process is necessarily relative: it reveals the best model out of the set of models considered. In contrast, model falsification functions as an absolute rejection criterion for model selection^40^.

This assesses the “generative performance” of a model: its ability to account for a specific statistical signature of interest when simulated^40,49^. In particular, we use RL models to simulate data and show that after a positive reward prediction error (operationalized as being when Likes on a post are greater than on the posts leading up to it), RL models systematically choose to take a shorter time before posting again (i.e., decrease their posting latency relative to their previous policy). However, this statistical signature is not present in datasets simulated using models without an RL component (for example, the HP model, where Likes on a post have no impact on posting latency). If this statistical signature is present in the empirical data, this falsifies the hypothesis that a process without an RL component underlies the data, regardless of how well such models perform in model comparison.

### Model comparison

**Fig. 3:**
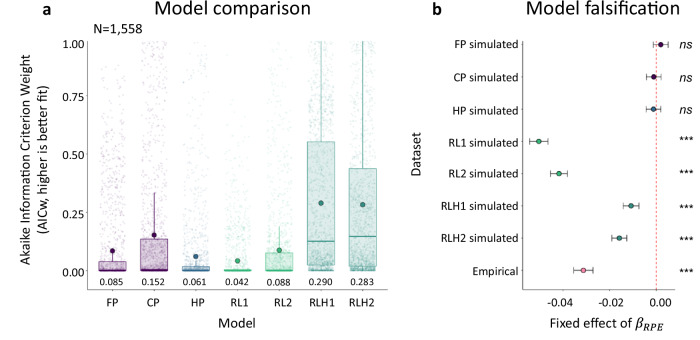
Model selection characterises the underlying learning process across users. **a** Model comparison in the confirmatory sample (*n* = 1558). FP Fixed Policy, CP Changing Policy, HP Habitual Policy, RL1 Pure RL, single learning rate, RL2 Pure RL, double learning rate, RLH1 Hybrid RL-habit, single learning rate, RLH2 Hybrid RL-habit, double learning rate. Each model was fitted to each user and a metric of model fit, the Akaike Information Criterion weight (AICw), was derived. Higher Akaike Information Criterion weight (AICw) represents a better fit. Scattered points represent the AICw results of the model fit for each user, *n* = 1558 for each model listed along the *x*-axis. Outlined circles represent the mean AICw, which is also indicated as numbers along the top of the *x*-axis (the criterion for which best-fitting model was selected). Box plots represent the distribution of AICw values. In each box plot, the centre line indicates the median (50th percentile), the box bounds indicate the 25th and 75th percentiles, and whiskers extend to the most extreme values within 1.5 times the interquartile range for AICw. Model comparison reveals that RLH1 provided the best description of behaviour, as it has the highest mean AICw across users. **b** Model falsification reveals that an RL component is present in the empirical confirmatory sample (*n* = 1558). All datasets were simulated with the same number of users and empirical parameters as the empirical confirmatory dataset (*n* = 1558). To establish the predictive coefficient, ${\beta }_{{RPE}}$, wherein reward prediction error on each post predicted change in posting latency on the subsequent post, we fitted multilevel models to each simulated and empirical dataset, with users as random slopes. Dots show estimates of the fixed effect of ${\beta }_{{RPE}}$ across the dataset, error bars show 95% confidence intervals for these fixed effects estimates, and the column on the right indicates significance for two-tailed significance tests, according to the following scheme: *ns* not significant, * = *p* < 0.05, ** = *p* < 0.01, *** = *p *< 0.001. This analysis was exploratory and not preregistered, and corrections were not made for multiple comparisons. Simulated datasets without an RL component do not show a significantly negative fixed effect of ${\beta }_{{RPE}}$ (FP: *t*(387300) = 1.203, *p* = 0.229, *β* = 0.002, 95% CI = [−0.001, 0.005], CP: *t*(387300) = −0.656, *p* = 0.512, *β* = −0.001, 95% CI = [−0.004, 0.002], HP: *t*(387300) = −0.726, *p* = 0.468, *β* = 0.001, 95% CI = [−0.004, 0.002]). In contrast, the simulated datasets with an RL component do (RL1: *t*(1063) = −25.054, *p* < 0.001, *β* = −0.050, 95% CI = [−0.054, −0.046]; RL2: *t*(1069) = −22.534, *p* < 0.001, *β* = −0.041, 95% CI = [−0.045, −0.038]; RLH1: *t*(920) = −6.268, *p* < 0.001, *β* = −0.011, 95% CI = [−0.014, −0.007]; RLH2: *t*(909.6) = −9.65, *p* < 0.001, *β* = −0.016, 95% CI = [−0.019, −0.013]). As the empirical dataset also shows a significantly negative fixed effect of ${\beta }_{{RPE}}$ (*t*(1276) = −14.864, *p* < 0.001, *β* = −0.031, 95% CI = [−0.035, −0.027]), this falsifies the hypothesis that the FP, CP or HP models underlie the data.

For model comparison, we preregistered that the model with the highest mean Akaike Information Criterion weight (AICw) would be the “winning” model^50^. Across both discovery and confirmatory samples, the hybrid RL-Habit models provided the best description of behaviour, supporting the hypothesis that RL and habit both contribute to posting behaviour (Fig. 3a). Specifically, RLH1 (hybrid RL-habit with no valence bias) was the best-fitting model in the AH confirmatory sample. RLH2 was the winning model in the AH discovery sample. The inconsistency across samples in whether the winning model had a valence bias likely reflects individual variation across users in whether they were better fit by RLH2 or RLH1. However, overall, given that the RLH1 model wins in the confirmatory sample, we can conclude that there is little evidence for a valence bias (or specifically for a positivity bias). We specified in the preregistration that if a different model from RLH2 provided the best fit in the confirmatory sample, we would report results both for RLH2 and for the winning model. We therefore report all results for parameters fit with the winning model in the confirmatory sample, RLH1, in the main body of the paper. We report results for parameters fit with RLH2 in the Supplementary Material (Supplementary Note 3). Given that RLH2 is equivalent to RLH1 in the special case where the positive and negative reward learning rates are equal, key results do not differ substantially between the models.

We then conducted several sensitivity analyses to verify the robustness of this model comparison result. First, we investigated the robustness to different definitions of reward. All models defined reward as the number of Likes. To test this assumption, we conducted additional analyses where reward was quantified either as only Retweets or as the sum of Likes and Retweets on each post (see Supplementary Material and Supplementary Note 3). These sensitivity analyses confirmed that hybrid RL-habit models also fitted data best under these different operationalizations of reward. Thus, the model comparison result is not dependent on a specific definition of what constitutes a reward on Twitter (currently X). Additional sensitivity analyses confirmed that the result that RL-habit models won was robust to variations of other analytical decisions in modelling, including different initializations of internal variables and different probabilistic distributions linking internal policy to action (Supplementary Material and Supplementary Note 3).

Finally, we conducted a further model comparison analysis in the confirmatory sample, comparing each of our models to the previous RL model of social media data developed by Lindström et al.^21,36^ (Supplementary Material and Supplementary Note 3). Both of our RL-habit hybrid models, RLH1 and RLH2, provided a better fit than the RL model developed by Lindström et al.^22^

### Model falsification

Next, for model falsification, we identified a behavioural signature of reward learning. Specifically, we hypothesized that in RL models, a positive reward prediction error on a post (i.e., where more Likes are received than expected based on the recent history of Likes) will systematically cause the subsequent post to occur more quickly in time (i.e., a decrease in posting latency compared with the previous posting latency). This is because in the RL model, an increase in expected reward leads to a decrease in policy (Fig. 2b, “Methods”). The decreased internal policy then has a probabilistic decreasing effect on observable posting latency. To quantify this statistically, we used the regression coefficient ${\beta }_{{RPE}}$ denoting the predictive relationship between the reward prediction error on a post and the change in posting latency on the subsequent post (“Methods”). Although we included this signature in our preregistration, we did not preregister the model falsification procedure, so it should be understood as exploratory (see the Preregistration Deviation table in the Supplementary Material and Supplementary Note 4).

We simulated datasets for each of the seven computational models using the exact parameters fitted for each of the users in the confirmatory sample. We reasoned that simulated datasets generated by models with an RL component (i.e., the pure RL and hybrid RL-habit models) would exhibit the reward learning signature, while simulated datasets generated by models without an RL component (i.e., the FP, CP and HP models) would not. Given that a hybrid RL-habit model won in model comparison in the empirical data, we also expected the empirical dataset to exhibit the reward learning signature, which would falsify the hypothesis that the FP, CP or HP models underlie behaviour. We therefore examined the reward learning behavioural signature (fixed effect of ${\beta }_{{RPE}}$) within each of the simulated datasets, and then the confirmatory empirical dataset (Fig. 3b).

As expected, ${\beta }_{{RPE}}$ was not significantly different from 0 in the datasets simulated by models without RL components (FP simulated dataset: *t*(387300) = 1.203, *p* = 0.229, *β* = 0.002, 95% CI = [−0.001, 0.005]; CP simulated dataset: *t*(387300) = −0.656, *p* = 0.512, *β* = −0.001, 95% CI = [−0.004, 0.002]; HP simulated dataset: *t*(387300) = −0.726, *p* = 0.468, *β* = 0.001, 95% CI = [−0.004, 0.002]). In contrast, the fixed effects of ${\beta }_{{RPE}}$ for the models with an RL component were significantly different from 0 (RL1 simulated dataset: *t*(1063) = −25.054, *p* < 0.001, *β* = −0.050, 95% CI = [−0.054, −0.046]; RL2 simulated dataset: *t*(1069) = −22.534, *p* < 0.001, *β* = −0.041, 95% CI = [−0.045, −0.038]; RLH1 simulated dataset: *t*(920) = −6.268, *p *< 0.001, *β* = −0.011, 95% CI = [−0.014, −0.007]; RLH2 simulated dataset: *t*(909.6) = −9.650, *p* < 0.001, *β* = −0.016, 95% CI = [−0.019, −0.013]). Importantly, ${\beta }_{{RPE}}$ was also significantly different from 0 in the empirical confirmatory dataset (*t*(1276) = −14.864, *p* < 0.001, *β* = −0.031, 95% CI = [−0.035, −0.027]), falsifying the hypothesis that reward learning is not occurring.

We also note that the size of the fixed effects estimate in the data seems to be in between the pure RL and RL-habit models, rather than the same as the RL-habit models, as we might expect if the RL-habit models fully capture all RL in the data. This suggests that the RL-habit models underestimate the strength of RL in the data, consistent with the possibility that our simplified model (for example, only capturing model-free RL on a ratio schedule) does not fully capture the more complex RL process underlying social media posting (which could include, for example, interval-schedule model-free RL, model-based RL or other RL systems)^13^.

Together, model comparison and model falsification indicate that social media posting is characterised by the integration of different cognitive systems (e.g., RL combined with habit) rather than a purely RL or purely habitual system.

### Higher posting frequency is related to higher habit weight and lower action learning rate, in line with habitual behaviour

Our next aim was to identify whether posting frequency, which is often used as a proxy for habitual social media use^30,51^, relates to model parameters suggestive of habitual behaviour. We identified three parameters from the winning model, RLH1, which might provide converging behavioural signatures of habit. The first signature of habit strength is a higher habit weight parameter. Higher habit weight indicates less weight placed on the RL system, which has been posited as the computational instantiation of habit^47^. Secondly, habits have been associated with predictability of behaviour across time^52,53^. The action learning rate parameter models the length of history of recent actions that predict behaviour, with a lower action learning rate parameter indicating a longer history of consistent average posting latency across time, consistent with habit-like behaviour that does not adapt to changing reward contingencies. Finally, habits are associated with decreased sensitivity to reward contingencies^25^. Therefore, a lower reward learning rate, representing a lower rate of adapting behaviour to experienced reward, might reflect more habitual use^30^.

**Fig. 4:**
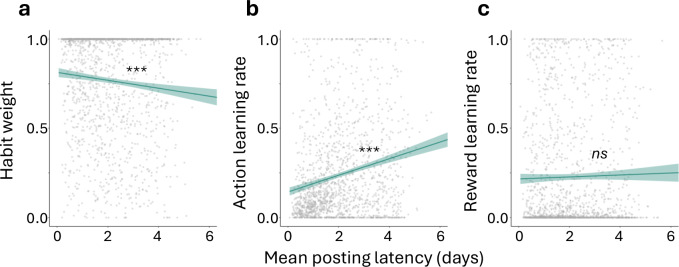
Relationship between posting latency and RLH1 parameters suggestive of habitual behaviour. All results are shown and calculated for the confirmatory dataset (*n* = 1558). Scatter plots show the relationship between posting latency and an RLH1 parameter for each user. Annotations indicate significance, according to the following scheme: *ns* not significant, * = *p* < 0.05, ** = *p* < 0.01, *** = *p* < 0.001. All statistical tests were preregistered and adjustments were not made for multiple comparisons. **a** Plotted lines are lines of best fit, shaded areas represent 95% confidence intervals. Testing with linear regression, posting latency negatively predicted habit weight (*t*(1556) = −4.284, *p*(two-tailed) <0.001, *β* = −0.108, 95% CI = [−0.157, −0.059]). **b** Plotted lines are lines of best fit, shaded areas represent 95% confidence intervals. Testing with linear regression, posting latency positively predicted action learning rate (*t*(1556) = 9.955, *p*(two-tailed) <0.001, *β* = 0.245, 95% CI = [0.196, 0.293]). **c** Plotted lines are lines of best fit, shaded areas represent 95% confidence intervals. Testing with linear regression, posting latency did not significantly predict reward learning rate (*t*(1556) = 0.948, *p*(two-tailed) = 0.343, *β* = 0.024, 95% CI = [−0.026, 0.074]).

More frequent posters, i.e., people with lower posting latency, exhibited converging computational signatures of more habitual behaviour. As predicted in the preregistration, users with lower posting latency had a higher habit weight (*t*(1556) = −4.284, *p* < 0.001, *β* = −0.108, 95% CI = [−0.157, −0.059]) (Fig. 4a) and a lower action learning rate in the confirmatory sample (*t*(1556) = 9.955, *p* < 0.001, *β* = 0.245, 95% CI = [0.196, 0.293]) (Fig. 4b). To assess the possibility that these parameters were mutually dependent due to the modelling pipeline rather than independent features of the empirical data, we validated using parameter recovery that there was no evidence that fitted habit weight and action learning rate were (anti-)correlated in simulated uncorrelated data (Supplementary Material and Supplementary Note 2). In contrast to habit weight and action learning rate, reward learning rate did not significantly relate to posting latency (*t*(1556) = 0.948, *p* = 0.343, *β* = 0.024, 95% CI = [−0.026, 0.074]) (Fig. 4c). This relationship remained nonsignificant after conducting a preregistered fixed effects meta-analysis across the discovery and confirmatory samples (*z* = 0.578, *p* = 0.563, *β* = 0.011, 95% CI = [−0.027, 0.049]).

Sensitivity analyses confirm that these findings remain significant after removing posting latency outliers (Supplementary Material and Supplementary Note 3) as well as after controlling for number of posts per user (see Preregistration Deviations in the Supplementary Material and Supplementary Note 4). Specifically, after conducting multiple linear regression controlling for number of posts per user, partial regression coefficients indicated that users with lower posting latency still had a higher habit weight (partial regression coefficient for posting latency: *t*(1555) = −2.197, *p* = 0.028, *β* = −0.070, 95% CI = [−0.132, −0.007], partial regression coefficient for number of posts: *t*(1555) = 2.004, *p* = 0.045, *β* = 0.063, 95% CI = [0.001, 0.126]) (Fig. 4a) and a lower action learning rate (partial regression coefficient for posting latency: *t*(1555) = 5.802, *p* < 0.001, *β* = 0.179, 95% CI = [0.118, 0.239], partial regression coefficient for number of posts: *t*(1555) = −3.541, *p* < 0.001, *β* = −0.109, 95% CI = [−0.169, −0.049]) (Fig. 4b). The relationship between posting latency and reward learning rate remained not significant (partial regression coefficient for posting latency: *t*(1555) = 0.518, *p* = 0.605, *β* = 0.017, 95% CI = [−0.046, 0.079], partial regression coefficient for number of posts: *t*(1555) = −0.390, *p* = 0.697, *β* = −0.012, 95% CI = [−0.075, 0.050]) (Fig. 4c).

However, we note that posting latency did positively correlate with negative reward learning rate extracted from the RLH2 model (*t*(1556) = 2.800, *p* = 0.005, *β* = 0.071, 95% CI = [0.021, 0.120]), suggesting that more frequent posters on Twitter (currently X) are less likely to change their behaviour when they receive fewer Likes than they expected (Supplementary Material and Supplementary Note 3). This correlation remained significant after controlling for number of posts, using the preregistered one-tailed significance test (partial regression coefficient for posting latency: *t*(1555) = 1.695, *p*(one-tailed) = 0.045, *β* = 0.054, 95% CI = [−0.008, 0.116], partial regression coefficient for number of posts: *t*(1555) = −0.877, *p* = 0.381, *β* = −0.028, 95% CI = [−0.090, 0.034]).

Overall, users with higher posting frequency therefore exhibited three hypothesized computational signatures of more habitual use—higher habit weight, higher action learning rate and lower negative reward learning rate—although there is no evidence that they had a lower overall reward learning rate. In other words, high frequency posters had more perseverative behaviour (higher habit weight), with less reward-independent volatility (lower action learning rate), and reduced behavioural sensitivity to negative reward prediction error (lower negative reward learning rate). Together, these characteristics of frequent posting suggest habitual behaviour.

### Younger people and women are more driven by reinforcement learning when posting on Twitter (currently X)

**Fig. 5:**
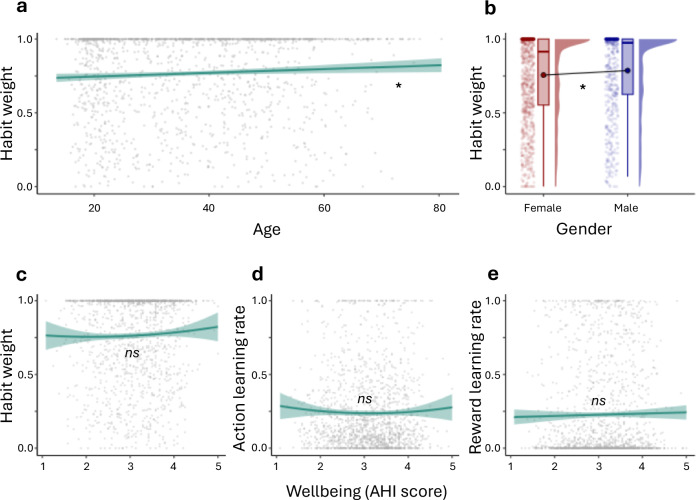
Relationship between demographics and wellbeing, and RLH1 parameters. All statistics are calculated for the confirmatory dataset (*n* = 1558). Annotations indicate significance, according to the following scheme: *ns* not significant, * = *p* < 0.05, ** = *p* < 0.01, *** = *p* < 0.001. All statistical tests were preregistered and adjustments were not made for multiple comparisons. **a** Plotted lines are lines of best fit, shaded areas represent 95% confidence intervals. Testing with linear regression, age positively predicted habit weight (*t*(1526) = 2.442, *p*(two-tailed) = 0.015, *β* = 0.062, 95% CI = [0.012, 0.113]). **b** Dots represent habit weight for each individual user. Box plots represent the distribution of habit weight values for each gender. In each box plot, the centre line indicates the median (50th percentile), the box bounds indicate the 25th and 75th percentiles, and whiskers extend to the most extreme values within 1.5 times the interquartile range for habit weight. Half violin plots represent density. Outlined circles represent mean values of habit weight for each gender. Testing with a paired-samples *t*-test, males (*n* = 560) had a higher habit weight than females (*n* = 998) (*t*(1182.2) = −1.96, *p*(one-tailed) = 0.025, *d* = −1.03, 95% CI = [−0.206, 0.001]). **c** Plotted lines are lines of best fit, shaded areas represent 95% confidence intervals. Testing with linear regression, wellbeing had no significant linear or quadratic relationship with habit weight (linear: *t*(1555) = −0.994, *p* = 0.321, *β* = −0.003, 95% CI = [−0.053, 0.047]; quadratic: *t*(1555) = 0.969, *p* = 0.333, Cohen’s ${f}^{2}$ = 0.001, 95% CI (bootstrapped) = [0.000, 0.006]). **d** Plotted lines are lines of best fit, shaded areas represent 95% confidence intervals. Testing with linear regression, wellbeing had no significant linear or quadratic relationship with action learning rate (linear: *β* = −0.07, *p*(two-tailed) = 0.321; quadratic: *β* = 0.012, *p*(two-tailed) = 0.333). **e** Plotted lines are lines of best fit, shaded areas represent 95% confidence intervals. Testing with linear regression, wellbeing had no significant linear relationship with reward learning rate *t*(1556) = 0.651, p(two-tailed) = 0.515, *β* = 0.016, 95% CI = [−0.033, 0.066].

We were next interested in how age and gender predicted individual differences in the contribution of RL vs. habitual systems. To investigate the contribution of different cognitive processes, we related age and gender to the size of the habit weight parameter. Given that a lower habit weight indicates more reliance on pure RL, we interpret this as more driven by RL. As hypothesized in the preregistration, age was positively associated with habit weight (*t*(1526) = 2.442, *p* = 0.015, *β* = 0.062, 95% CI = [0.012, 0.113]) (Fig. 5a). This is also supported by our finding that younger people exhibited a stronger statistical signature of RL (Supplementary Material and Supplementary Note 2). Additionally, using our preregistered one-tailed significance test, men had a higher habit weight than females (paired-samples *t*-test: *t*(1182.2) = −1.96, *p*(one-tailed) = 0.025, *d* = −1.03, 95% CI = [−0.206, 0.001]) (Fig. 5b).

Sensitivity analyses confirmed that these results remained significant after controlling for number of posts per user (see Preregistration Deviations in the Supplementary Material and Supplementary Note 4). Specifically, after conducting multiple linear regression, partial regression coefficients indicated that age significantly predicted habit weight (partial regression coefficient for age: *t*(1525) = 2.298, *p* = 0.022, *β* = 0.058, 95% CI = [0.009, 0.108], partial regression coefficient for number of posts: *t*(1525) = 4.185, *p* < 0.001, *β* = 0.011, 95% CI = [0.057, 0.156]). Additionally, after conducting multiple linear regression using gender and number of posts to predict habit weight, and using our preregistered one-tailed significance test, partial regression coefficients indicated that males had a higher habit weight than females (partial regression coefficient for gender: *t*(1555) = 2.048, *p*(one-tailed) = 0.020, *β* = 0.107, 95% CI = [0.005, 0.210], partial regression coefficient for number of posts: *t*(1555) = 4.322, *p*(one-tailed) <0.001, *β* = 0.107, 95% CI = [0.057, 0.156]).

Overall, these results show that younger people and women have a greater influence of RL, or are more reward-sensitive, when posting on Twitter (currently X). In other words, these populations are more likely to actively adapt their posting behaviour to maximize social media rewards, while older people and men are more likely to follow a reward-independent posting strategy suggestive of habit. Thus, differences in the contribution of RL vs. habits could be a candidate cognitive mechanism underlying age and gender differences in social media use.

### No evidence for a relationship between wellbeing and reward learning on social media

Finally, we were interested in how different cognitive processes and biases relate to positive wellbeing across social media users (Fig. 5c–e). First, we investigated whether the reliance on RL vs. habitual social media use, which we found to differ across age and gender, related to wellbeing. Because we had strong prior hypotheses that wellbeing would relate to reward learning based on previous literature^20,54,55^, we explored for nonlinear as well as linear relationships in the discovery sample. We found nonlinear (i.e., both linear and quadratic) relationships between wellbeing and both habit weight and action learning rate in the discovery sample. Therefore, to see if these quadratic relationships replicated, we here tested for linear and quadratic relationships between wellbeing and both habit weight and action learning rate, and just for a linear relationship between wellbeing and reward learning rate.

To this end, we related the habit weight and action learning rate to scores on the AHI, where higher AHI scores indicate greater wellbeing. We found no significant linear or quadratic relationship between wellbeing and habit weight (linear: *t*(1555) = −0.431, *p* = 0.667, *β* = −0.037, 95% CI = [−0.013, 0.087], quadratic: *t*(1555) = 0.622, *p* = 0.534, Cohen’s ${f}^{2}$ = 0.000, 95% CI (bootstrapped) = [0.000, 0.004]) (Fig. 5c). We therefore conducted a preregistered fixed-effects meta-analysis across discovery and confirmatory samples which also found no significant relationship (linear: *z* = 1.231, *p* = 0.219, *β* = 0.024, 95% CI = [−0.014, 0.062]; quadratic: *z* = 0.709, *p* = 0.478, Cohen’s ${f}^{2}$ = 0.002, 95% CI = [−0.003, 0.006]).

We also investigated the relationship between action learning rate and wellbeing, but found no significant relationship either in the confirmatory sample (linear: *t*(1555) = −0.994, *p* = 0.321, *β* = −0.003, 95% CI = [−0.053, 0.047], quadratic: *t*(1555) = 0.969, *p* = 0.333, Cohen’s ${f}^{2}$ = 0.001, 95% CI (bootstrapped) = [0.000, 0.006])) (Fig. 5d) or after conducting the preregistered meta-analysis across discovery and confirmatory samples (linear: *z* = −1.021, *p* = 0.307, *β* = 0.026, 95% CI = [−0.076, 0.024]; quadratic: *z* = 0.863, Cohen’s ${f}^{2}$ = −0.002, 95% CI = [−0.002, 0.006]).

Finally, we were interested in whether reward learning rate related to wellbeing, but did not find any significant relationship. Wellbeing did not significantly predict reward learning rate either in the confirmatory sample alone (*t*(1556) = 0.651, *p* = 0.515, *β* = 0.016, 95% CI = [−0.033, 0.066]) (Fig. 5e), or after conducting the preregistered meta-analysis (*z* = −0.284, *p* = 0.776, *β* = −0.007, 95% CI = [−0.058, 0.044]).

Sensitivity analyses controlling for number of posts confirmed that all relationships between parameters and wellbeing remained nonsignificant (see Preregistration Deviations in the Supplementary Material and Supplementary Note 4). Specifically, multiple linear regression controlling for number of posts confirmed there was no significant relationship between wellbeing and habit weight (partial linear coefficient for wellbeing: *t*(1554) = −0.307, *p* = 0.759, *β* = −0.039, 95% CI = [−0.011, 0.089], partial quadratic coefficient for wellbeing: *t*(1554) = 0.515, *p* = 0.606, Cohen’s ${f}^{2}$ = 0.000, 95% CI (bootstrapped) = [0.000, 0.004]), partial linear coefficient for number of posts: *t*(1554) = 4.214, *p* < 0.001, *β* = 0.106, 95% CI = [0.057, 0.156]), nor wellbeing and action learning rate (partial linear coefficient for wellbeing: *t*(1554) = −1.280, *p* = 0.201, *β* = −0.001, 95% CI = [−0.057, 0.041], partial quadratic coefficient for wellbeing: *t*(1554) = 1.223, *p* = 0.222, Cohen’s ${f}^{2}$ = 0.001, 95% CI = [0.000, 0.007], partial linear coefficient for number of posts: *t*(1554) = −8.819, *p* < 0.001, *β* = −0.218, 95% CI = [−0.267, −0.170]), nor wellbeing and reward learning rate (partial linear coefficient for wellbeing: *t*(1555) = 0.626, *p* = 0.531, *β* = 0.016, 95% CI = [−0.034, 0.066], partial linear coefficient for number of posts: *t*(1555) = −0.866, *p* = 0.387, *β* = −0.022, 95% CI = [-0.072, 0.028]).

## Discussion

Computational modelling could open new doors to investigate mismatches between evolved human learning processes and social media^13,15–17^. Here, we develop a computational model of real-world social media data, extracting the relative contribution of multiple cognitive processes. We show across a large Twitter (currently X) dataset that a RL process drives posting, consistent with suggestions that low-level, cross-species learning processes underlie social media behaviour^13,21,36^. Further, we show that compared with a pure RL process, as characterised in previous research^21,36^, social media behaviour is better described by a RL-habit hybrid process.

Understanding how multiple behavioural systems contribute to social media behaviour allows us to ask more nuanced questions about social media effects and interventions, such as how specific features shift the balance between RL vs. habitual behaviour, and how best to disrupt unhealthy behavioural patterns. For example, if a user or population’s social media behaviour is more habitual, altering the rewards they receive and engaging with their explicit motivations might be less effective than altering user interface (context) to influence behaviour^18,30^. Many have raised concerns that social media environments disproportionately encourage habitual behaviour while violating the conditions under which habits are beneficial^13,17,26,56^. Thus, habits could represent vulnerabilities in social media environments, both by driving time-consuming social media use which deviates from one’s goals, and on a societal scale, where habitual social media posting could influence social and political discourse^18,19^.

In a context where the definition of habits is the subject of active debate^47,57^, computational modelling helps reveal the specific behavioural tendencies which underlie habitual use. The association of frequent posting with higher habit weight and lower action learning rate align with work associating habits with perseveration and predictability from longer-term past behaviour, respectively. Theoretical research defines model-free RL as a “goal-directed” system, interacting with a value-free habitual system which perseveres with previous behaviour regardless of changes in expected reward^47^. Consistent with this definition, reliance on perseverative posting behaviour over model-free RL relates across users to other converging signatures of habit, such as self-reported habitual social media use and disruption of posting in response to changing contexts^30^. Additionally, predictability in behaviour across time, which can be understood as indexed by a low action learning rate in our model, has been associated both with habitual phone use^53^ and habitual gym attendance^52^.

Finally, there was no evidence for a relationship between posting latency and overall reward learning rate, although we did find a positive relationship between posting latency and negative reward learning rate in the RL-habit model with two learning rates (Supplementary Material and Supplementary Note 3). The positive relationship between posting latency and negative reward learning rate suggests that more frequent (i.e., possibly more habitual) posters are less sensitive to receiving fewer rewards than expected, although they may retain sensitivity to receiving greater reward than expected. This is consistent with a “positivity bias” wherein humans learn more from positive than negative rewards, even as the habitual system takes control of behaviour^44^. However, we note that the best-fitting model across users (RLH1) did not have a positivity bias, therefore evidence for a positivity bias in this dataset is not strong and future work will be needed to establish whether, and if so under what conditions, humans exhibit a positivity bias when learning from social media rewards. Overall, this computational formalisation of different signatures of habitual use (reliance on perseveration over model-free RL, predictability from past behaviour, reward sensitivity) provides precise insights into the ways in which habits manifest in social media posting.

We additionally found that younger people and women showed a higher influence of RL on social media, as indexed by a lower habit weight parameter. Such populations therefore adjust their posting more adaptably in order to maximise social media Likes. This is consistent with research showing that the influence of the habit system increases for older people^58^, and that social rewards are of particular importance to adolescents^59^, as well as a recent study which found that younger people are more sensitive to rewards on social media^36^. Further, women might face unique challenges on social media, such as the way in which their body image is judged^60^, motivating them to exert more effort maximizing symbolic social approval such as via Likes^61,62^. In other words, both biological and social factors could make adolescents and women more likely to devote the effort associated with higher reward sensitivity over habit when posting on social media. These results therefore support the hypothesis that specific cognitive processes—in particular, the relative balance between RL and habitual learning—could partially underlie demographic differences in social media behaviour.

We did not find any significant relationships between computational reward learning parameters and wellbeing. Importantly, our AHI measure of wellbeing correlates highly positively with other measures of positive affect such as in the Positive and Negative Affect Scale (PANAS), but only moderately negatively with measures of negative affect^45^. Computational psychiatry research has predominantly focused on relating negative measures of wellbeing, including psychiatric disorders, to computational reward learning phenotypes, but results have proven difficult to replicate^63^ or generalize^64^. Indeed, a recent study measuring observable statistical signatures of reward learning found that depressed groups were more sensitive to social media rewards, contrasting with previous findings suggesting reduced sensitivity to reward in depression^20^. Due to the paucity of other comparable studies relating wellbeing to computational parameters extracted directly from social media data, it is not possible to interpret whether this null result signifies the lack of a true effect, or insufficient power. Future work which relates our model parameters to both positive and negative wellbeing in clinical and nonclinical groups^65^ could both help extend computational psychiatry to more ecologically valid contexts, as well as elucidating how wellbeing relates to social media behaviour.

Much previous research inferred cognitive processes from statistical signatures in observable data, such as regression coefficients denoting the predictive relationship between Likes received and subsequent latency or content of posts^20,30,38,39^. However, computational modelling has several advantages over this approach. First, statistical signatures can be ambiguous as to the underlying generative process, with different analysis pipelines yielding conflicting results. For example, although there is a broad understanding that in RL, rewards received should predict posting behaviour at a subsequent time-point, it is not clear how exactly to quantify these two variables. “Rewards received” have been variously operationalized as absolute rewards on a previous post^30^, the average number of rewards received across posts on a previous day^20^, or the difference between the mean amount of rewards in the previous seven days and the rewards on the previous day (approximating prediction error rather than reward)^38^. If a dataset satisfies only some but not all of these statistical signatures of RL, it is not clear which effectively represent the hypothesized underlying process of interest. This can be clarified by simulating interpretable computational models^40^. Further, cognitive modelling rigorously parses the cognitive process into precise quantified components, such as positive vs. negative reward learning rates, allowing more specific distinctions between different cognitive phenotypes^55,66^. Finally, generative models can be simulated under different conditions to predict how behaviour changes when novel environments interact with internal processes^67^.

Thus, establishing which generative model best describes empirical data lays the groundwork for novel, principled and quantitative predictions about how new environments such as social media might interact with specific cognitive tendencies^67^. Because such models formalise psychological theory, this method allows us to test different theory-driven, mechanistic hypotheses about social media behaviour, and in return can inform theoretical accounts of social media use^4,13^.

It is important to note, however, that as an initial step modelling multiple cognitive processes in real-world social media behaviour, both our model and dataset are simplifications of a complex process. We document the specific simplifying assumptions of our model in the Supplementary Material and Supplementary Methods. For example, our model considers only two generative processes underlying posting—the habitual system and model-free RL, whereas in reality there are likely to be many more interactive cognitive processes governing posting—for example, model-based RL^13^ and social learning^38^. Further, our model assumes that rewards are delivered on a ratio schedule, such that the amount of Likes received does not depend on posting latency. This is unlikely to be the case in the real world, where it is possible that Likes do depend to some extent on latency (for example, because more frequent posting increases visibility via the algorithm, or because too frequent posting decreases the likelihood that followers will Like every post), and that social media users actively learn a relationship between posting latency and Likes received. Additionally, our model assumes that all “actions” (i.e., posts) are the same, whereas in reality, social media users may consider posts with different content as separate “actions” which each have different reward schedules.

Future work could therefore reintroduce parameters into the model to account for more complexity—for example, (a) more complex cognitive processes, (b) different actions, such as posts with different types of content, and (c) other types of rewards or punishments. Finally, we here related model parameters to individual differences only via cross-sectional correlations. Future research is therefore needed to dissect the causal pathways between these cognitive processes and individual differences of interest, for example, via longitudinal modelling or experimental intervention.

Our model provides the foundation for a large range of future systematic investigation of cognitive processes such as habits in naturalistic behaviour, including how they relate to persistent social media use, individual differences and political and wellbeing outcomes. Indeed, the highly-spread parameter distributions (Table S1, Supplementary material and Supplementary Note 1) indicate substantial individual variation in reward learning processes, which is yet to be explored and explained in future work. While we initially focused on age, gender and wellbeing due to previous literature demonstrating that these variables relate to differences in both reward learning^33,55,68^ and social media use^32,65^, we hope that this proof-of-concept for identifying individual differences in social media use will prompt future researchers to investigate other individual differences as well as interactions.

We adapt the model from the animal literature^21^, and validate it with model and parameter recovery and model falsification^40,49^. Additionally, because our model does not divide latency into days or weeks, as in some previous work^20,38,41^, it does not rely on assumptions about the timescale on which cognitive processes occur. Given that our model can in general characterize how the speed of any action is modulated by any reward, it could be used in future to describe and compare the cognitive processes underlying other actions—for example, passive social media behaviours such as scrolling for newsfeed rewards, or other real-world online or offline behaviours of interest. To facilitate re-use of our model, we have provided openly available code^69^.

In conclusion, answers to pressing questions about how social media is changing life and society are currently held back by a lack of effective scientific methods for measuring and understanding social media use^10^. Here, we have introduced a computational model of real-world behaviour, taking advantage of both decades of cognitive theory and a recent explosion in available digital footprint data. Future work could use this model to compare computational parameters across individuals, algorithms and other conditions, to shed light on policy-relevant problems ranging from online political discourse to the wellbeing impact of social media use.

## Methods

### Dataset

Data collection was approved by the University of Pennsylvania Institutional Review Board (IRB protocol #816091 and associated IRB-confirmed exemptions for publicly available data), which covered our data collection protocol and waived parental consent for all participants, including minors (i.e., those in our dataset aged between 13 and 18). All participants provided informed consent and were not compensated financially for participation.

Our dataset constituted a convenience online sample taking wellbeing surveys through the Authentic Happiness website (https://www.authentichappiness.sas.upenn.edu/) hosted by the University of Pennsylvania. Participants volunteered their Twitter (currently X) handles during sign-up, as well as providing date of birth, age, gender and wellbeing via the Authentic Happiness Inventory (AHI)^45^. The AHI is a 24-item questionnaire, which is a validated measure of current overall happiness. Each item requires participants to rate on a scale of 1–5 how they have been feeling in the last week (e.g., ‘1 = my life does not have any purpose or meaning, 5 = I have a very clear idea about the purpose or meaning of my life; 1 = I am pessimistic about the future, 5 = I feel extraordinarily optimistic about the future)^45^. Scores from the 24 items are summed and averaged to provide an overall happiness score, which has been shown to correlate highly with other measures of positive wellbeing such as in the PANAS^45^.

Using participants’ Twitter (currently X) handles, their timeline of posts (with timestamps) was obtained using the Twitter (currently X) API from January 2023 to February 2023, including Like and Retweet counts of every Twitter (currently X) post JSON object. All downloads and uses of these data complied with Twitter/X’s Terms of Service.

We obtained the maximal number of retroactive posts per user provided by the API. A total of 16,229,780 posts from March 2006 to February 2023 was obtained from a total of *n* = 11,625 users. These data were then further pre-processed as described in “Pre-processing” below, resulting in a final sample of 684,943 posts from *n* = 2696 users (split into a discovery and confirmatory sample, following the study protocol).

### Protocol

To select a subset of the dataset as the “discovery sample”, we first identified how many users had at least 500 posts (*n* = 6923), and then selected a random subset of *n* = 3000 of these users, without performing any other analyses on the rest of the dataset. Pre-processing the discovery sample left a sample of *n* = 1138 (706 self-reported female, 432 self-reported male). We then preregistered our entire analysis pipeline on the Open Science Framework (https://osf.io/jybhx)^46^. Finally, we performed all analyses on the remainder of the dataset (the “confirmatory sample”), which included *n* = 1558 (998 self-reported female, 560 self-reported male) after pre-processing. Given the shortage of previous research using computational modelling of social media data, no statistical method was initially used to predetermine sample size as effect sizes were not known. However, we ensured that the confirmatory sample would be larger than the discovery sample in the hope that any effects with the same effect size should be replicated.

Our analysis pipeline included (a) model validation, and then (b) hypothesis testing. To complete model validation, we verified model and parameter recovery (Supplementary Material and Supplementary Note 2) as well as testing for the presence of statistical signatures of the algorithmic process of interest in both the synthetic and empirical datasets—specifically, a statistical signature of RL and a statistical signature of habits^40,49^ (the statistical signature of RL is shown in Fig. 3b, while the statistical signature of habits is reported in the Supplementary Material and Supplementary Note 2).

Subsequently, to test our hypotheses, we began by establishing the cognitive process underlying posting behaviour across users. We fitted seven computational models to the dataset and established the winning model, representing the inferred underlying process, as that with the highest mean AICw^50^. AICw is a method of single-subject maximum likelihood estimation which, unlike other popular methods such as hierarchical or maximum a posteriori model fitting, incorporates minimal prior assumptions about the value or distribution of parameters. This was appropriate given the paucity of previous studies fitting computational models to social media data, which could have been used to estimate plausible priors.

Next, we were interested in how specific model parameters related to posting frequency, demographics and wellbeing. To investigate this, we extracted best-fitting parameters for each participant and conducted linear regression analyses relating them to posting latency and other linked variables.

### Pre-processing

To pre-process the data, we first removed all Retweets (RTs) from the datasets, as the Twitter (currently X) API did not record the amount of Likes on RT posts, and furthermore, we reasoned that the rewards for RTs may have a different psychological meaning from those for original posts. Next, we grouped all posts that occurred within 300 s of each other into one single “post”, where the amount of Likes and RTs on this post was equal to the maximum number of Likes on a single post in that group, and the “time” at which it was posted was equal to the time at which the first post in that group was posted. This is because we expected posts which happened closely in time not to have yet accrued many Likes, therefore the number of Likes on such posts would likely not have influenced the latency of the subsequent post. Additionally, such posts might have been “thread” posts, which were intended as the same post but separated due to character length. We then restricted each participant’s data to the year-long period starting 6 months before and ending 6 months after they completed the AHI questionnaire and demographics. We restricted the period to one year because learning strategies and, therefore, parameters may change over long time periods, and we wanted a window where parameters would remain relatively constant. We restricted to the year around the AHI completion so that the wellbeing scores would be relatively contemporaneous to the Twitter (currently X) data. These time restrictions yielded a final dataset with post dates ranging from 30/06/2012 to 07/08/2020 in the discovery sample, and from 26/08/2011 to 08/08/2020 in the confirmatory sample.

We then applied the following exclusion criteria. For all analyses, we retained only the participants with at least 80 posts after all the pre-processing steps above. Further, for the analyses which included age as a variable, we additionally excluded all individuals whose age was less than 13 (the legal age to have a Twitter (currently X) account) or greater than 99, on the basis that their age was likely to be inaccurately recorded. Age was calculated as the amount of time between each participant’s self-reported date of birth and the date they completed the AHI. In the discovery sample, after applying the first exclusion criterion, 42 individuals entered their age as below 13, and one as over 99. In the confirmatory sample, 29 people were aged under 13 and one over 99.

### Model fitting procedure

Model fitting was performed in R using the “optim” function to minimize the negative log likelihood of the data. All code for model fitting can be found online^69^.

### Computational models

We fitted seven models to the data: two baseline models, one habit model, two RL models, and two hybrid RL-habit models (Fig. 2). Both the habit and RL models are adapted from previous literature on human and animal reward learning^21,47,70^. The precise derivations of the RL and habit models from studies on animal behaviour are detailed in the Supplementary Material and Supplementary Methods.

The “rewards” in each model are the number of Likes on each post, although sensitivity analyses confirmed that the key model comparison findings are also robust to the operationalization of rewards as RTs, or as the sum of Likes and RTs on each post (Supplementary Material and Supplementary Note 3). Every model specifies a policy, ${{Policy}}_{t}$, which dictates how each posting latency (the time taken from the last post until the current post, at timepoint $t$) is determined. For every model, the posting latency, ${\tau }_{{{Post}}^{t}}$, at time $t$ is determined stochastically by ${{Policy}}_{t}$, as a draw from the exponential distribution with expected value ${{Policy}}_{t}$ (Eq. (1)). However, model comparison findings are not dependent on the specific policy distribution used, with both normal and gamma policy distributions yielding similar results (Supplementary Material and Supplementary Note 3).

1 $${\tau }_{{{Post}}^{t}} \sim {{{\rm{Exponential}}}}\left(\frac{1}{{{Policy}}_{t}}\right)$$

### Baseline models

#### Fixed policy model (FP)

The FP model assumes a constant policy, $P$, which is fit as a free parameter to each individual user’s data.

#### Changing policy model (CP)

In the CP model, the policy evolves across each post according to Eq. (2), where $a$, $b$ and $c$ are free parameters fitted to each user’s data. Thus, this model allows the policy to evolve gradually over time, according to factors that are not modelled in our dataset.

2 $${{Policy}}_{t}=a-b*{e}^{-c*t}$$

### Habit model

#### Habitual policy model (HP)

The HP model is based on a definition of “habit” as a value-free process, following Thorndike’s (1911) Law of Exercise to define habits: that an action that has been taken often in the past is likely to be repeated in the future^47,48^. Unlike RL, which involves value-based learning, habits involve learning from past actions in a form of value-free learning, resulting in perseveration or repetition of previous actions.

The habit model constructs a latent variable, $H$ or “habit tendency”, an estimate of expected future action latency. $H$ is learned using temporal difference learning with “action prediction errors” ${\delta }_{{H}^{t}}$ (Eq. (3)). Specifically, in temporal difference learning, an action prediction error is calculated after each successive action is performed, and is the difference in latency between the action that has actually been taken and the previous habit tendency. Theoretical and experimental research suggests that such a temporal difference learning mechanism underlies habit learning in the brain, with action prediction errors possibly encoded via dopamine^47,71,72^. At each step, $H$ is then updated by combining the previous $H$ with the action prediction error.

The initial $H$ is estimated from the data as the first posting latency in each user’s dataset, but we confirmed that our model comparison results were robust to this approximation by repeating analyses with different initializations (Supplementary Material and Supplementary Note 3). The action learning rate ${\alpha }_{A}$ is a free parameter fitted for each participant, which controls the relative influence on $H$ of more recent actions compared with actions further in the past (Eq. (4)). ${H}_{t}$ is then used as the policy (Eq. (5)).

3 $${\delta }_{{H}^{t}}={\tau }_{{{Post}}^{t}}-{H}_{t}$$

4 $${H}_{t+1}\longleftarrow {H}_{t}+{\alpha }_{A}*{\delta }_{{H}^{t}}$$

5 $${{Policy}}_{t+1}={H}_{t+1}$$

### RL models

#### Single learning rate RL (RL1)

The RL model aims to maximise the average subjective reward rate; that is, the reward, minus costs, obtained per unit time^21,42,43^. To do so, the model estimates the reward expected on the next post, ${\hat{R}}_{t+1}$, using temporal difference learning. The expected reward ${\hat{R}}_{t}$ is first compared with observed reward ${R}_{t}$ to compute the reward prediction error, ${\delta }_{{R}^{t}}$, which, like action prediction errors, is likely encoded via dopamine in the brain^71,73^ (Eq. (6)). This prediction error ${\delta }_{{R}^{t}}$ is then scaled by the reward learning rate ${\alpha }_{R}$ (a recency-weighting mechanism analogous to the action learning rate in the HP model) and added to the previous expected reward to update the expectation, and result in a new expected reward, ${\hat{R}}_{t+1}$ (Eq. (7)). To compute ${{Policy}}_{t+1}$, the expected reward ${\hat{R}}_{t+1}$ is then combined with the subjective vigour cost constant $C$ (Eq. (8)). Specifically, the policy is inversely proportionate to the expected reward, such that higher expected rewards produce shorter posting latencies (Eq. (8)).

The vigour cost constant $C$ associates each posting action with a “vigour cost”, where the vigour cost of action ${\tau }_{{{Post}}^{t}}$ is equal to $\frac{C}{{\tau }_{{{Post}}^{t}}}$, and thus is higher for shorter posting latencies^42,70^. Mathematically, the vigour cost constant represents the rate at which the vigour cost decreases as posting latency increases. If there were no (subjective) costs associated with posting, there would be no reason not to try to post infinitely fast, regardless of reward. However, given that each action (i.e., each post) is associated with a subjective cost, it is only worth increasing posting frequency if the corresponding expected reward from doing so justifies the increased cost.

In the real-world, the “cost” of posting might correspond to any factor which deters an individual from posting fast—such as the increased cognitive effort as well as the opportunity cost of time associated with posting more quickly instead of engaging in other activities. The “vigour cost” of posting specifically denotes any effort and time which is greater for each post at a higher frequency than for each post at a at lower frequency—as the vigour cost per post, $\frac{{{{\rm{C}}}}}{{{{{\rm{\tau }}}}}_{{{{{\rm{Post}}}}}^{{{{\rm{t}}}}}}}$, is greater for smaller ${{{{\rm{\tau }}}}}_{{{{{\rm{Post}}}}}^{{{{\rm{t}}}}}}$. For example, posting very fast on Twitter (currently X) might be more costly relative to posting slowly because posting fast requires one to actively search for new material to post about rather than simply posting whenever news comes up, as well as to post more creatively to ensure less overlap with multiple other recent posts. A figure showing how varying the vigour cost constant $C$ affects the relationship between expected reward and policy is contained in the Supplementary Material and Supplementary Methods.

The initial values for both ${Policy}$ and expected reward $\hat{R}$ were estimated from the data, and we confirmed that our main model comparison results were robust to different initialization decisions by repeating model comparison with both ${Policy}$ and $\hat{R}$ initialized using a variety of different values (Supplementary Material and Supplementary Note 3). In the main analyses, ${Policy}$ is initialized as $C$ divided by the mean value of Likes across all posts for that user, while $\hat{R}$ is initialized as the number of rewards on the first post. The free parameters for each user are the reward learning rate, ${\alpha }_{R}$, and the vigour cost constant $C$.

6 $${\delta }_{{R}^{t}}={R}_{t}-{\hat{R}}_{t}$$

7 $${\hat{R}}_{t+1}\longleftarrow {\hat{R}}_{t}+{\alpha }_{R}*{\delta }_{{R}^{t}}$$

8 $${{Policy}}_{t+1}=\,\frac{C}{{\hat{R}}_{t+1}}$$

#### Double learning rate RL (RL2)

The double learning rate RL model differs from RL1 only in that rather than a single reward learning rate ${\alpha }_{R}$, there are two separate reward learning rates for positive and negative prediction errors; ${{\alpha }_{R}}^{P}$ and ${{\alpha }_{R}}^{N}$, respectively. This allows the model to instantiate a “valence bias” in learning, consistent with research showing that humans typically weight positive and negative rewards differently during learning^44,74,75^. ${{\alpha }_{R}}^{P}$ and ${{\alpha }_{R}}^{N}$ are both fit as free parameters, which can vary across participants, along with vigour cost constant $C$. Which learning rate is used for each reward update is determined according to Eq. (9).

9 $${\alpha }_{R}=\,\left\{\begin{array}{c}{{\alpha }_{R}}^{P}\,{{{\rm{if}}}}{\delta }_{{R}^{t}} \geq 0\\ {{\alpha }_{R}}^{N}\,{{{\rm{if}}}}{\delta }_{{R}^{t}} < 0\end{array}\right.$$

### Hybrid RL-habit models

#### Single learning rate RL-habit (RLH1)

The hybrid models are adapted from a model in the human and animal psychology literature, which characterises behaviour as jointly motivated by a habitual system and a RL system^47^. The habitual component computes the habit tendency $H$ at each timepoint, using Eqs. (3–5), which is translated into the “habit policy” ${{Policy}\left[H\right]}_{t}$. The RL component computes the RL policy, ${{Policy}\left[RL\right]}_{t}$, using Eqs. (6–8).

The two policies are then combined to produce the final policy, with the “habit weight” ${\omega }_{H}$ governing the relative balance between the habitual and RL systems, according to Eq. (10). The initial values for ${Policy}$, habit tendency $H$ and expected reward $\hat{R}$ were estimated from the data as follows. As with the pure RL models, the initial ${Policy}$ was set as the vigour cost constant $C$ divided by the mean received reward. The initial expected reward, $\hat{R},$ was set as the number of Likes on the first post, while the initial $H$ was set as equal to the initial ${Policy}$. Robustness analyses confirmed that model comparison results were robust to varying these initializations (Supplementary Material and Supplementary Note 3). ${\omega }_{H}$ is a free parameter fit to each individual’s data.

10 $${{Policy}}_{t}=\left({\omega }_{H} * {{Policy}[H]}_{t}\right)+(\left(1-{\omega }_{H}\right) * {{Policy}[RL]}_{t})$$

#### Double learning rate RL-habit (RLH2)

RLH2 is the same as the RLH1, with the addition that the RL component has separate learning rates for positive and negative prediction errors, like RL2, and as described in Eq. (9).

### Behavioural signature of reward learning

While model comparison establishes the “predictive performance” of our winning model, we also wanted to verify its “generative performance”: the ability to reproduce a statistical signature of the cognitive process of interest when simulated^40,49^. We therefore identified model-agnostic statistical signatures of reward learning and habits, which we used to validate the generative performance of the models (a) across participants, and (b) between participants.

The statistical signature of reward learning is the predictive relationship between the reward prediction error (RPE) at timepoint $t-1$, which we denote as ${\delta }_{{R}^{t-1}}$, and the difference in posting latency at time *t* compared with time $t-1$, which we denote as $\Delta {\tau }_{{{Post}}^{t}}$. We quantify this relationship using linear regression to extract the coefficient, ${\beta }_{{RPE}}$, denoting how strongly ${\delta }_{{R}^{t-1}}$ predicts $\Delta {\tau }_{{{Post}}^{t}}$. We consider a more negative ${\beta }_{{RPE}}$ to be a behavioural signature of RL because in RL, prediction errors cause an update in expected reward and therefore latent policy at the next step. A more positive RPE increases expected reward, which decreases subsequent policy, producing (probabilistically) a lower posting latency. This behavioural signature of RL is similar to that used in previous work^29,76^.

To compute ${\delta }_{{R}^{t-1}}$, we first estimated the “expected reward”, ${\hat{R}}_{t-1}$, as the mean of the rewards received on the ten preceding posts ($t-2$ to $t-11$). We note that this is an imperfect estimate of reward prediction, as the model’s actual reward prediction is learned through RL and depends on an individual’s reward learning rate. However, the approximate nature of the reward prediction estimate does not affect results: the model falsification result is robust to different ways of estimating reward prediction, both using all preceding posts and using the five preceding posts (Supplementary Material and Supplementary Note 3).

We then obtained the prediction error ${\delta }_{{R}^{t-1}}$ by subtracting the Likes (reward) received at post $t-1$ from this expected reward. To pre-process this RPE variable, we scaled it within each user (subtracting the mean and dividing by the standard deviation). To compute $\Delta {\tau }_{{{Post}}^{t}}$, we first log-transformed ${\tau }_{{{Post}}^{t-1}}$ and ${\tau }_{{{Post}}^{t}}$ (because the distribution was skewed) and then subtracted ${\tau }_{{{Post}}^{t-1}}$ from ${\tau }_{{{Post}}^{t}}$. We then scaled the $\Delta {\tau }_{{{Post}}^{t}}$ within each user (deviating from our preregistered plan, see Preregistration Deviations in the Supplementary Material and Supplementary Note 4). Finally, we used a Multilevel Model (MLM), with users as random slopes, to relate ${\delta }_{{R}^{t-1}}$ to $\Delta {\tau }_{{{Post}}^{t}}$ and extract ${\beta }_{{RPE}}$.

We used the fixed effect of ${\beta }_{{RPE}}$ to evaluate the generative performance across participants, i.e., in model falsification. This is reported in the Main text (Fig. 3b). We then used the random effects of ${\beta }_{{RPE}}$ to evaluate the generative performance of the winning model between participants, i.e., the extent to which the RLH1 habit weight relates to the extent of RL vs. habit control within each individual. This is reported in the Supplementary Material and Supplementary Note 2.

### Behavioural signature of habit

As well as reward learning, we developed a statistical signature of habit learning in which we related the simulated and fitted habit weight across users to autocorrelation across time, a signature of the perseverative nature of habits. Details of this model validation analysis are contained in the Supplementary Material and Supplementary Note 2.

### Statistical inference

All statistical analyses were conducted using *R* statistical software (version 4.2.0)^77^. We preregistered that effects would be considered true if they were significant in the confirmatory sample at the $\alpha$ = 0.05 significance level, or a subsequent fixed-effects meta-analysis across the discovery and confirmatory samples, using the “metafor” package in *R*^78^. This method of statistical inference is consistent with another recent computational modelling registered report^79^.

We also preregistered that we would use one-tailed statistical tests for inference where a directional hypothesis had been stated. However, throughout, we instead report the *p*-value for two-tailed tests, because this is more stringent, except in cases where statistics for the winning model were only significant at one-tailed tests, where the one-tailed test is explicitly stated. Therefore, all statistical tests are two-tailed unless stated otherwise.

The assumptions of statistical tests were assessed by inspection of standard diagnostic plots, including residual Q–Q plots to test for normality. We employed visual inspection rather than formal tests due to the large sample size (*n *= 1558). No substantial deviations from model assumptions were observed.

Statistics throughout the paper are reported for the confirmatory sample unless stated otherwise. To control for effects of number of posts on parameter values, we repeated all individual difference analyses in the main paper (i.e., analyses relating posting latency, age, gender and wellbeing to parameter values) controlling for number of posts. This control deviated from our preregistration; for further explanation, see Preregistration Deviations in the Supplementary Material and Supplementary Note 4.

### Reporting summary

Further information on research design is available in the Nature Portfolio Reporting Summary linked to this article.

## Supplementary information

Supplementary Information

Reporting Summary

Transparent Peer Review file

## Data Availability

The discovery and confirmatory datasets, created after pre-processing, have been deposited on Zenodo and are publicly available online with the following 10.5281/ZENODO.19608894^80^. This dataset was obtained through the Authentic Happiness Website (https://www.authentichappiness.sas.upenn.edu/) and the Twitter (Currently X) API, and then pre-processed according to the protocol described in “Methods”, using analysis code which can be found online (see “Code Availability”).

## Code availability

The code used for all analyses are freely available online, on Zenodo with the following DOI: 10.5281/ZENODO.19608808^69^. They are also available at the GitHub repository: https://github.com/georgiaturner/rl_twitter.git.
