# Supplementary material for: A computational model of reward learning and habits on social media
Source: Nat Commun. 2026 Jun 4;17:7170. doi: 10.1038/s41467-026-73547-6 (PMC13396645; doi:10.1038/s41467-026-73547-6)
Supplement: Supplementary file 1 — Supplementary Information [file 41467_2026_73547_MOESM1_ESM.pdf]

Supplementary Material

Table of Contents

Supplementary Methods *2*

Model derivation 2

i. Baseline models 2

ii. Reinforcement learning models 2

iii. Habit models 8

Supplementary Note 1 *9*

Descriptives 9

i. Fitted parameter values 9

Supplementary Note 2 *11*

Model validation 11

i. Model and parameter recovery 11

ii. Statistical signature of reward learning 17

iii. Statistical signature of habit 20

Supplementary Note 3 *24*

Sensitivity analyses 24

i. Model comparison with different definitions of reward 24

ii. Model comparison with different policy distributions 26

iii. Model comparison with different initializations 28

iv. Model comparison with the RL model of Lindström et al. (2021) 30

v. Model falsification with different definitions of reward prediction error 33

vi. Results from the RLH2 model 34

vii. Posting latency correlations with outliers removed 40

Supplementary Note 4 *42*

Preregistration deviations 42

References *44*

Supplementary Methods

Model derivation

This section describes the precise mathematical and conceptual derivations by which we adapted our models from the human and animal psychology literature.

i. Baseline models

The Fixed Policy (FP) model, assuming a constant posting latency, is equivalent to the 'no learning' baseline model originally used by Lindström et al. (2021)[1]. Given that this model makes the stronger assumption of no *change* as well as no learning, we here supplemented it with another baseline 'no learning' model, the Changing Policy (CP) model, which allows for change across time, and thus provides a stronger control for the cognitive models.

ii. Reinforcement learning models

Our RL model is inspired by the insight of Lindström et al. (2021)[1] that the speed of posting on social media might be describable by the same cognitive process as an animal model of rats obtaining rewards in psychological experiments[2,3]. However, we did not employ Lindström et al.'s original model for two reasons. First, their RL model induced a spurious autocorrelation between posts, because the expected reward was mathematically dependent on the size of the previous posting latency (because of the appearance of the posting latency term in their Equation 2, which constructs the expected reward[1]). Therefore, a longer posting latency would tend to lower the estimated reward for the next post, which in turn would tend to increase posting latency for the next post, and so on. As such, this model could provide a good description of behaviour not only because of the RL component, but alternatively because of

autocorrelation across posting latencies. We instead wanted an RL model which would capture a purely reward-driven process, such that the estimate of expected reward was not influenced by previous posting latency. This could then be compared to hybrid models which explicitly added the autocorrelaton component on top (i.e., our RLH1 and RLH2 models), thus dissecting the relative contributions of each of these components. Second, Lindström et al.'s model was complex, therefore to enhance interpretability, we wanted a simpler model wherein the influence of each parameter on observable behaviour would be clear and intuitive.

We therefore returned to the original model of animal behaviour, by Niv et al. (2005, 2007)[2,3], to derive our RL model. The definition of average (subjective) reward rate our RL model uses can be mathematically derived from the original model by Niv et al., given several assumptions. We make these assumptions both to adapt our model from the general case detailed in Niv et al. to the specific social media context, and to simplify the model to enhance interpretability. We discuss and justify each of these assumptions below.

Niv et al.[2,3] provide an equation to deduce the optimal average reward rate and the associated optimal latency between actions, where 'average reward rate' describes the maximum rewards that can be obtained, minus the costs, per unit time. We first assumed that reward is delivered on a ratio schedule, i.e. that the amount of Likes received on each post is not influenced by posting latency. This simplifying assumption may not be satisfied in the case of real Twitter posting, where posting latency could plausibly affect Likes on posts, for example via algorithms and resulting increased follower numbers, and people may instrumentally adjust their posting latency to influence this. However, the assumption of a ratio schedule allows the optimal posting latency for a given optimal reward learning rate to be solved analytically[2]. Importantly, the inverse relationship between expected reward and optimal posting latency still holds for interval schedules, therefore the 'ratio schedule' assumption is unlikely to greatly affect qualitative results.

Given a ratio schedule, the animal model in Niv et al.[2] states that the optimal posting latency, $\hat{\tau}$, for action a_i is given by Equation S1, such that $\hat{\tau}$ depends on the vigour cost constant of completing action a_i after a_i, $K_v(a_i,\ a_i)$, and the estimated average reward rate $\bar{r}$. $K_v(a_i,\ a_i)$, the 'vigour cost constant', determines the the latency-dependent subjective 'vigour cost', which is proportional to $\frac{K_v(a_i, a_i),}{\tau_{Post}t}$, of performing action a_i after a_i. This cost is higher for shorter latencies[4]. In the real world, the vigour cost might represent an 'effort cost', or more generally a cost resulting from any variable which decreases the subjective value of behaving more quickly – for example, in the social media context, (perceived) reputational cost.

(Equation S1) $$\hat{\tau}_{a_i} = \sqrt{\frac{K_v(a_i, a_i)}{\bar{r}}}$$

The average reward rate $\bar{r}$ is given by Equation S2, also taken from Niv et al.[2].

(Equation S2) $$\bar{r} = \left(\frac{U(r)+n\rho(a_i)+\rho(a_j)}{2\left[(n-1)\sqrt{K_v(a_i, a_i)}+\sqrt{K_v(a_i, a_j)}+\sqrt{K_v(a_j, a_j)}\right]}\right)^2$$

In Equation S2, we see that $\bar{r}$ depends on $U(r)$, the immediate reward r for the next action, scaled by a subjective utility function $U(\,)$. n represents the amount of times action a_i needs to be repeated to obtain this immediate reward.

a_i and a_j are two different possible actions needed to obtain a reward. The vigour cost constant $\sqrt{K_v(a_{prev},\ a)}$ can vary depending on the preceding and subsequent action: here, $\sqrt{K_v(a_{prev},\ a)}$ denotes the specific vigour cost of carrying out action a after the previous action a_{prev}.

$\rho(a_i)$ is the intrinsic or internal reward associated with action a_i (i.e., any further subjective reward not modelled by the external reward $U(r)$). In the social media case, an example of such intrinsic reward for posting might be satisfaction resulting from self-disclosure[5].

To simplify these equations, we made the following assumptions:

(Equation S3) $U(r) = r$

(Equation S4) $\forall \, (a_i = a_j)$

(Equation S5) $\rho(a_i) = 0$

(Equation S6) $n = 1$

Equation S3 states that all Likes are of equal subjective value. Previous work suggests that this assumption may not always be satisfied, with Likes sometimes displaying diminishing marginal utility[1], and future investigation into how subjective reward utility differs across users might wish to reintroduce this parameter to the model.

Equation S4 states that all actions are equivalent (i.e., each action is a single post), though future work may wish to relax this assumption, for example defining posts with different types of content as different 'actions'. Therefore $K_v(a_i, a_i)$ simplifies to K_v.

Equation S5 states that there is no intrinsic reward for action a_i, so that the only 'reward' obtained for each post is the external Likes. In the case of social media, there may well be intrinsic rewards which motivate behaviour, for example satisfaction or identity development from self-disclosure, which could again be reintegrated in future work with this parameter.

Finally, Equation S6 states that the number of actions (in the social media scenario, posts) necessary to obtain a reward is 1. This is more suited to the way in which Likes function on social media, with each post potentially receiving Likes independently.

With the assumptions in Equations S3-S6, Equation S2 simplifies to Equation S7.

(Equation S7) $$\bar{r} = \left(\frac{r}{2*2\sqrt{K_v}}\right)^2 = \frac{r^2}{16K_v}$$

We can then substitute Equation S7 back into Equation S1, to obtain a new, simpler equation for the optimal posting latency, Equation S8.

(Equation S8) $$\hat{t} = \sqrt{\frac{K_v}{\frac{r^2}{16K_v}}} = \frac{4K_v}{r} = \frac{C}{r}$$

Equation S8 shows that the optimal posting latency is inversely proportionate to the expected immediate reward r, scaled by a constant, C (where $C = 4K_v$). Thus, our final RL model learns a policy as this optimal posting latency (i.e., Equation S8 is equivalent to Equation 8 in the main paper), where C is a free parameter held constant for each user.

Specifically, C represents the vigour cost constant, which determines the vigour cost of each action, $\frac{C}{\tau_{Post}t}$, with the vigour cost increasing for actions with shorter latencies. In the real world, the vigour cost might represent any subjective cost which is greater for higher frequency posting, such as the increased effort required to actively come up with more varied and creative posts rather than simply wait until a good idea for a post comes along, as well as the opportunity cost of not being able to engage in other activites. As shown in Figure S1, for a given expected reward per post, a higher vigour cost constant will result in a higher policy (i.e., a slower posting latency). This is because a higher vigour cost constant translates to a higher subjective cost of posting, which decreases the subjective reward, and results in a policy to post at longer latencies (less often).

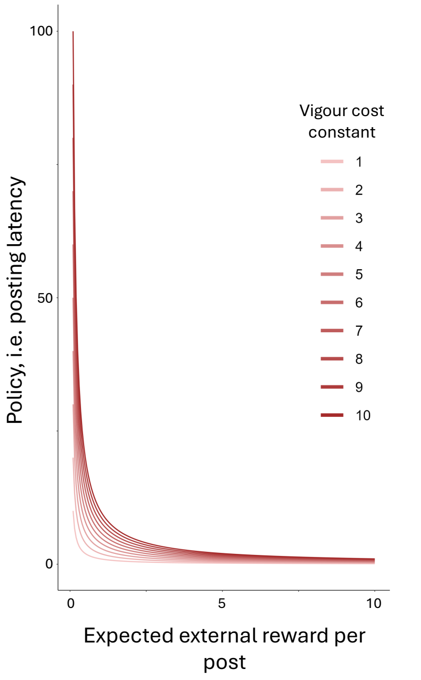

Figure S1. The relationship between expected reward and policy in the RL model depends on the vigour cost constant. For a higher vigour cost constant, the policy will be higher for a given expected reward.

Because Niv et al.[2] did not include learning, we added learning to the model, such that the expected immediate reward r is computed by temporal difference learning (Equations 6-7, main paper), before being substituted into Equation 8.

A strength of our model, in contrast to much previous work investigating RL on social media[6–8] (although consistent with Lindström et al. (2021)[1]), is that posting latency is modelled as a continuous variable rather than quantized in time periods (e.g. days[6,9] or weeks[7]). Avoiding assumptions about the timescale on which cognitive processes occur has at least two advantages. First, this model is powerful enough to identify processes occurring on different timescales (e.g. two days, three days) which would have been simply interpreted as lack of evidence for the cognitive process in models which assume specific timescales a priori. Secondly, this facilitates simple extension of our model to other behaviours wherein habits occur on different timescales. Future work may also consider relaxing the assumptions discussed in Equations S3-S6 (for

example, re-introducing intrinsic reward or subjective utility of external reward) in order to examine further, more granular aspects of the learning process.

iii. Habit models

Our habit models are conceptually inspired by the computational model of animal habits put forward by Miller et al (2019)[10]. Specifically, habits are conceived of as a value-free process which takes the form of perseveration, aligning with Thorndike's (1911)[11] Law of Exercise: that an action that has been taken often in the past is likely to be repeated in the future.

This theoretical definition is used in much traditional classical psychology research, which suggests that, although habits are initially formed when repeating rewarded actions, they represent stimulus-response associations[12,13]. Stimulus-response associations contrast from response-outcome associations[14], as in both model-free and model-based RL. Thus, habits are a form of learning, but the learning pertains to stimulus-response (value-free) rather than response-outcome (value-based) associations[10,15].

This definition of habit as a value-free process differs from a previously proposed view in the computational RL literature, which mapped habits onto model-free RL, and goal-directed behaviour onto model-based RL[16–18]. However, we believe that this previous definition is in contrast with both neural and behavioural evidence supporting the value-free habit definition we use. Specifically, neural studies find no clear separation between neural substrates of model-free and model-based RL[19], contrasting with consistent findings that habitual and goal-directed systems are underpinned by distinct brain regions[20]. Additionally, habitual social media posting, operationalized both through self-report and posting frequency, is associated with perseveration and

reduced reliance on model-free RL – which is not what we might expect if habits were underpinned by model-free RL[21]. Together, this evidence converges towards a view of all reinforcement learning as part of a 'goal-directed', value-maximising system, distinct from a perseverative habitual system.

In our RL-habit hybrid models, therefore, a habitual policy, constituting a recency-weighted average of past actions, is learned through temporal difference learning. As in Miller et al (2019)[10], in the RL-habit hybrid models, this habitual policy is combined with the RL policy, to produce a final policy as a weighted average of the two systems.

Supplementary Note 1

Descriptives

i. Fitted parameter values

Table S1 contains information about the fitted parameter values for all models in both the discovery and confirmatory samples.

Model	Parameter	Sample	Minimum	Maximum	Mean	Median	Standard deviation
Fixed Policy (FP)	Policy	Discovery	0.028	4.589	1.994	1.830	1.157
		Confirmatory	0.065	4.605	1.961	1.760	1.120
Changing Policy (CP)	a	Discovery	-67.059	2245.55	107.031	48.297	179.270
		Confirmatory	-53.008	4856.995	133.187	53.538	281.105
	b	Discovery	-42335.830	2245.540	52.182	48.301	1419.545
		Confirmatory	-12562.360	4855.874	121.6841	52.453	435.103
	c	Discovery	-0.119	5.285	0.067	1.28×10^{-5}	0.396
		Confirmatory	-0.106	4.505	0.041	1.20×10^{-5}	0.264
Habitual Policy (HP)	Action learning rate	Discovery	4.99×10^{-9}	0.841	0.107	0.092	0.075
		Confirmatory	5.18×10^{-9}	0.711	0.114	0.098	0.083
Single learning	Reward learning rate	Discovery	1.21×10^{-15}	0.941	0.040	0.013	0.071

rate RL (RL1)		Confirmatory	8.82×10^{-16}	0.993	0.044	0.014	0.087
	Vigour cost constant	Discovery	2.76×10^{-6}	14656.469	7.495	1.482	60.552
		Confirmatory	1.70×10^{-5}	828.588	4.511	1.432	26.088
Double learning rate RL (RL2)	Positive reward learning rate	Discovery	1.66×10^{-14}	1	0.188	0.006	0.326
		Confirmatory	1.43×10^{-14}	1	0.185	0.006	0.329
	Negative reward learning rate	Discovery	2.32×10^{-11}	0.992	0.060	0.019	0.125
		Confirmatory	9.93×10^{-13}	1.00	0.061	0.019	0.119
	Vigour cost constant	Discovery	2.76×10^{-6}	4981.01	11.640	1.579	153.594
		Confirmatory	2.70×10^{-5}	672.491	5.327	1.590	22.476
Single learning rate RL-habit (RLH1)	Reward learning rate	Discovery	3.042×10^{-14}	1	0.223	0.035	0.325
		Confirmatory	2.244×10^{-14}	1	0.227	0.032	0.330
	Vigour cost constant	Discovery	1.848×10^{-10}	2243.037	9.549	1.575	85.707
		Confirmatory	8.957×10^{-10}	886.862	4.680	1.424	24.218
	Action learning rate	Discovery	2.684×10^{-10}	1	0.228	0.127	0.266
		Confirmatory	7.023×10^{-11}	1	0.242	0.136	0.272
	Habit weight	Discovery	1.281×10^{-09}	1	0.767	0.923	0.287
		Confirmatory	2.248×10^{-10}	1	0.767	0.944	0.295
Double learning rate RL-habit (RLH2)	Positive reward learning rate	Discovery	1.023×10^{-12}	1	0.262	0.015	0.361
		Confirmatory	7.510×10^{-14}	1	0.261	0.016	0.360
	Negative reward learning rate	Discovery	1.586×10^{-10}	1	0.233	0.109	0.281
		Confirmatory	9.450×10^{-11}	1	0.222	0.010	0.274
	Vigour cost constant	Discovery	5.057×10^{-5}	277.576	3.634	1.345	9.237
		Confirmatory	2.868×10^{-9}	105.552	3.731	1.407	7.123
	Action learning rate	Discovery	7.039×10^{-12}	1	0.234	0.125	0.276
		Confirmatory	6.986×10^{-11}	1	0.256	0.148	0.282
	Habit weight	Discovery	1.536×10^{-10}	1	0.762	0.960	0.304
		Confirmatory	1.633×10^{-8}	1	0.760	0.976	0.313

Table S1. List of parameter values for each model in the discovery and confirmatory datasets.

Supplementary Note 2

Model validation

i. Model and parameter recovery

To establish the power of our modelling procedure to uncover the true underlying generative process, we performed model and parameter recovery analyses[22]. These analyses involve simulating the models to create a synthetic dataset, then fitting the same models back to the synthetic datasets. Recovery then assesses the extent to which the model fitting procedure correctly 'recovers' the true underlying simulated models and parameters, and whether the procedure induces spurious biases towards any particular model or parameter values.

To maximally reflect the confirmatory empirical dataset in sample size and range of parameter values, we simulated datasets for each model with 1,500 synthetic participants. We note that these simulated datasets, unlike the datasets we simulated for model falsification, did not use the exact parameters from the social media users. Instead, to ensure that the simulated parameters were not correlated, the simulated parameters for each synthetic participant for these analyses were drawn from normal distributions based on the distribution of parameters in the discovery sample. Specifically, parameters for each model were drawn from normal distributions with means as the mean of the fitted parameters for that model across the discovery sample, and standard deviations as half the interquartile range of the fitted parameters across the discovery sample. We then applied further constraints to simulated parameters reflecting their theoretical constraints in the models. Specifically, a maximum of 1 was applied to all learning rate and habit weight parameters, and a minimum of 0.001 was applied to the learning rates, habit weight and cost parameters.

The simulated 'rewards' or Likes were drawn from a Poisson distribution, where the mean of the Poisson distribution (the parameter λ) evolved according to a Gaussian

walk across posts (see example in Figure 2b of the main paper). To reflect a similar range to the empirical dataset, the starting λ for the Poisson distribution was the median of the mean Likes for each user, while the standard deviation of the Gaussian walk was the median of the range of Likes for each user, divided by 1000 (because the synthetic dataset had 1000 posts).

For both model and parameter recovery, we first performed recovery for 1000 posts to examine results at convergence, i.e. with sufficient data. This allowed us to verify how the models performed when not limited by the data, which will be informative for any future research which re-uses these models on datasets of different sizes. Next, we performed recovery only on the first 80 posts from the simulated dataset. This is because 80 was the minimum number of posts per user in our empirical analyses. We chose 80 because, unlike in traditional experimental settings, given that we were using real-world data we could not control the amount of data available. 80 posts was a compromise between including as many users as possible and having enough posts for satisfactory model recovery. Given that we expected model and parameter recovery to be worse on the n = 80 datasets, we used these analyses primarily to establish whether our modelling procedure favoured the winning models and parameter values, i.e. to assess the likelihood of false positives.

Model recovery

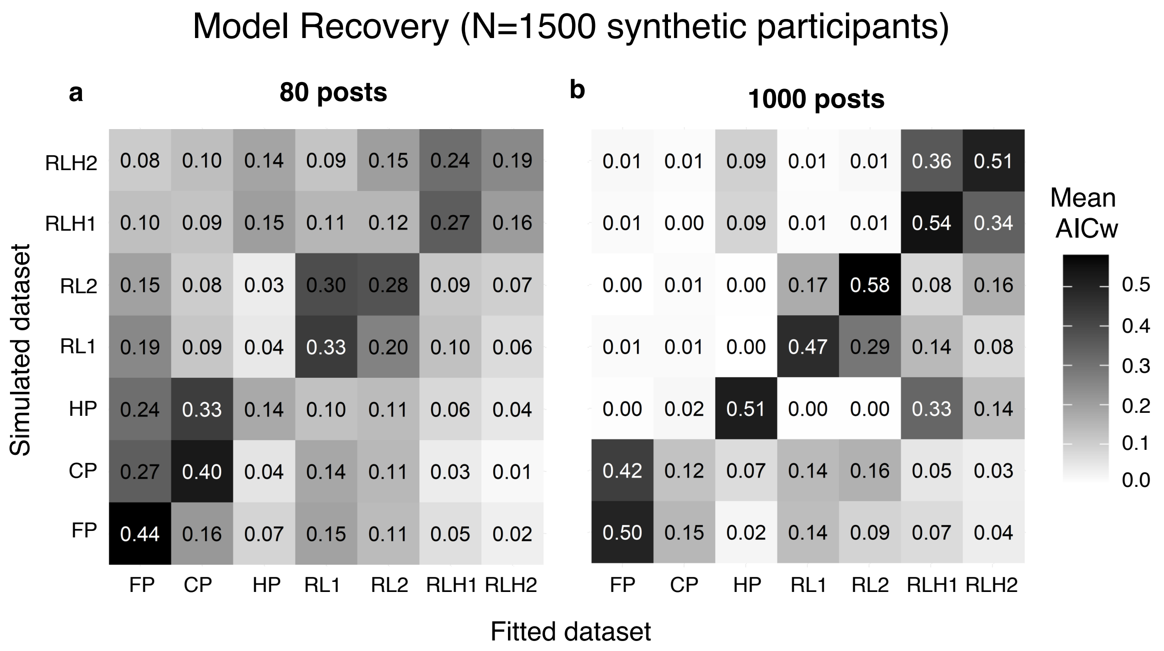

Figure S2. Model recovery. FP = Fixed Policy, CP = Changing Policy, HP = Habitual Policy, RL1 = Pure RL, single learning rate, RL2 = Pure RL, double learning rate, RLH1 = Hybrid RL-habit, single learning rate, RLH2 = Hybrid RL-habit, double learning rate. Shaded colours represent mean AICw for each fitted model across each simulated dataset. Better model recovery is indicated by high mean AICw for the same model fitted to the simulated dataset (i.e., across the diagonal). **a)** Model recovery for 80 posts shows that while the correct model is not always recovered, there is a bias towards the simpler models (those with fewer parameters). Therefore, there is no bias towards the RL-habit models which won in the empirical dataset, reducing the likelihood of false positives and making our model fitting procedure a conservative test of the RL-habit models. **b)** Model recovery for 1000 posts shows excellent recovery. High mean AICws outside of the diagonal are primarily for models which share components – for example, the HP model is sometimes recovered as the RLH1 model, which makes sense as the HP model is a component contained within the RLH1 model; indeed the RLH1 model reduces to HP when habit weight is 1. There is also some confusion between the RL1 and RL2 models, and RLH1 and RLH2 models, which can be attributed to their very similar structure.

We first performed recovery for 1000 posts (Figure S2b). Encouragingly, this model recovery analysis reveals higher mean AICw values in the diagonals than at any other location in the matrix. This means that for each of the seven models, if that model represents the true generative process in a dataset, then that model will also be the

'best fitting' model according to our model fitting procedure (higher mean AICw for that fitting model than for any other model in each row).

However, we also note that model recovery is not perfect. For example, the RLH1 column (Figure S2b) indicates that with 1000 posts, when RLH1 is the winning fitted model, model comparison yields a fairly high possibility that the HP model generated the underlying dataset. However, in this case, model simulation and falsification can be used to test whether the statistical signature of RL is present in a given dataset. If it is, the HP model, which includes no RL component, can be absolutely rejected, regardless of the relative evidence for HP in model comparison. This thus underscores the importance of combining model comparison, a measure of the evidence for each model relative to the set of models considered, with model falsification, an absolute criterion for model rejection[23]. Further, we also note that in Figure S2b, the CP model is poorly recovered and is often incorrectly recovered as the FP. This is a result of the parameters that the CP model was simulated with, which resulted in a mostly consistent policy across time. As the FP and CP models were baseline models, good model recovery between these two is not important for our research questions.

We then considered model recovery for 80 posts, which was the minimum amount of posts for a user to be included in our empirical analyses (Figure S2a). As expected, given fewer data points, model recovery was worse for 80 than for 1000 posts. In particular, model recovery overwhelmingly favoured the simpler models, which is as expected given that AICw, our criterion for model selection, penalizes models with more parameters[24]. This suggests that our modelling procedure was a conservative test for the most complex models RLH1 and RLH2. Given that these were winning models in our empirical analyses, this shows that our model comparison results are unlikely to contain false positives. We chose to use 80 posts as the minimum number of data points per user, both because this model recovery analysis showed a low likelihood of false positives at 80 posts, and because of other considerations specific

to our dataset such as the requirement not to exclude too many users due to a small amount of posts.

We therefore suggest, from this initial validation, that 80 posts function as an initial rubric for minimum number of posts in any similar future research. However, we ideally recommend that future researchers employing our modelling procedure use a process specific to their dataset and research question to select inclusion criteria. First, model recovery with a selected number of posts should be run with parameter and reward distributions reflective of those in the specific dataset under consideration, as these variables in a dataset can have large effects on model and parameter recovery[22]. For example, the ability to recover models with a double learning rate depends on the distribution of the learning rate parameters: it will be easier to distinguish models with double from single learning rates in model recovery if the learning rates in the double learning rate model have a larger difference from each other. Secondly, other considerations such as minimizing number of participants excluded may contribute to lowering or raising the exclusion criterion for number of posts. Third, the specific research question of interest should be considered. We were most interested in the underlying generative process for our data, so model recovery analysis was important in considering number of posts. However, for example, if a researcher is more interested in how a specific parameter value changes across populations or conditions, more weight should be given to the results of a parameter recovery analysis for this parameter than model recovery in determining minimum number of posts.

Parameter recovery

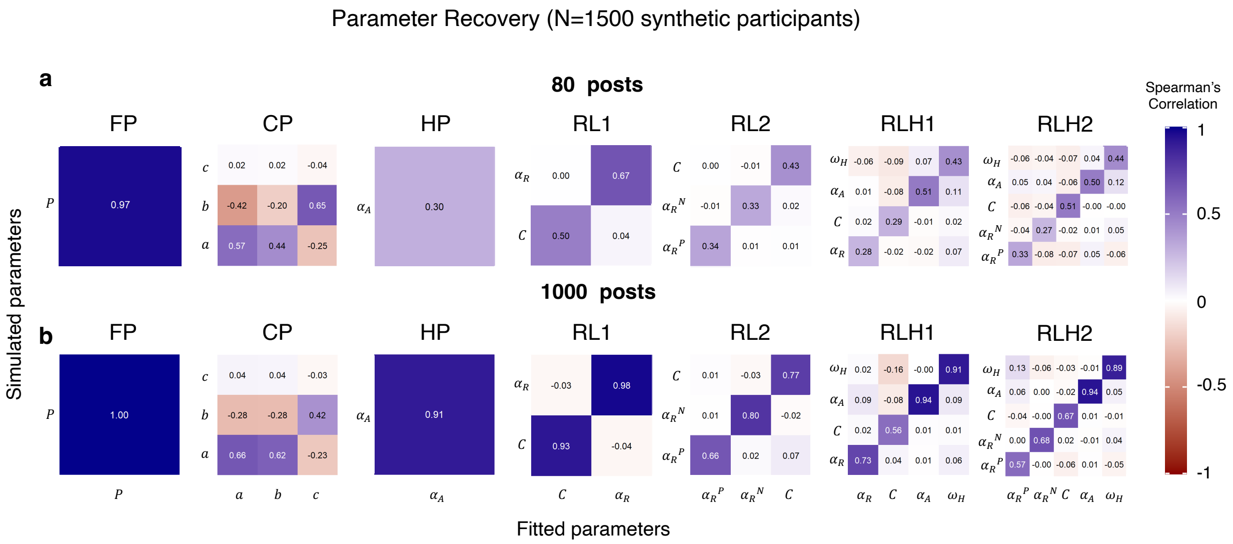

Figure S3. Parameter recovery. FP = Fixed Policy, CP = Changing Policy, HP = Habitual Policy, RL1 = Pure RL, single learning rate, RL2 = Pure RL, double learning rate, RLH1 = Hybrid RL-habit, single learning rate, RLH2 = Hybrid RL-habit, double learning rate. Shaded colours represent the Spearman's correlation between the fitted and actual parameter, for each of the seven models. High Spearman's correlation indicates good parameter recovery. Low correlations for all nondiagonal squares also indicate that the modelling procedure can effectively distinguish between different parameters within the same model, and thus that each parameter represents a distinct aspect of the cognitive process. FP = Fixed Policy, CP = Changing Policy, HP = Habitual Policy, RL1 = Pure RL, single learning rate, RL2 = Pure RL, double learning rate, RLH1 = Hybrid RL-habit, single learning rate, RLH2 = Hybrid RL-habit, double learning rate. **a)** Parameter recovery for 80 posts. **b)** Parameter recovery for 1000 posts indicates very good recovery for all models apart from the CP model, for which we were not interested in exact parameter values.

Mirroring our procedure for model recovery, we then performed parameter recovery for both 80 (Figure S3a) and 1000 (Figure S3b) posts. Specifically, we calculated the Spearman's rank correlation between simulated and fitted parameters for each model. As with model recovery, parameter recovery was very good for 1000, and less strong but still showed correlations in the correct direction for 80 posts. The only model with poor parameter recovery was the CP model, which is likely because the complex

three-parameter curve was often too complex for the trend in the data, meaning there was not a unique solution to the curve. However as we were never interested in parameter values for this model, this is not a problem for our results.

ii. Statistical signature of reward learning

Evaluating model generative performance across users

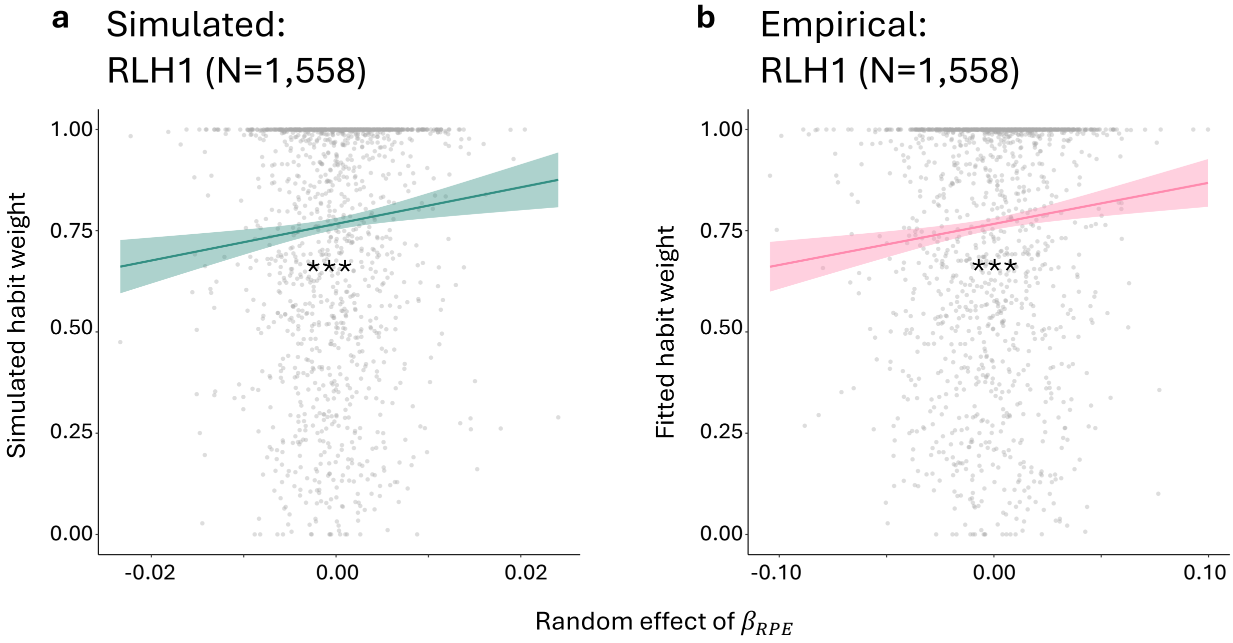

Figure S4. Using the signature of reward learning, β_{RPE}, to verify model generative performance between participants. Scatter graphs show the random effects of β_{RPE} for each user predicting simulated or fitted habit weight. Lines of best fit are shown, with shaded regions representing 95% confidence intervals. Annotations indicate significance, according to the following scheme: ns = not significant, * = $p < 0.05$, ** = $p < 0.01$, *** = $p < 0.001$. **a)** The random effect of β_{RPE} in the RLH1 simulated dataset, simulated with the parameters of the confirmatory sample, positively predicts the simulated habit weight across users ($t(1556) = 3.23$, $p = 0.001$, $\beta = 0.082$, 95% CI = [0.032, 0.131]). **b)** The random effect of β_{RPE} in the confirmatory sample positively predicts fitted RLH1 habit weight ($t(1556) = 3.478$, $p < 0.001$, $\beta = 0.088$, 95% CI = [0.038, 0.137]).

We defined a reward learning signature as the coefficient, β_{RPE}, relating prediction error at timepoint t to the change in posting latency between posts *at* timepoints t and $t + 1$.The procedure for calculating β_{RPE} is reported in the main Methods. To evaluate the generative performance of the winning model between participants, we considered the 'habit weight' parameter, which quantifies the relative weighting of the RL component in the hybrid RL-habit models. We expected participants with a higher habit weight to exhibit the reward learning signature less strongly. We therefore extracted the random effect of β_{RPE} for each individual user, and examined how this reward learning signature predicted the value of the simulated habit weight parameter in the generated RLH1 dataset (the winning model in the AH confirmatory and dataset) (Figure S4a), and the fitted habit weight parameter in the empirical dataset (Figure S4b). β_{RPE} significantly predicted simulated habit weight in the simulated RLH1 dataset (t(1556) = 3.23, p = 0.001, β = 0.082, 95% CI = [0.032, 0.131]) and fitted habit weight in the empirical confirmatory sample (t(1556) = 3.478, p < 0.001, β = 0.088, 95% CI = [0.038, 0.137]). This confirms that the fitted habit weight value does relate to the extent to which individuals exhibit reward learning.

Relating RL signature to posting latency and age across users

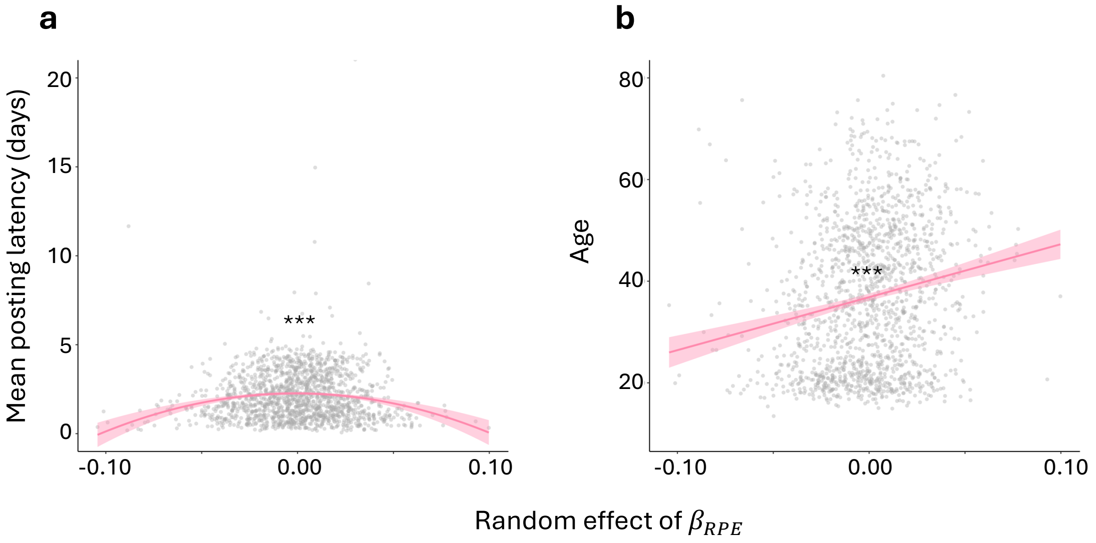

Figure S5. Using β_{RPE} to predict posting latency and age. Scatter plots show the random effect of β_{RPE} across users, predicting posting latency and age. Lines are lines of best fit, shaded areas represent 95% confidence intervals. Annotations indicate significance, according to the following scheme: ns = not significant, * = p < 0.05, ** = p < 0.01, *** = p < 0.001. **a)** β_{RPE} had a negative quadratic relationship with posting latency in the confirmatory sample (t(1555) = -6.667, p < 0.001, Cohen's f^2 = 0.029, 95% CI (bootstrapped) = [0.009, 0.055]). **b)** β_{RPE} positively predicted age in the confirmatory sample (t(1556) = 6.612, p < 0.001, β = 0.165, 95% CI = [0.116, 0.214]).

In our discovery sample, we found two exploratory relationships between β_{RPE} and individual differences in a) posting latency and b) age. To establish whether these were true effects, we preregistered both relationships for testing on the confirmatory sample. As predicted, in the confirmatory sample, β_{RPE} had a significant negative quadratic relationship to posting latency (t(1555) = -6.667, p < 0.001, Cohen's f^2 = 0.029, 95% CI (bootstrapped) = [0.009, 0.055]) (Figure S5a) and a positive linear relationship to age (t(1556) = 6.612, p < 0.001, β = 0.165, 95% CI = [0.116, 0.214]) (Figure S5b).

The relationship with age is in line with the positive relationship between age and habit weight, providing converging evidence that younger people are more reward-sensitive. However, the quadratic relationship with posting latency is less straightforward. One possibility is that there is another process causing frequent posters to also have a positive relationship between RPE and change in posting latency (i.e., the opposite direction from that predicted by the RL model). For example, the dataset does not have the exact timestamps for receiving rewards, therefore a very successful post leads to a delay in posting again rather than speeding up as people continue to receive rewards for a long time. Alternatively, there could be an instrumental process where people speed up posting when they are getting fewer rewards as they expect to get more attention this way – as a simplification of our model was that we assumed a ratio rather than interval reward schedule, which does not account for instrumental changes to posting latency (see 'Model Derivation', above). A further possibility is that habitual social media use is not always more frequent, but instead arises at a specific posting latency, such that both higher and lower posting latencies are less habitual. This ambiguity underscores the dissociation between underlying generative processes and statistical signatures, highlighting the need for mechanistic generative models to adjudicate between different generative processes which could produce similar statistical signatures.

iii. Statistical signature of habit

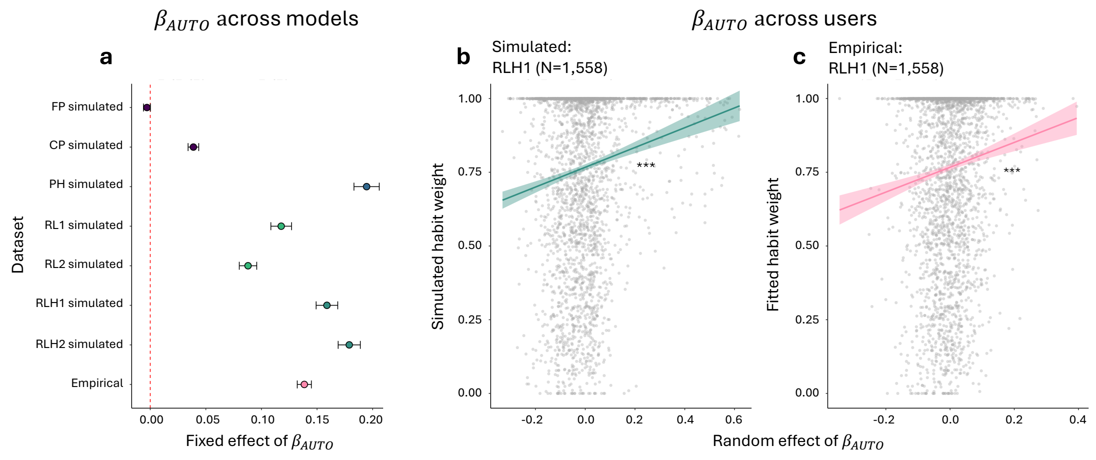

Figure S6. The statistical signature of habit, β_{AUTO}, across models and users. a) Dots show estimates of β_{AUTO}, error bars show 95% confidence intervals. The dataset simulated using the Habitual Policy model has the strongest fixed effect of β_{AUTO}, followed by the two hybrid RL-habit models, RLH1 and RLH2. The pure RL models have smaller fixed effects of β_{AUTO}, but are not 0 due to the approximated nature of the reward prediction being controlled for. **b)** Scatter graphs show the random effects of β_{AUTO} for each user predicting simulated or fitted habit weight. Lines of best fit are shown, with shaded regions representing 95% confidence intervals. Annotations indicate significance, according to the following scheme: ns = not significant, * = $p < 0.05$, ** = $p < 0.01$, *** = $p < 0.001$. The random effect of β_{AUTO} in the RLH1 simulated dataset, simulated with the parameters of the confirmatory sample, positively predicts the simulated RLH1 habit weight across users ($t(1556) = 6.166$, $p < 0.001$, $\beta = 0.154$, 95% CI = [0.105, 0.204]). **c)** The random effect of β_{AUTO} in the confirmatory sample positively predicts fitted RLH1 habit weight ($t(1556) = 4.872$, $p < 0.001$, $\beta = 0.122$, 95% CI = [0.073, 0.172]).

To verify that the habit component of the RLH1 model was also working mechanistically as expected, we developed a statistical signature of habitual behaviour. Specifically, habit is distinguished by the tendency to repeat previous actions regardless of predicted reward. In contrast, in pure RL models RL1 and RL2, posting latency is fully determined by expected reward, therefore there should be no autocorrelation across successive posts after controlling for expected reward.

To quantify this statistical signature of habit, we used multiple linear regression to extract the coefficient β_{AUTO} denoting the extent to which posting latencies are autocorrelated across time over and above expected reward. Specifically, β_{AUTO} represents the predictive relationship between the posting latency at timepoint $\mathrm{t}-1$, which we denote as $\tau_{\mathrm{Post}^{\mathrm{t-1}}}$, and the posting latency at timepoint t, $\tau_{\mathrm{Post}^{\mathrm{t}}}$, controlling for the predicted reward at timepoint $\mathrm{t}-1$, $\widehat{\mathrm{R}}_{\mathrm{t}-1}$. Because the extent to which successive posts are correlated across time over and above expected reward is a signature of habit, within the dataset simulated by the RLH1 model, we expected users with a higher habit weight to exhibit the habit signature more strongly, as indexed by a higher random effect of β_{AUTO}. Additionally, we also expected the fitted habit weight in the empirical dataset to be positively related to the random effect of β_{AUTO}.

To pre-process the variables, we first log-transformed τ_{Post^t} and $\tau_{Post^{t-1}}$ (because the distribution of posting latency was highly skewed) and then scaled them within users (because we were interested in within-individual relationships). We calculated $\hat{R}_{t-1}$ as the mean reward (number of Likes) on the ten preceding posts ($t-2$ to $t-11$), consistent with the inferred predicted reward we used in the statistical signature of reward learning. Importantly, the true reward prediction is an internal latent variable calculated by the model, for which the relative contribution of more recent vs. less recent posts is determined by each user's individual reward learning rate. Therefore, including only the previous ten posts, and weighting them all equally, is an imperfect estimate of the model's reward prediction. Because this is imperfect, our linear regression underestimates the shared variance between successive posts which can be attributed to predicted reward, and therefore overestimates the non-reward related autocorrelation for pure RL models. We therefore did not expect $\beta_{AUTO}$to be 0 even for pure RL models. We inputted these variables into a Multilevel Model (MLM), to relate τ_{Post^t} to $\tau_{Post^{t-1}}$ controlling for $\hat{R}_{t-1}$. Users were random effects, with random slopes for both predictors ($\tau_{Post^{t-1}}$ and $\hat{R}_{t-1}$).

First, we visualized the fixed effect of β_{AUTO} in datasets simulated by different models (the same datasets as those used for the statistical signature of reward learning analysis) (Figure S6a). As expected, β_{AUTO} was greater than 0 even for the pure RL models because of the approximated nature of the estimate of reward prediction $\hat{R}_{t-1}$.

Next, to confirm that the habit component of our model functioned mechanistically as we expected, we examined the habit signature across users. We extracted the random effect of β_{AUTO} for each individual user, and examined how this habit signature predicted the value of the simulated habit weight parameter in the generated RLH1 dataset (the winning model in the AH confirmatory and dataset) (Figure S6b), and the fitted habit weight parameter in the empirical confirmatory sample (Figure S6c). β_{AUTO} significantly predicted simulated habit weight in the simulated RLH1 dataset (t(1556) = 6.166, p < 0.001, β = 0.154, 95% CI = [0.105, 0.204]) and fitted habit weight in the empirical dataset (t(1556) = 4.872, p < 0.001, β = 0.122, 95% CI = [0.073, 0.172]). This confirms that the simulated and fitted habit weight values are related to the extent to which individuals exhibit reward-independent action perseverance, a signature of habitual tendency.

Supplementary Note 3

Sensitivity analyses

i. Model comparison with different definitions of reward

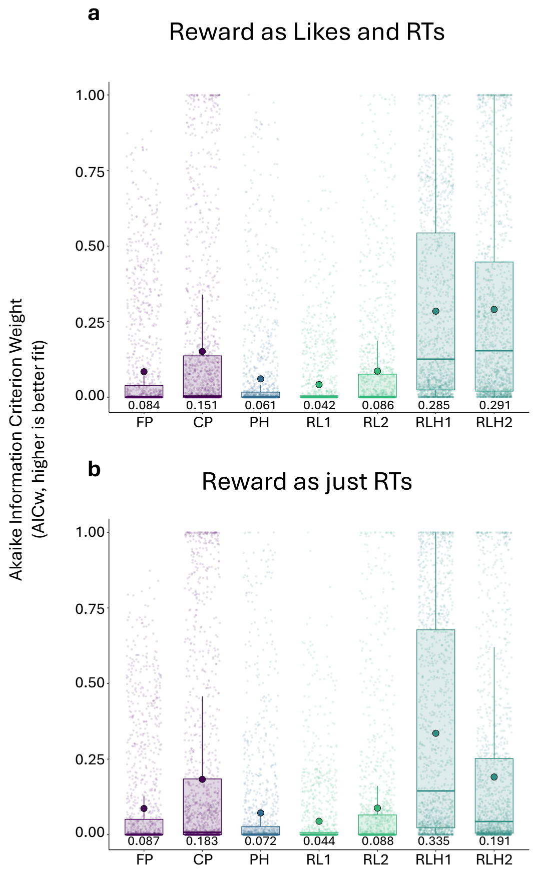

Figure S7. Model comparison with different definitions of reward in the confirmatory sample. FP = Fixed Policy, CP = Changing Policy, HP = Habitual Policy, RL1 = Pure RL, single learning rate, RL2 = Pure RL, double learning rate, RLH1 = Hybrid RL-habit, single learning rate, RLH2 = Hybrid RL-habit, double learning rate. Model comparison did not differ substantially if reward was conceptualized as the sum of Likes and RTs, or just RTs. Higher AICw represents a better fit. Outlined circles represent the mean AICw, which is also indicated as numbers along the top of the x-axis (the criterion for which best fitting model was selected), while box plots represent the median and interquartile range for AICw, and scattered points represent the AICw for each individual user. **a)** Model comparison results for models fit with reward as the sum of Likes and RTs. **b)** Model comparison results for models fit with reward as just RTs.

In our main analyses, we assumed that the ‘reward’ on each post was the number of Likes the post received. This is because we reasoned that post RTs can often be perceived as negative, whereas Likes are a purer measure of the positive subjective experience of a reward. To test the implications of this assumption, we re-conducted our main model comparison analysis with two alternative definitions of ‘reward’: the sum of Likes and RTs (as has been used in previous work on Twitter[25]), and just RTs. RL-habit hybrid models remained the winning models for both these definitions of reward, suggesting that our main results are robust to these different definitions of ‘reward’ (Figure S5).

ii. Model comparison with different policy distributions

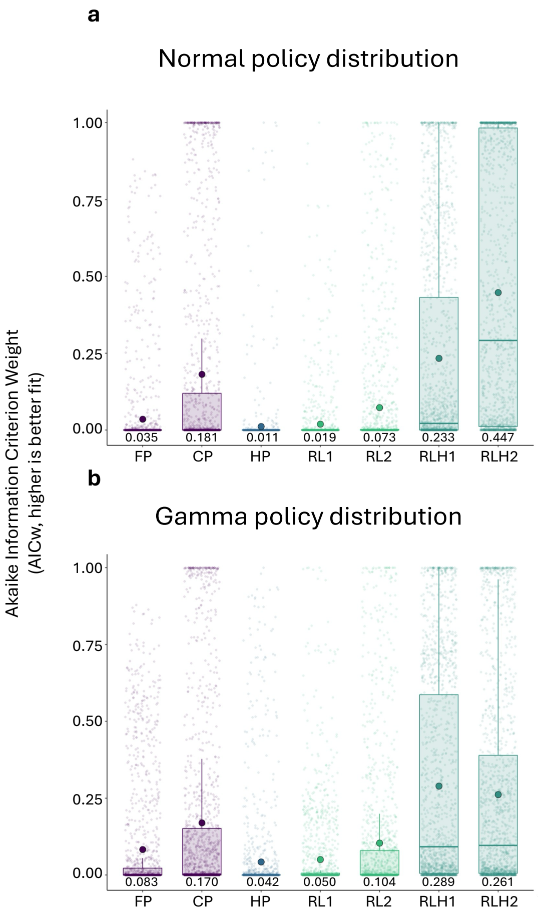

Figure S8. Model comparison with different policy distributions in the confirmatory sample. FP = Fixed Policy, CP = Changing Policy, HP = Habitual Policy, RL1 = Pure RL, single learning rate, RL2 = Pure RL, double learning rate, RLH1 = Hybrid RL-habit, single learning rate, RLH2 = Hybrid RL-habit, double learning rate. Model comparison did not differ substantially for a normal or gamma distribution, compared with the exponential distribution used in all analyses in the main paper. Specifically, hybrid RL-habit models still provided the best fit with both these variations. Higher AICw represents a better fit. Outlined circles represent the mean AICw, which is also indicated as numbers along the top of the x-axis (the criterion for which best fitting model was selected), while box plots represent the median and interquartile range for AICw, and scattered points represent the AICw for each individual user. **a)** Model comparison results for models fit with a normal policy distribution. **b)** Model comparison results for models fit with a gamma policy distribution.

In our main analyses, we assumed that each posting latency, τ_{Post^t}, was a random draw from an exponential distribution with expected value $Policy_t$ (Equation S9, which is the same as Equation 1 in the main paper):

Equation (S9) $$\tau_{Post^t} \sim Exponential\left(\frac{1}{Policy_t}\right)$$

To test the implications of this assumption, we repeated the model fitting and model comparison with two alternative policy distributions. The constraints for the distribution were that (1) the distribution must allow a continuous outcome variable (as posting latency is continuous), (2) τ_{Post^t} must be greater than 0, and (3) the expected value of the distribution would be $Policy_t$. First, we used a normal distribution with both mean (expected value) and standard deviation $Policy_t$, and for every τ_{Post^t} resampled from the distribution until τ_{Post^t} was greater than 0 (Equation S10).

Equation (S10) $$\tau_{Post^t} \sim Normal\left(Policy_t\,,\ {Policy_t}^2\right)$$

Next, we used a gamma distribution with both 'shape' and 'scale' parameters set to $\sqrt{Policy_t}$ (as the expected value of a gamma distribution is its shape parameter multiplied by its scale parameter) (Equation S11).

Equation (S11) $$\tau_{Post^t} \sim Gamma\left(\sqrt{Policy_t}\ ,\ \sqrt{Policy_t}\right)$$

Model comparison revealed that RL-habit hybrid models remained the winning models for both these policy distributions, suggesting that our main results do not depend on the specific policy distribution chosen (Figure S8).

iii. Model comparison with different initializations

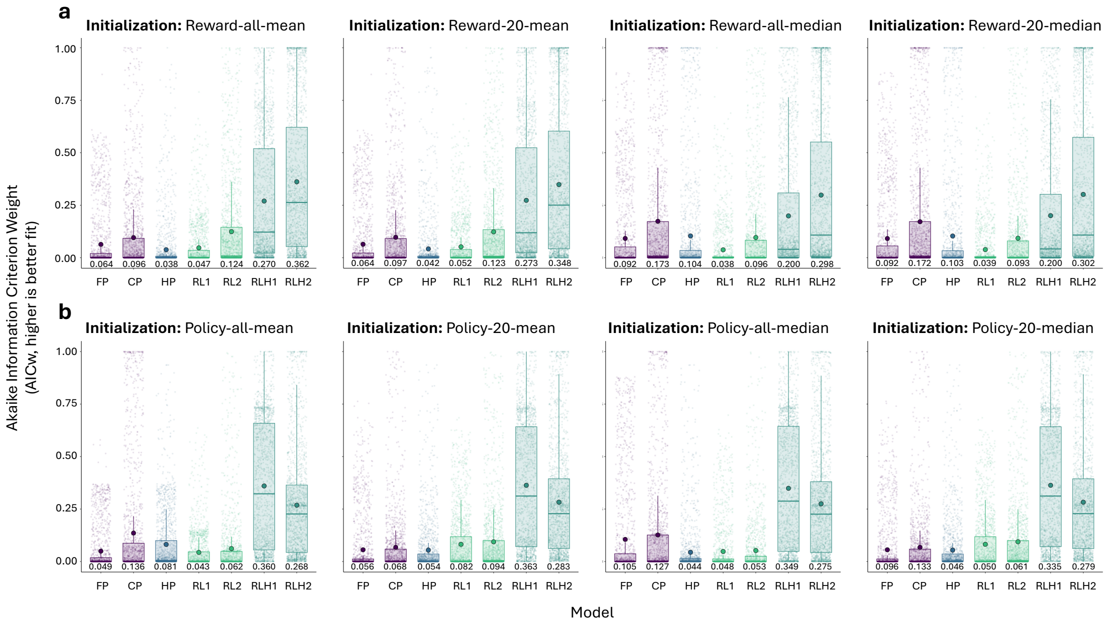

Figure S9. Model comparison with different initializations in the confirmatory sample. FP = Fixed Policy, CP = Changing Policy, HP = Habitual Policy, RL1 = Pure RL, single learning rate, RL2 = Pure RL, double learning rate, RLH1 = Hybrid RL-habit, single learning rate, RLH2 = Hybrid RL-habit, double learning rate. Model comparison did not differ substantially for eight different ways of estimating the initial policy and expected reward from the data. Higher AICw represents a better fit. Outlined circles represent the mean AICw, which is also indicated as numbers along the top of the x-axis (the criterion for which best fitting model was selected), while box plots represent the median and interquartile range for AICw, and scattered points represent the AICw for each individual user. **a)** Model comparison results for models fit using the rewards for each user to estimate starting values. **b)** Model comparison results for models fit using the posting latencies for each user to estimate starting values.

The RL, habit and hybrid RL-habit models all estimated start values for policy and expected reward from the data. The main paper describes how these were estimated for all main analyses (Methods). Here, to test whether our results were robust to the way in which these values were initialized, we re-ran model comparison with eight different ways of estimating both policy and expected reward from the data, which vary across three dimensions: (i) whether the rewards (Likes) or the posting latencies were used in the estimate, (ii) whether all posts in a user's data or the just first 20 posts

were used, and (iii) whether the mean or median of the relevant data points was used. Our results confirmed that our model comparison was robust to all these different ways of estimating initial expected reward and policy (Figure S9).

The eight different initialization schemes are as follows. In ‘Reward-all-mean’, the initial value for estimated reward in RL1, RL2, RLH1 and RLH2 was the mean of all rewards in that user’s data. The initial policy in RL1, RL2, RLH1 and RLH2, as well as the initial habit tendency in RLH1 and RLH2, was the vigour cost constant divided by this mean reward. The initial policy in the HP model was the same as in the main paper, the first posting latency for that user. In ‘Reward-20-mean’ all initializations were the same as ‘Reward-all-mean’ except the mean was taken of the first 20 rewards (Likes) for each user rather than all rewards. ‘Reward-all-median’ and ‘Reward-20-median’ were the same as ‘Reward-all-mean’ and ‘Reward-20-mean’, respectively, except that in each case medians were taken rather than means.

In ‘Policy-all-mean’, the initial value for estimated reward in RL1, RL2, RLH1 and RLH2 was the vigour cost constant divided by the mean of all posting latencies in that user’s data. The initial policy in RL1, RL2, RLH1, RLH2 and HP models, as well as the initial habit tendency in the RLH1 and RLH2 models, was also this mean of all posting latencies in that user’s data. Policy-20-mean, Policy-all-median and Policy-20-median were calculated by using the mean latency of the first 20 posts instead of all posts, the median of all posts, and the median of the first 20 posts, respectively.

iv. Model comparison with the RL model of Lindström et al. (2021)

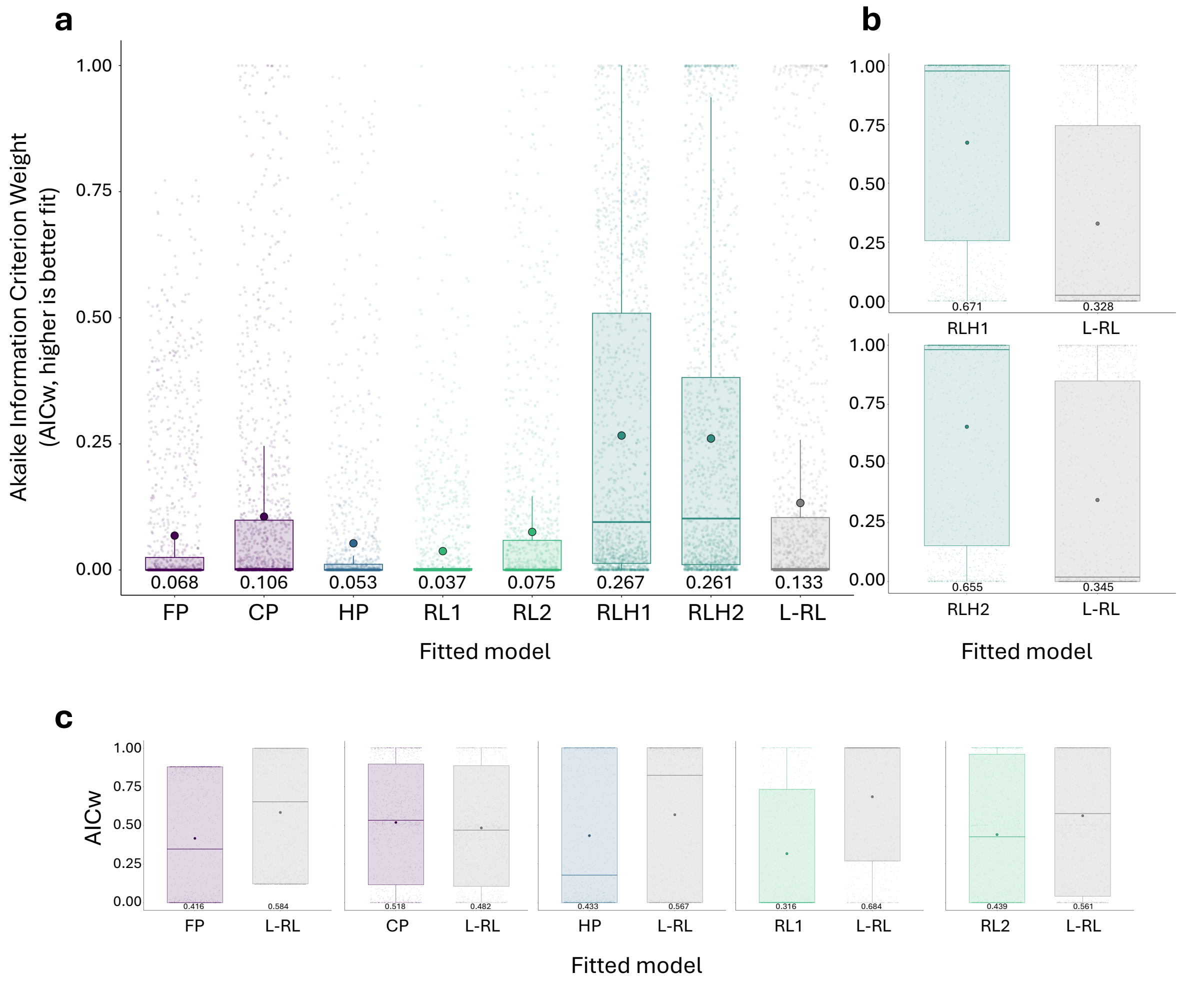

Figure S10. Model comparison with the Lindström RL model. FP = Fixed Policy, CP = Changing Policy, HP = Habitual Policy, RL1 = Pure RL, single learning rate, RL2 = Pure RL, double learning rate, RLH1 = Hybrid RL-habit, single learning rate, RLH2 = Hybrid RL-habit, double learning rate, L-RL = Lindström RL model. The RLH1 and RLH2 models provided better fits to the confirmatory sample than the Lindström RL model. Higher AICw represents a better fit. Outlined circles represent the mean AICw, which is also indicated as numbers along the top of the x-axis (the criterion for which best fitting model was selected), while box plots represent the median and interquartile range for AICw, and scattered points represent the AICw for each individual user. **a)** Model comparison results for all seven models from the main paper along with the Lindström RL model. **b)** Model comparison results for the RL-habit hybrid models each compared with the Lindström RL model, showing both of the RL-habit hybrid models

provide a better fit (higher AICw). **c)** Model comparison for all other models, each compared with the Lindström RL model.

Our modelling procedure was inspired by Lindström et al. (2021)[1], who were the first, to our knowledge, to adapt a model from the cross-species literature to describe a reinforcement learning process underlying posting on social media. Although inspired by the same animal model of action speed[2,3], our model differs from the Lindström RL model (see 'Model derivation' above for a complete description and justification).

To establish whether our winning model provided a better account of the cognitive processes underlying behaviour than the Lindström RL model, we fitted the Lindström RL model to our confirmatory Twitter dataset and compared its fit to all other models both together (Figure S10a) and individually (Figure S10b,c). Given that AICw identifies the best fitting model given the data and the set of candidate models, the comparison with each model individually allowed us to directly compare the performance of the Lindström model with each of our models in turn.

We first found that the Lindström RL model provided a better fit to our Twitter dataset than the Fixed Policy (FP) model. Given that the FP model is equivalent to the 'No Learning' model in Lindstrom et al. (2021)[1], this result replicates the main finding of Lindström et al. that their RL model provides a better fit than a FP model, in a new social media dataset (generalizing to Twitter from Instagram and forums) (Figure S10b). Importantly however, the Lindström RL model provided a worse fit to the data than both our RL-habit hybrid models (RLH1 and RLH2) (Figure S10c).

We demonstrated that this finding that our RL-habit models fitted the data better than the Lindström RL model was robust to different modelling decisions, by creating a new variant of the Lindström RL model (henceforth: 'new Lindström RL model') which

estimated initial policy from the data, by setting the initial policy as the mean of all posting latencies in each participant's dataset. Given that the original Lindström RL model fitted policy as a free parameter, this new variant had one fewer parameter than the original Lindström RL model, which might have been expected to give it more of an advantage in model fitting because the model fitting procedure, using Akaike Weights, penalized for each free parameter[24].

However, the new Lindström RL model fitted the data less well than the original Lindström RL model. Specifically, when fitting only the original and new Lindström RL models to the data, results were as follows: original: AICw = 0.648; new: AICw = 0.352. By extension, this new Lindström model also performed worse than both the RLH1 and RLH2 models when compared to each of them separately in turn. Comparing the new Lindström RL model to our RLH1 resulted in AICw = 0.787 for the RLH1 model compared with AICw = 0.213 for the new Lindström RL model, while comparing the new Lindström RL model to our RLH2 resulted in AICw = 0.761 for the RLH2 model compared with AICw = 0.239 for the new Lindström RL model. In summary, our models still fitted the data better than the Lindström RL model, even after removing a free parameter from the Lindström RL model.

v. Model falsification with different definitions of reward prediction error

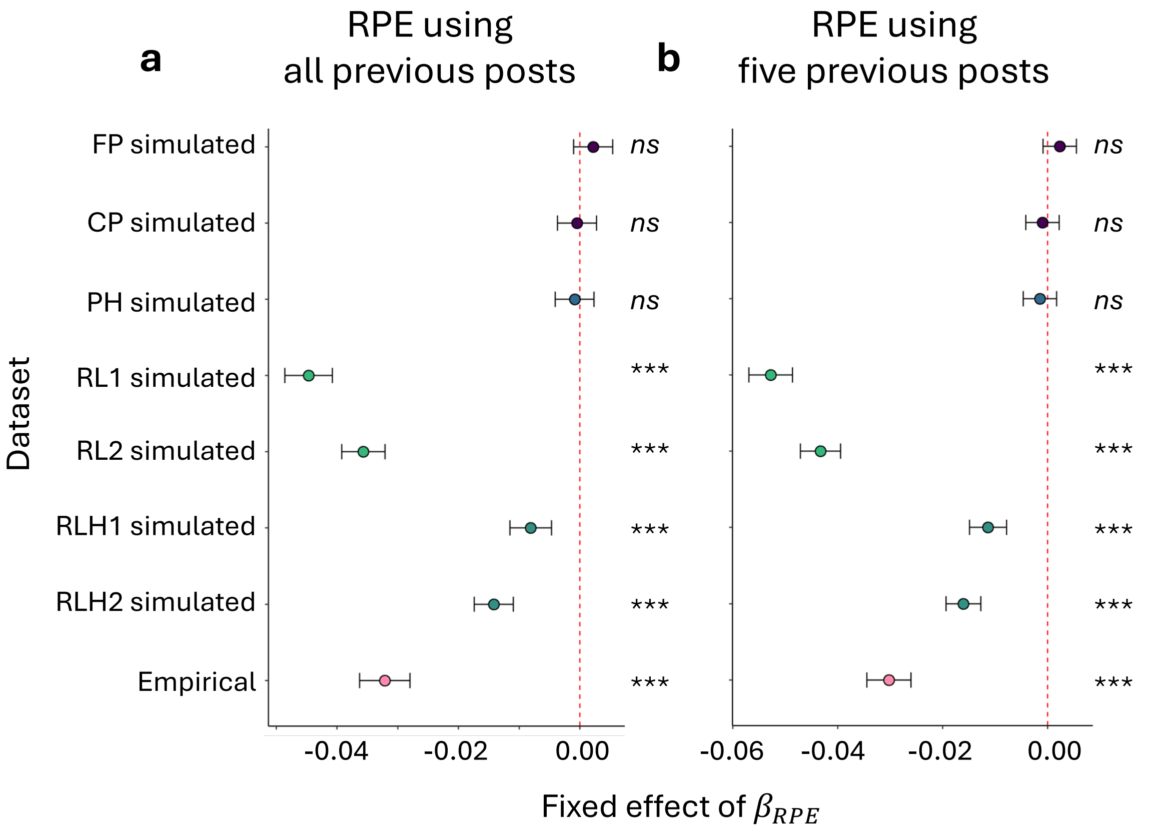

Figure S11. Model falsification with different definitions of reward prediction error (RPE). Dots show estimates of β_{RPE}, error bars show 95% confidence intervals. FP = Fixed Policy, CP = Changing Policy, HP = Habitual Policy, RL1 = Pure RL, single learning rate, RL2 = Pure RL, double learning rate, RLH1 = Hybrid RL-habit, single learning rate, RLH2 = Hybrid RL-habit, double learning rate. Model falsification results were not affected by varying the amount of trials considered for the inferred RPE. Specifically, the empirical dataset also shows a significantly negative fixed effect of β_{RPE}, which falsifies the hypothesis that the FP, CP or HP models underlie the data. **a)** Model falsification where RPE included all previous posts for each reward prediction. **b)** Model falsification where RPE included only the five previous posts for each reward prediction.

In the model falsification analysis in the main paper, we examined a model-agnostic statistical signature of reward learning by inferring the reward prediction error directly

from the data. Specifically, we calculated reward prediction error as the difference between the Likes received on the previous post, and the mean number of Likes on the preceding ten posts before that (the ‘prediction’).

This is an approximation to the true reward prediction error, as the amount of previous rewards experienced which contributes to each reward prediction would differ for each user depending on their individual reward learning rate. Therefore, to establish whether the model falsification results were dependent on the specific form of this approximation, we repeated the model falsification analysis with two alternative definitions of reward prediction: the mean number of Likes on all preceding posts for that user, and the mean number of Likes on the five preceding posts for that user. The model falsification results did not differ substantially for each of these alternatives, confirming that they are robust to these different formulations of the reward prediction approximation (Figure S11).

vi. Results from the RLH2 model

Our preregistration stated that we would conduct all analyses both for the RLH2 model, which won in the discovery sample, and for the winning model in the confirmatory sample. Given that RLH1 won in the confirmatory sample, we report all results for RLH1 in the main body of the paper, and for RLH2 in this section.

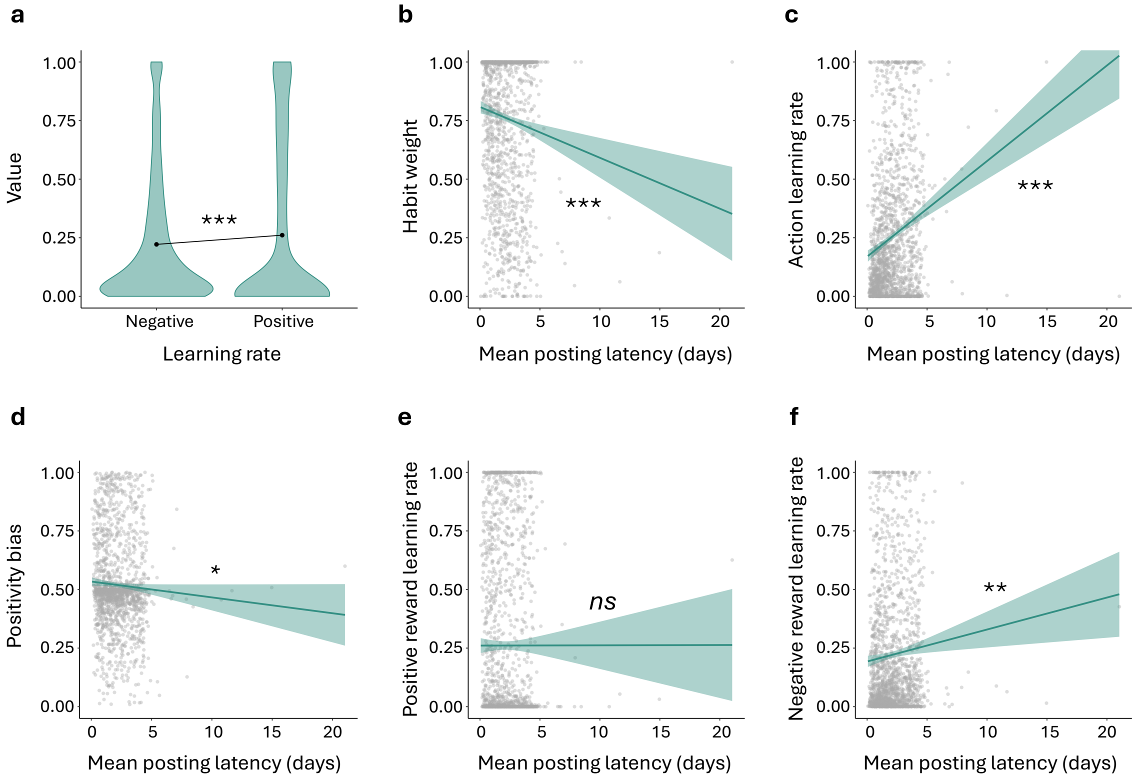

Figure S12. Positivity bias and relationship between computational parameters and posting latency using RLH2 parameters in the confirmatory sample. Annotations indicate significance, according to the following scheme: ns = not significant, * = $p < 0.05$, ** = $p < 0.01$, *** = $p < 0.001$. **a)** Violin plots compare RLH2 positive and negative reward learning rates. Dots represent the mean value of each reward learning rate. Positive reward learning rate was significantly greater than negative reward learning rate ($t(1557) = 3.911$, $p < 0.001$, $d = 0.099$, 95% CI = [0.049, 0.149]). **b)** Posting latency negatively predicted RLH2 habit weight ($t(1556) = -4.022$, $p < 0.001$, $\beta = -0.101$, 95% CI = [-0.151, -0.052]). **c)** Posting latency positively predicted RLH2 action learning rate ($t(1556) = 8.288$, $p < 0.001$, $\beta = 0.206$, 95% CI = [0.157, 0.254]). **d)** Posting latency negatively predicted the difference between RLH2 positive and negative reward learning rates (positivity bias) ($t(1556) = -1.917$, p(one-tailed) = 0.028, 95% CI = [-0.098, 0.001]). **e)** Posting latency had no significant relationship to RLH2 positive reward learning ($t(1556) = 0.018$, $p = 0.985$, $\beta = 0.000$, 95% CI = [-0.049, 0.050]). **f)** Posting latency positively predicted RLH2 negative reward learning rate ($t(1556) = 2.800$, $p = 0.005$, $\beta = 0.071$, 95% CI = [0.021, 0.120]).

We first investigated the valence bias using a paired-samples t-test (Figure S12a).

Participants showed a higher learning rate for positive compared to negative rewards in the confirmatory sample ($t(1557) = 3.911$, $p < 0.001$, $d = 0.099$, 95% CI = [0.049, 0.149]) (Figure S12a). We next investigated the relationship between posting latency and computational parameters across users. Users with lower posting latency had a higher habit weight ($t(1556) = -4.022$, $p < 0.001$, $\beta = -0.101$, 95% CI = [-0.151, -0.052]) (Figure S12b) and a lower action learning rate ($t(1556) = 8.288$, $p < 0.001$, $\beta = 0.206$, 95% CI = [0.157, 0.254]) (Figure 6c). Posting latency was negatively related to positivity bias in the confirmatory sample, given our preregistered one-tailed significance test ($t(1556) = -1.917$, p(one-tailed) = 0.028, $\beta = -0.049$, 95% CI = [-0.098, 0.001]) (Figure S12d).

Posting latency had no significant relationship to positive reward learning rate ($t(1556) = 0.018$, $p = 0.985$, $\beta = 0.000$, 95% CI = [-0.049, 0.050]) (Figure S12e), including after a meta-analysis across both samples ($z = -0.749$, $p = 0.454$, $\beta = -0.014$, 95% CI = [-0.052, 0.023]. However, users with lower posting latency did have a lower negative reward learning rate ($t(1556) = 2.800$, $p = 0.005$, $\beta = 0.071$, 95% CI = [0.021, 0.120]) (Figure S12f), suggesting that more frequent posters on Twitter are less likely to change their behaviour when they receive fewer posts than they expected. This remained significant, given our preregistered one-tailed test, after controlling for number of posts per person $t(1555) = 1.695$, p(one-tailed) = 0.045, $\beta = 0.054$, 95% CI = [-0.008, 0.116], partial regression coefficient for number of posts: $t(1555) = -0.877$, $p = 0.381$, $\beta = -0.028$, 95% CI = [-0.090, 0.034]) (see Preregistration Deviations).

We note that the findings relating to positivity bias should be interpreted with caution given that the winning model across the sample had a single learning rate (RLH1). Further, a positivity bias could plausibly arise from a model fitting procedure that assigns a high positive learning rate to high frequency posters, finding a local rather than global minimum for positive learning rate. In light of these concerns, replication of this result will be needed to confirm whether there is a true positivity bias in the empirical data.

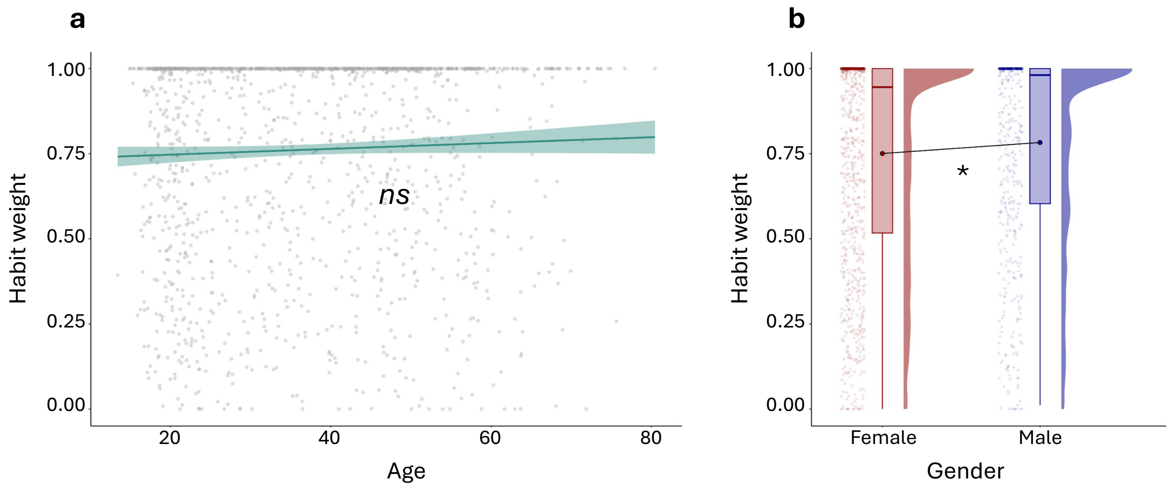

Figure S13. Relationship between habit weight and demographics using RLH2 parameters in the confirmatory sample. Annotations indicate significance, according to the following scheme: ns = not significant, * = p < 0.05, ** = p < 0.01, *** = p < 0.001. **a)** Age did not significantly predict RLH2 habit weight (t(1526) = 1.581, p = 0.114, β = 0.040, 95% CI = [-0.010, 0.091]). **b)** Males had a higher RLH2 habit weight than females (t(1221) = -2.043, p = 0.041, d = -0.106, 95% CI = [-0.209, -0.002]).

We next compared RLH2 habit weight to age and gender (Figure S7). Unlike RLH1, the relationship between age and habit weight was not significant (t(1526) = 1.581, p = 0.114, β = 0.040, 95% CI = [-0.010, 0.091]) (Figure S13a). However, gender was associated with habit weight: females had a lower habit weight than males (paired-samples t-test: t(1221) = -2.043, p = 0.041, d = -0.106, 95% CI = [-0.209, -0.002]) (Figure S13b).

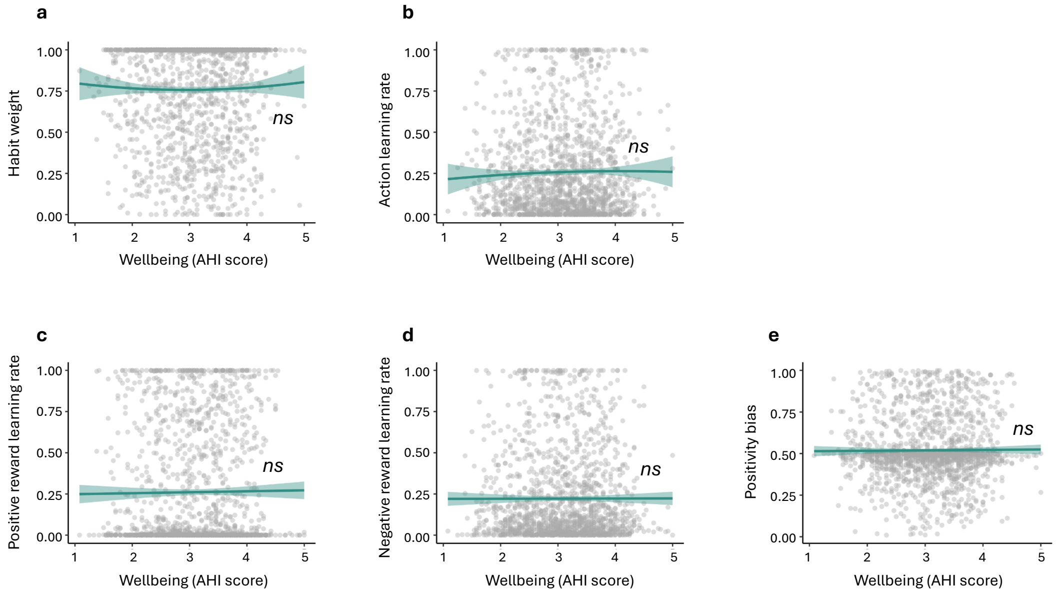

Figure S14. Relationship between computational parameters and wellbeing using RLH2 parameters in the confirmatory sample. Annotations indicate significance, according to the following scheme: ns = not significant, * = p < 0.05, ** = p < 0.01, *** = p < 0.001. **a)** Wellbeing had no significant linear or quadratic relationship to habit weight (linear: t(1555) = -0.785, p = 0.433, β = 0.008, 95% CI = [-0.024, 0.058], quadratic: t(1555) = 0.820, p = 0.412, Cohen's f^2 = 0.000, 95% CI (bootstrapped) = [0.000, 0.004]). **b)** Wellbeing had no significant linear or quadratic relationship to action learning rate (linear: t(1555) = 0.571, p = 0.568, β = 0.026, 95% CI = [-0.024, 0.076], quadratic: t(1555) = -0.429, p = 0.668, Cohen's f^2 = 0.000, 95% CI (bootstrapped = [0.000, 0.004]). **c)** Wellbeing had no significant linear relationship to positive reward learning rate ((t(1556) = 0.437, p = 0.662, β = 0.011, 95% CI = [-0.039, 0.061]). **d)** Wellbeing had no significant linear relationship to negative reward learning rate (t(1556) = 0.070, p = 0.944, β = 0.002, 95% CI = [-0.048, 0.052]). **e)** Wellbeing had no significant linear relationship to positivity bias (t(1556) = 0.349, β = 0.009, 95% CI = [-0.041, 0.059]).

Finally, we repeated the analyses relating RLH2 parameters to wellbeing. As with RLH1, we found no significant relationships between wellbeing and any computational parameters. AHI score did not significantly predict RLH2 habit weight (linear: t(1555) = -0.785, p = 0.433, β = 0.008, 95% CI = [-0.024, 0.058], quadratic: t(1555) = 0.820, p = 0.412, Cohen's f^2 = 0.000, 95% CI (bootstrapped) = [0.000, 0.004]) (Figure S14a). A meta-analysis across discovery and confirmatory samples also found no relationship between habit weight and wellbeing (linear: z = 0.658, p = 0.510, β = 0.013, 95% CI =

[-0.025, 0.051], quadratic: z = 0.798, p = 0.425, Cohen's f^2 = 0.003, 95% CI = [-0.005, 0.011]).

Wellbeing also did not significantly predict RLH2 action learning rate (linear: t(1555) = 0.571, p = 0.568, β = 0.026, 95% CI = [-0.024, 0.076], quadratic: t(1555) = -0.429, p = 0.668, Cohen's f^2 = 0.000, 95% CI (bootstrapped = [0.000, 0.004]) (Figure S14b). A meta-analysis across discovery and confirmatory samples also found no significant relationship for either the linear (z = 0.021, p = 0.983, β = 0.000, 95% CI = [-0.053, 0.054]) or quadratic (z = 0.531, p = 0.595, Cohen's f^2 = 0.000, 95% CI = [-0.001, 0.002]) terms.

We also found no significant relationship between wellbeing and either positive reward learning rate (t(1556) = 0.437, p = 0.662, β = 0.011, 95% CI = [-0.039, 0.061]) or negative reward learning rate (t(1556) = 0.070, p = 0.944, β = 0.002, 95% CI = [-0.048, 0.052]) (Figures S14c,d). Meta-analyses across both samples also found no significant relationship for positive reward learning rate (z = -0.651, p = 0.515, β = -0.024, 95% CI = [-0.095, 0.047]) or negative reward learning rate (z = -0.900, β = -0.031, 95% CI = [-0.098, 0.037]). Finally, we found no significant relationship between positivity bias and wellbeing (t(1556) = 0.349, β = 0.009, 95% CI = [-0.041, 0.059]) (Figure S14e). A meta-analysis across both samples also also found no significant relationship (z = 0.081, p = 0.936, β = 0.002, 95% CI = [-0.036, 0.039].

vii. Posting latency correlations with outliers removed

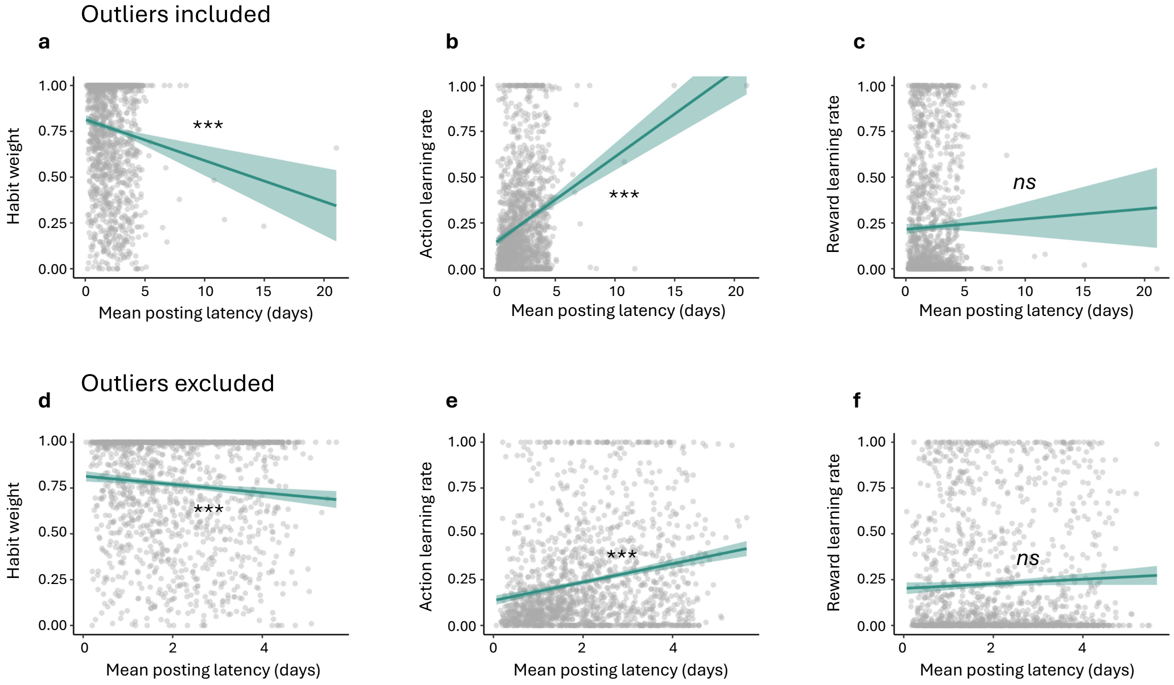

Figure S15. Relationship between computational parameters and posting latency with and without posting latency outliers. Annotations indicate significance, according to the following scheme: *ns* = not significant, * = $p < 0.05$, ** = $p < 0.01$, *** = $p < 0.001$. Figures **a)** to **c)** are the same as Figures 4a-c in the main paper, with the x axis extended to include outliers. **a)** Posting latency negatively predicted habit weight ($t(1556) = -4.284$, $p < 0.001$, $\beta = -0.108$, 95% CI = [-0.157, -0.059]). **b)** Posting latency positively predicted action learning rate ($t(1556) = 9.955$, $p < 0.001$, $\beta = 0.245$, 95% CI = [0.196, 0.293]). **c)** Posting latency did not significantly predict reward learning rate rate ($t(1556) = 0.948$, $p = 0.343$, $\beta = 0.024$, 95% CI = [-0.026, 0.074]). **d)** Posting latency negatively predicted habit weight in the confirmatory sample with outliers removed ($t(1544) = -3.659$, $p < 0.001$, $\beta = -0.093$, 95% CI = [-0.142, -0.043]). **e)** Posting latency positively predicted action learning rate in the confirmatory sample with outliers removed ($t(1544) = 9.040$, $p < 0.001$, $\beta = 0.224$, 95% CI = [0.176, 0.273]). **f)** Posting latency did not significantly predict reward learning rate in the confirmatory sample with outliers removed ($t(1544) = 1.797$, $p = 0.073$, $\beta = 0.046$, 95% CI = [-0.004, 0.096]).

The posting latency across users had outliers in both the discovery and confirmatory sample. Figure 4 in the main paper shows the x-axis cropped without these outliers; Figures S15a-c show the same relationships with outliers included.

To confirm that our results were not skewed by these outliers, we conducted a sensitivity analysis for all posting latency results, with all users with a posting latency over 1.5 times the interquartile range above the upper quartile or below the lower quartile removed. We used parameters from RLH1, the winning model in the confirmatory sample. Findings remained significant with outliers excluded: posting latency negatively predicted habit weight ($t(1544) = -3.659$, $p < 0.001$, $\beta = -0.093$, 95% CI = [-0.142, -0.043]) (Figure S15d) and positively predicted action learning rate ($t(1544) = 9.040$, $p < 0.001$, $\beta = 0.224$, 95% CI = [0.176, 0.273]) (Figure S15e), but was not significantly related to reward learning rate ($t(1544) = 1.797$, $p = 0.073$, $\beta = 0.046$, 95% CI = [-0.004, 0.096]) (Figure S15f), including after a meta-analysis across discovery and confirmatory samples ($z = 1.261$, $p = 0.207$, $\beta = 0.027$, 95% CI = [-0.015, 0.069]).

Supplementary Note 4

Preregistration deviations

We made three deviations from our preregistered analysis plan[26], documented in Table S2.

Original plan	Revised plan	Justification for deviation
When testing for the reward learning behavioural signature, we originally planned not to standardize the dependent variable, as stated in the preregistration on page 12[26]: ***'To compute $\Delta\tau_{Post^t}$ for use in the GLM, we will first take the logarithm of τ_{Post^t} and $\tau_{Post^{t-1}}$, and then subtract $\tau_{Post^{t-1}}$ from τ_{Post^t}. We will then use a Generalized Linear Model (GLM) with random effects for user, to use $\delta_{R^{t-1}}$ to predict $\Delta\tau_{Post^t}$, extracting a coefficient β_{RPE} for each user.'***	We mean-centered and standardized $\Delta\tau_{Post^t}$ within users.	We had mean-centered and standardized the predictor variable within users. As our research question concerned how a reward prediction error predicted a change in posting latency within individuals, both variables should be standardized to within-individual values.
We originally intended to use only model comparison to select the winning model.	We also carried out model falsification to verify model selection.	Model falsification provides a complementary absolute criterion for behavioural model comparison, to the relative criterion in model comparison, strengthening our conclusions about the winning model[23].
We originally intended to compare individual differences (i.e., posting latency, age, gender, and wellbeing) to parameters without controlling for number of posts.	We added sensitivity analyses controlling for number of posts per participant in all linear models relating individual differences to model parameters in the main paper.	We discovered in a simulated dataset that number of posts was related to both the fitted habit weight and action learning rate parameters of the RLH1 model, suggesting that relationships between

		frequency and other individual differences could be partly influenced by aspects of the model fitting procedure. We therefore now add new analyses to all individual-difference analyses in the main paper, controlling for number of posts to verify that our individual differences results were not driven by this artefact of the fitting procedure.

Table S2. List of preregistration deviations and justifications.

References

1. Lindström, B. *et al.* A computational reward learning account of social media engagement. *Nat Commun* **12**, 1311 (2021).

2. Niv, Y., Daw, N., & Dayan, P. How fast to work: Response vigor, motivation and tonic dopamine. *Advances in neural information processing systems,* 18 (2005).

3. Niv, Y., Daw, N. D., Joel, D. & Dayan, P. Tonic dopamine: opportunity costs and the control of response vigor. *Psychopharmacology* **191**, 507–520 (2007).

4. Staddon, J. E. R. *Adaptive Dynamics: The Theoretical Analysis of Behavior*. xiv, 423 (The MIT Press, Cambridge, MA, US, 2001). doi:10.7551/mitpress/1092.001.0001.

5. Towner, E., Grint, J., Levy, T., Blakemore, S.-J. & Tomova, L. Revealing the self in a digital world: A systematic review of adolescent online and offline self-disclosure. *Current Opinion in Psychology* **45**, 101309 (2022).

6. Mirea, D.-M. *et al.* Depression is associated with higher sensitivity to social media rewards. Preprint at https://doi.org/10.31234/osf.io/4ynbc (2024).

7. Brady, W. J., McLoughlin, K., Doan, T. N. & Crockett, M. J. How social learning amplifies moral outrage expression in online social networks. *Sci Adv* **7**, eabe5641 (2021).

8. Camerer, C., Xin, Y. & Zhao, C. A neural autopilot theory of habit: Evidence from consumer purchases and social media use. *Journal of the Experimental Analysis of Behavior* **121**, 108–122 (2024).

9. Webb, R. *et al.* Integrating Neuro-Psychological Habit Research into Consumer Choice Models. SSRN Scholarly Paper at https://doi.org/10.2139/ssrn.4853969 (2024).

10. Miller, K. J., Shenhav, A. & Ludvig, E. A. Habits without Values. *Psychol Rev* **126**, 292–311 (2019).

11. Thorndike, E. L. *Animal Intelligence: Experimental Studies*. viii, 297 (Macmillan Press, Lewiston, NY, US, 1911). doi:10.5962/bhl.title.55072.

12. Dickinson, A. Actions and Habits: The Development of Behavioural Autonomy. *Philosophical Transactions of the Royal Society of London. Series B, Biological Sciences* **308**, 67–78 (1985).

13. Hull, C. L. *Principles of Behavior: An Introduction to Behavior Theory*. x, 422 (Appleton-Century, Oxford, England, 1943).

14. Wood, W., Mazar, A. & Neal, D. T. Habits and Goals in Human Behavior: Separate but Interacting Systems. *Perspect Psychol Sci* **17**, 590–605 (2022).

15. Greenstreet, F. *et al.* Dopaminergic action prediction errors serve as a value-free teaching signal. *Nature* 1–10 (2025) doi:10.1038/s41586-025-09008-9.

16. Doll, B. B., Simon, D. A. & Daw, N. D. The ubiquity of model-based reinforcement learning. *Curr Opin Neurobiol* **22**, 1075–,1081 (2012).

17. Daw, N. D. & O'Doherty, J. P. Chapter 21 - Multiple Systems for Value Learning. in *Neuroeconomics (Second Edition)* (eds Glimcher, P. W. & Fehr, E.) 393–410 (Academic Press, San Diego, 2014). doi:10.1016/B978-0-12-416008-8.00021-8.

18. Dolan, R. J. & Dayan, P. Goals and Habits in the Brain. *Neuron* **80**, 312–325 (2013).

19. Daw, N. D., Gershman, S. J., Seymour, B., Dayan, P. & Dolan, R. J. Model-based influences on humans' choices and striatal prediction errors. *Neuron* **69**, 1204–1215 (2011).

20. Gremel, C. M. & Costa, R. M. Orbitofrontal and striatal circuits dynamically encode the shift between goal-directed and habitual actions. *Nat Commun* **4**, 2264 (2013).

21. Anderson, I. A. & Wood, W. Social motivations' limited influence on habitual behavior: Tests from social media engagement. *Motivation Science* **9**, 107–119 (2023).

22. Wilson, R. C. & Collins, A. G. Ten simple rules for the computational modeling of behavioral data. *eLife* **8**, e49547 (2019).

23. Palminteri, S., Wyart, V. & Koechlin, E. The Importance of Falsification in Computational Cognitive Modeling. *Trends in Cognitive Sciences* **21**, 425–433 (2017).

24. Wagenmakers, E.-J. & Farrell, S. AIC model selection using Akaike weights. *Psychon Bull Rev* **11**, 192–196 (2004).

25. Anderson, I. A. Beyond Active and Passive Social Media Use: Habit Mechanisms Are Behind Frequent Posting and Scrolling on Twitter/X. (UNIVERSITY OF SOUTHERN CALIFORNIA, 2024).

26. Turner, G. Habits and Reward Learning on Twitter: A Computational Approach. https://doi.org/10.17605/OSF.IO/JYBHX (2024) doi:https://doi.org/10.17605/OSF.IO/JYBHX.
