# Supplementary material for: A computational model of reward learning and habits on social media
Source: Nat Commun. 2026 Jun 4;17:7170. doi: 10.1038/s41467-026-73547-6 (PMC13396645; doi:10.1038/s41467-026-73547-6)
Supplement: Supplementary file 3 — Transparent Peer Review file [file 41467_2026_73547_MOESM3_ESM.pdf]

nature portfolio

Peer Review File

A computational model of reward learning and habits on social media

Corresponding Author: Ms Georgia Turner

This file contains all reviewer reports in order by version, followed by all author rebuttals in order by version.

Version 0:

Reviewer comments:

Reviewer #1

(Remarks to the Author)
The paper by Turner et al is an interesting contribution to the literature and seems to be an excellent result of a fruitful collaboration between computational modelling expert (Palminteri) and a leader in (youth) social media research (Orben). The model is very interesting and their approach including a preregistered replication sample is thorough and impressive. I have a few points that the authors may want to consider:
• Interpretation of the models: the authors very strongly term the latency model as “habitual” and the other as goal-directed. However, the latter has often been (falsely?) termed habitual (e.g. Claire Gillan’s work) rather than goal-directed. I believe it would be worth discussing the terminology a bit more and provide a clear definition of “habitual” as one could argue that any form of learning would not be habitual (i.e. only the FP model would be habitual).
• Model boundary effects: When looking at Figs 4 and 5, it is striking how many parameters land in the boundaries (e.g. Fig 4a means that most people are fully “habitual). This points to some convergence issues. The authors may consider using hierarchical or MAP methods to get more reliable parameter estimates (which may improve the individual-differences findings). I also did not see a parameter recovery matrix, but I might have missed it.
• It would be nice to have more descriptives of the sample, e.g. how many posts per person, how many likes per post, variability of posts, variability of posting latency, etc.
• The model equations would be a bit more explained, esp eq. 3-5
• Did the authors consider alternative policy distributions other than just an (inverse) exponential, e.g. Poisson distribution?
• It would be nice to have an indication for significances in the Figures. I believe that Fig 1b is not essential.
• There seems a typo for the replication sample N: Intro: N=1558, Results: N=1158
• I could not find any data availability statement

(Remarks on code availability)

Reviewer #2

(Remarks to the Author)
Review: ”A computational model of reward learning and habits on social media”

In this paper, the authors propose a computational model that combines reward learning and habit-like responding and compare this model to a set of alternative models in two Twitter data sets. The results indicate that Twitter users are both sensitive to rewards and tend to repeat previous actions (response latencies). They also provide evidence for some differences between men and women, and younger versus older users in the weight of the two processes. There is a lot to like about this paper: it tackles important questions and uses rigorous modeling methods, combined with model-independent analysis of reward signatures. Overall, this is very good work, which will interest a broad readership.

However, I have several concerns and questions that merit clarifications or additional analysis (none of which are grounds for rejection).

Model formulation. The authors derive their model from an earlier model developed by Niv. Specifically, they base it on the

equations linking the optimal response rate to the average reward rate (which depends on expected immediate rewards, the number of responses required, costs etc) in a ratio schedule. They make a number of simplifying assumptions and end up with a model where the response latency is inversely proportional to the expected reward and directly proportional to a "cost" parameter. While this is motivated by Niv's theory, it results in a model that only cares about the average reward but is insensitive to time. This makes sense in the context of a ratio schedule but falls short in other cases. For example, consider the case where the likes a Twitter user receives depend on their posting latencies (e.g., R = 20 if 10 < latency < 20 and R = 1 otherwise, or some smoothed version thereof), I don't see how the authors model could not learn this optimal latency. The only way for it's policy to fall in the optimal range is by adjusting the cost parameter (and the initial reward), which does not seem satisfactory. The same issue would presumably arise on interval schedules. While I do not think this is a critical flaw of the paper, I do think it requires a careful description of the limits of the model, and acknowledging that even though it seems to describe user behavior quite well, it might fall short as a more complete account social media posting (I assume that humans can easily learn such things, and probably often do).

Description of habit component. The authors motivate their habit model based on the "habits without values", and refer to a "stickiness" parameter. While I do get what the authors mean, the "stickiness" parameter just determines the memory of a moving average of previous posting latencies – i.e calling it stickiness seems a bit odd. Although I am open to being convinced differently. Relatedly, the Miller model bases habits on frequencies of different actions (e.g., action A 10 times, action B 100 times). As described, the authors' model formulation simplifies this a lot with the moving average formulation, which just says that posting latencies will be similar to previous posting latencies. Alternatively, one could count the number of posts in various time bins (corresponding to actions), which would determine the habit strength. Although I guess this in practice would be quite similar. In summary, I ask the authors to be a bit more transparent in how their model and choices correspond to the motivating literature. No need to oversell.

Model falsification. I like this part a lot. A couple of questions: how sensitive are the results to the exact definition of the reward signature (e.g., the number of trials considered for the PE)? The size of the fixed effects estimate in the data (-.031) seems to be more similar to the pure RL models than the RL + Habit models (-0.012 & -0.016). Could you discuss that? Finally, and most importantly: are there any model-independent signatures of "habitual" responding? Can you distinguish between pure RL and RL + Habit based on such a signature? It might be difficult, but would be a strong addition. However, not crucial for the success of the paper.

Habitual users. The authors note that users with more posts tend to be fit with a higher habit weight. It would be good to confirm that this is not an artifact of the fitting procedure, e.g., by simulating and fitting data based on the same parameter values, but with different number of posts. The question would then be if more posts => higher habit weight.
Parameter values. It would be great with a table with the average estimated parameter values for the different models and samples.
Finally, I could not find any information about any start value for the policy or the reward estimate.

(Remarks on code availability)

Reviewer #3

(Remarks to the Author)
This paper puts forward a hybrid model of reinforcement learning and habitual processes involved in social media posting behavior. I applaud the authors for their creative thinking and their research practices, including an extensive (and adhered to!!!) preregistration, extensive model simulations, model comparison and model falsification. Their modeling code seems well-organized and well-commented, aiding use of their model (although I have not tried). Although I think the authors delivered a great paper already, I do have some considerations for them to make the paper clearer and even more convincing.

Major points:

Introduction

1. The introduction is clear and well-written. As somebody outside of the social media field, some sentences require additional explanation. For example, I can picture what you mean with "diversity" in lines 77/78 "Critically, the dose-response approach can neither account for the diversity of social media users and behavior", but in the part after that – "nor generate findings which are robust to the constantly changing social media landscape." – I struggle to visualize "the constantly changing social media landscape". The same holds for "However, there are many ways in which this statistical signature might be operationalized" (lines 133/134) and for the "behavior-environment mismatch" on lines 89/90. Examples would help.

Method

2. How were values (both in the RL part and in the Habit part of the model) initialized? It doesn't seem so straight forward to decide which initial expectation individuals have about receiving likes while this initialization can have a large effect on (asymmetric) learning rates.

Results

3. I really appreciate you preregistering the project, sticking to the preregistration, and clearly reporting deviations in the supplements. Yet, I wonder whether relating posting frequency to habitual model parameters is more of a model check then a primary goal, and should therefore have a less prominent place in the paper.

4. I believe that the evidence for a positivity bias is very limited and that claims should therefore be removed throughout. The RLH1 outperforms the RLH2 in the confirmatory sample and, judging from the plot in Figure 2 of the preregistration, the difference in learning rates in the discovery sample is negligible (as also reflected in the 0.049 p-value). Although you do state that “the findings relating to positivity bias should be interpreted with caution given that the winning model across the sample has a single learning rate (RLH1)”, this is only done so in the supplements and claims about the positivity bias within the text are unnuanced.

5. This may be a plotting function issue, but the regression lines (especially in Figure 5C/5D) suggest non-linearity to me. Did you test for linear as well as non-linear relationships? If so, this is missing in the text. If not, why did you only do this for wellbeing?

Discussion

6. With the current societal discussion on individual differences in effects of social media use, I see it as a missed opportunity to not quantify individual differences in model parameters or even in the cognitive processes involved in social media use. You already have these data because you fit models individually and, judging from the model parameter plots in the supplements and from the model fit plot in Figure 3A, individual differences in both are substantial. I understand you cannot do everything in one paper, but as you do relate individual differences to demographics and well-being, mentioning individual differences in the discussion would already bring added value.

7. I find the discussion somewhat uncritical. The title suggests that the main goal of the project is to put forward “a computational model of reward learning and habits on social media”. Because of this, I expected you to discuss the shortcomings of the model. Although you do mention these, you only do so in the supplements (line 605-607: “We document the specific simplifying assumptions of our model in the Supplementary Materials, so that future work can reintroduce parameters into the model to account for more complexity.”).

8. Relatedly, you state that “model recovery was excellent” with 1000 posts. I don’t think I agree. If I look at the column of the best-fitting model (RLH1) in Figure S1b, the AIC weight of the corresponding model (RLH1) is about .5. The AIC weight of the PH about .3. Dividing these values gives you that the RLH1 is only 1.66 times more likely to be the underlying model than the PH. I wouldn’t call that excellent, especially if the average number of posts is lower than 1000.

I don’t want to be nitpicky and I think you have already done enough simulation work, but if I’m a social media researcher without much modeling experience that wishes to apply your hybrid model, I would want to be sure that it is recoverable. If it is barely recoverable at 1000 posts, I would be worried. I suggest providing some discussion on this point. What is your feeling about the required number of posts based on your simulations?

Minor points:

9. Even after reading the supplemental materials, I’m having trouble visualizing what vigour costs mean. I suppose you need the vigour cost parameter to allow for differences in the relationship between expected rewards and policies across individuals, but what this parameter captures in real life is vague to me. Please elaborate.

10. I may have missed this but what was the average number of posts? It would help to add this to Table 1 in order to put the simulation results (in which you used 80 and 1000 posts) into perspective.

11. I would consider changing some of the model names. I (and probably other RL modelers) associate PH with the Pearce-Hall model. Besides, the P sometimes means “pure” (as in PH) and sometimes “policy” (as in CP).

12. You made lovely plots. For that reason (and for conciseness), I would remove Table 2 (which information is already in Figure 3A) and put test statistics in the plots instead of in the text, which often doubles the information in the plots (e.g., Figure 3B and the paragraph starting at line 370 “As expected…”).

13. The reason for investigating how individual differences in model parameters relate to age, gender, and wellbeing is unclear to me. Why these variables and why no interactions for example?

14. Some typos I noticed: line 244 (Hih), line 440 (interquartlie), line 453 (“paired-samples” should be “independent-samples”?), line 708 (from from), Figure S4 (y-axis should be “Random” instead of “Fixed” effect?) and below that on line 301 (sample sample).

To promote constructive reviewing and transparency, I always sign my reviews,
Jessica Schaaf

(Remarks on code availability)

A quick glance suggests the code is well-organized and well-commented. README file is present. I did carefully compare the preregistration to the manuscript. A breath of fresh air. Precise and extensive. Almost no deviations, and if there were, they were outlined including reasoning in the supplements.

Version 1:

Reviewer comments:

Reviewer #1

(Remarks to the Author)
I thank the authors for their thorough revision of the manuscript and for addressing all issues that I've raised previously.

(Remarks on code availability)

Reviewer #2

(Remarks to the Author)
The authors have done a great job overall with the review, and generally, all my original questions have been addressed. Congrats on a great paper!

Regarding the model formulation point, I have an additional concern:

The authors now fit the model described in Lindström et al 2021 to their data, and found their RL+Habit model to be better. The "Lindström model" included a parameter to set the initial policy, while the RL+Habit models did not (where the initial policy depends on the initial reward estimate, which the authors defined in a variety of different ways). This seems like an unfair comparison - it would therefore be instructive to fit the "Lindström model" using the same/similar approaches to initialize the policy as the RL+Habit models, thereby reducing the number of free parameters.

(Remarks on code availability)

Reviewer #3

(Remarks to the Author)
Thank you for the thorough revision. You have addressed all my points adequately. I don't have additional ones. It has become (and already was) a great paper. You may all be very proud of this work that hopefully sets the stage to increasingly use RL models to investigate real-life learning behavior.

(Remarks on code availability)

Version 2:

Reviewer comments:

Reviewer #2

(Remarks to the Author)
I have no more concerns or questions. Congrats on a great paper!

(Remarks on code availability)

Open Access This Peer Review File is licensed under a Creative Commons Attribution 4.0 International License, which permits use, sharing, adaptation, distribution and reproduction in any medium or format, as long as you give appropriate credit to the original author(s) and the source, provide a link to the Creative Commons license, and indicate if changes were made.
In cases where reviewers are anonymous, credit should be given to 'Anonymous Referee' and the source.
The images or other third party material in this Peer Review File are included in the article's Creative Commons license, unless indicated otherwise in a credit line to the material. If material is not included in the article's Creative Commons license and your intended use is not permitted by statutory regulation or exceeds the permitted use, you will need to obtain permission directly from the copyright holder.
To view a copy of this license, visit https://creativecommons.org/licenses/by/4.0/

A computational model of reward learning and habits on social media
Georgia Turner, Lukas J. Gunschera, Shashanka Subrahmanya, Aadesh Salecha, Johannes C. Eichstaedt, Stefano Palminteri* and Amy Orben*
* = joint senior authorship

Nature Communications: Reviewer Response

REVIEWER COMMENTS

Reviewer #1 (Remarks to the Author):

The paper by Turner et al is an interesting contribution to the literature and seems to be an excellent result of a fruitful collaboration between computational modelling expert (Palminteri) and a leader in (youth) social media research (Orben). The model is very interesting and their approach including a preregistered replication sample is thorough and impressive.

We thank R1 for their appreciation of our work.

I have a few points that the authors may want to consider:

R1.1
Interpretation of the models: the authors very strongly term the latency model as “habitual” and the other as goal-directed. However, the latter has often been (falsely?) termed habitual (e.g. Claire Gillan’s work) rather than goal-directed. I believe it would be worth discussing the terminology a bit more and provide a clear definition of “habitual” as one could argue that any form of learning would not be habitual (i.e. only the FP model would be habitual).

R1 is right to point out that our mapping from the concepts in classical psychology of goal-directed and habitual control, to reinforcement learning algorithms, contrasts with the previously accepted view that equates habit with model-free reinforcement learning, and goal-directed control with model-based reinforcement learning. Instead, our definition of habit is grounded in a definition propounded by (Miller et al., 2019) and others, and stemming from Thorndike’s Law of Exercise for habit: that an action that has been taken often in the past is likely to be repeated in the future (Thorndike, 1911). This view defines habit as a value-free process, and all reinforcement learning (both model-free and model-based), as a goal-directed process. Thus, the distinction between habit and goal-directed processes is not one between learning and not learning, but between value-free learning (habit) and value-based learning (goal-directed, such as RL).

The definition we use is supported both by theoretical and neurobiological research in psychology, and by empirical research into how habits manifest in social media posting. To help clarify why we believe this view to be correct, as well as to make it more explicit that this contrasts with the definition used in previous work by Nathaniel Daw, Claire Gillan and others, we have added more detailed justifications in three places.

The description of the models in Methods:

> *“Habitual Policy model (HP). The Habitual Policy model is based on a definition of ‘habit’ as a value-free process, following Thorndike’s (1911) Law of Exercise to define habits: that an action that has been taken often in the past is likely to be repeated in the future(Miller et al., 2019; Thorndike, 1911). Unlike RL, which involves value-based learning, habits learn from past actions in a form of value-free learning, resulting in perseveration or repetition of previous actions.”* (page 31, lines 877-882)

The description of the model derivation in the Supplementary:

> *“Our habit models are conceptually inspired by the computational model of animal habits put forward by Miller et al (2019)(Miller et al., 2019). Specifically, habits are conceived of as a value-free process which takes the form of perseveration, aligning with Thorndike’s (1911)(Thorndike, 1911) Law of Exercise: that an action that has been taken often in the past is likely to be repeated in the future.*
>
> *This theoretical definition is used in much traditional classical psychology research, which suggests that, although habits are initially formed when repeating rewarded actions, they represent stimulus-response associations(Dickinson, 1985; Hull, 1943). Stimulus-response associations contrast from response-outcome associations(Wood et al., 2022), as in both model-free and model-based RL. Thus, habits are a form of learning, but the learning pertains to stimulus-response (value-free) rather than response-outcome (value-based) associations(Greenstreet et al., 2025; Miller et al., 2019).*
>
> *This definition of habit as a value-free process differs from a previously proposed view in the computational RL literature, which mapped habits onto model-free RL, and goal-directed behaviour onto model-based RL*(Daw & O’Doherty, 2014; Dolan & Dayan, 2013; Doll et al., 2012)*. However, we believe*

that this previous definition is in contrast with both neural and behavioural evidence supporting the value-free habit definition we use. Specifically, neural studies find no clear separation between neural substrates of model-free and model-based RL(Daw et al., 2011), contrasting with consistent findings that habitual and goal-directed systems are underpinned by distinct brain regions(Gremel & Costa, 2013). Additionally, habitual social media posting, operationalised both through self-report and posting frequency, is associated with perseveration and reduced reliance on model-free RL – which is not what we might expect if habits were underpinned by model-free RL(Anderson & Wood, 2023). Together, this evidence converges towards a view of all reinforcement learning as part of a goal-directed, value-maximising system, distinct from a perseverative habitual system. (page 8, lines 181-207)

The Discussion:

"Theoretical research defines model-free RL as a goal-directed system, interacting with a value-free habitual system which perseveres with previous behaviour regardless of changes in expected reward(Miller et al., 2019). Consistent with this definition, reliance on perseverative posting behaviour over model-free RL correlates across users with other converging signatures of habit, such as self-reported habitual social media use and disruption of posting in response to changing contexts(Anderson & Wood, 2023)." (page 22, lines 603-609)

R1.2
Model boundary effects: When looking at Figs 4 and 5, it is striking how many parameters land in the boundaries (e.g. Fig 4a means that most people are fully "habitual). This points to some convergence issues. The authors may consider using hierarchical or MAP methods to get more reliable parameter estimates (which may improve the individual-differences findings).

We thank R1 for prompting us to more explicitly justify our choice of model-fitting procedure within the paper. We chose, and preregistered, to use Akaike Information Criterion Weights (AICw, (Wagenmakers & Farrell, 2004)), a method of single-subject maximum likelihood estimation, for model fitting because this method incorporates minimal a priori assumptions about the distribution of the parameters. Of note, we would also like to highlight that we did not find flags for convergence issues when running our modelling code using the 'optim' function in R.

Hierarchical and MAP methods rely on prior assumptions about parameter estimates and distributions to increase the precision of single-subject parameter estimates. Specifically, hierarchical methods incorporate a priori assumptions about the group-level parameter distribution to estimate parameters at the individual level, while MAP methods incorporate a prior density over the estimated quantities. These model fitting approaches are useful in conventional modelling papers which are able to ground their a priori assumptions about parameter estimates by building on a wealth of prior work with similar models and experimental data. Importantly, the resulting parameter estimates will only be more reliable (i.e., externally valid) to the extent that such prior assumptions are correct.

In contrast to conventional modelling papers which estimate parameters from experimental data, ours is one of the first studies to fit computational model parameters directly to social media data (to our knowledge, previous examples include only (Camerer et al., 2024; da Silva Pinho et al., 2024; Lindström et al., 2021)), and it is the first to extract parameters specifically from Twitter data. Given the novelty of the field, we therefore selected a modelling approach which incorporated minimal a priori assumptions about parameter values. This empirical-first approach allows us to study how RL works in the real world without making assumptions that this will be similar to laboratory data. Indeed, we hope that publishing this and other similar papers will pave the way for future work to incorporate the uncovered empirical distributions into a priori assumptions for hierarchical or MAP methods.

However, we recognise that we did not originally justify this explicitly in the original manuscript. We have therefore added a justification of our choice of modelling procedure to the Methods:

"We fitted seven computational models to the dataset and established the winning model, representing the inferred underlying process, as that with the highest mean AICw(Wagenmakers & Farrell, 2004). AICw is a method of single-subject maximum likelihood estimation which, unlike other popular methods such as hierarchical or maximum a posteriori model fitting, incorporates minimal prior assumptions about the value or distribution of parameters. This was appropriate given the paucity of previous studies fitting computational models to social media data which could have been used to estimate plausible priors." (page 28, lines 791-798)

R1.3
I also did not see a parameter recovery matrix, but I might have missed it.

We included a parameter recovery matrix in the Supplementary Material (Figure S3, Figure R1), which we refer to in the main paper Methods and Discussion. We also added a further reference to parameter recovery in the Results:

> *"We validated using parameter recovery that fitted habit weight and action learning rate were not (anti-)correlated in simulated uncorrelated data, indicating that the relationships between posting latency and each of these parameters are independent features of the empirical data (Supplementary Material)."* (page 16, lines 565-569)

As Figure R1 shows, parameter recovery was very good for all parameters in all models except the Changing Policy model (for which we were not interested in exact parameter values). Specifically, fitted parameters were highly correlated with their simulated counterparts (on-diagonal), and showed close to 0 correlation with all other parameters (off-diagonal).

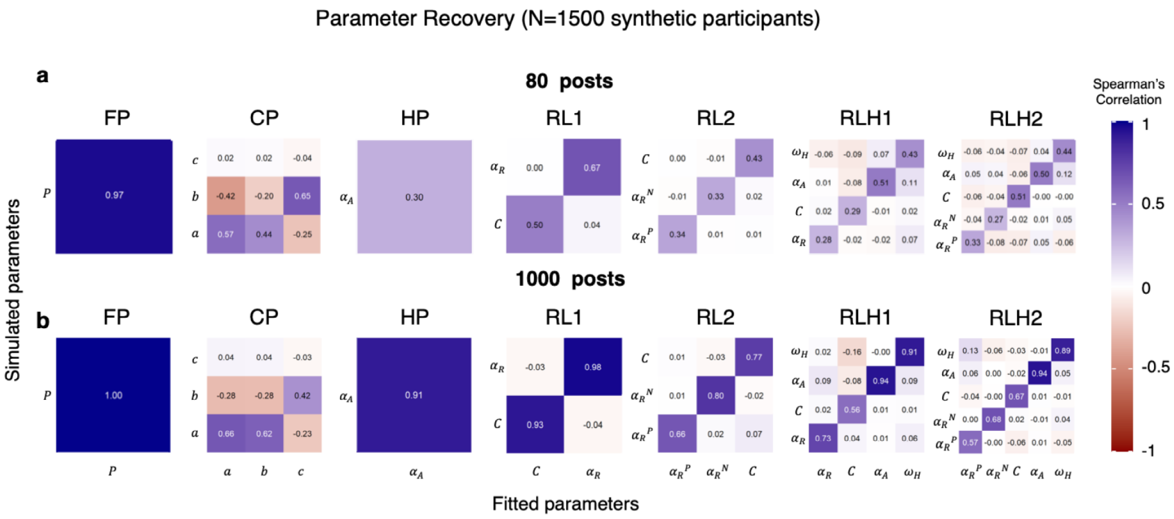

*"**Figure R1 (Figure S3). Parameter recovery.** Shaded colours represent the Spearman's correlation between the fitted and actual parameter, for each of the seven models. High Spearman's correlation indicates good parameter recovery. Low correlations for all nondiagonal squares also indicates that the modelling procedure can effectively distinguish between different parameters within the same model, and thus that each parameter represents a distinct aspect of the cognitive process. FP = Fixed Policy, CP = Changing Policy, HP = Habitual Policy, RL1 = Pure RL, single learning rate, RL2 = Pure RL, double learning rate, RLH1 = Hybrid RL-habit, single learning rate, RLH2 = Hybrid RL-habit, double learning rate. **a)** Parameter recovery for 80 posts. **b)** Parameter recovery for 1000 posts indicates very good recovery for all models apart from the CP model, for which we were not interested in exact parameter values."*

R1.4
It would be nice to have more descriptives of the sample, e.g. how many posts per person, how many likes per post, variability of posts, variability of posting latency, etc.

We have now enhanced Table 1 (Table R1) with these suggestions. Specifically, we added the following rows: 'Number of posts per person' (how many posts per person), Standard deviation of Likes per post within person (in addition to the existing Mean Likes per post within person; how many likes per post), Standard deviation of posting latency (days) (variability of posting latency). Additionally, we added the column: 'Standard Deviation' (variability of posts for the row 'number of posts per person').

Variable	Sample	Minimum	Maximum	Mean	Median	Standard deviation
Age	Discovery	15	82	36	35	14
	Confirmatory	13	80	36	35	14
Mean posting latency (days)	Discovery	0.04	16.21	2.10	1.92	1.35
	Confirmatory	0.07	21.03	2.12	1.86	1.42
Standard deviation of posting latency (days)	Discovery	0.06	110.82	3.98	3.00	6.02
	Confirmatory	0.13	231.84	4.76	2.94	11.25
	Discovery	0	40,485,990	38,143	390	1,200,326

Follower number	Confirmatory	0	17,240,910	32,638	350	607,397
Mean Likes per post within person	Discovery	0	11,495	16.77	1.18	350.78
	Confirmatory	0	32,239	37.18	1.03	885.43
Standard deviation of Likes per post within person	Discovery	0	19,075.57	36.48	2.21	632.69
	Confirmatory	0	24,036.64	55.11	1.97	961.69
Number of posts per person	Discovery	80	2,437	260.18	185.00	216.55
	Confirmatory	80	1,950	249.59	185.50	199.51

"Table R1 (Table 1). Descriptive statistics for the discovery sample and confirmatory sample."

R1.5
The model equations would be a bit more explained, esp eq. 3-5

We thank R1 for this suggestion and have added a more comprehensive explanation of how we developed the model equations.

For equations 3-5 (now the 'Habitual Policy' model after a suggestion by R3.11 below, formerly the Pure Habit model), we added the following explanation:

> *"Habitual Policy model (HP). The Habitual Policy model is based on a definition of 'habit' as a value-free process, following Thorndike's (1911) Law of Exercise to define habits: that an action that has been taken often in the past is likely to be repeated in the future(Miller et al., 2019; Thorndike, 1911). Unlike RL, which involves value-based learning, habits involve learning from past actions in a form of value-free learning, resulting in perseveration or repetition of previous actions.*
>
> *The habit model constructs a latent variable, H or 'habit tendency', an estimate of expected future action latency. H is learned using temporal difference learning with 'action prediction errors' δ_{H^t} (Equation 3). Specifically, in temporal difference learning, an action prediction error is calculated after each successive action is performed, and is the difference in latency between the action that has actually been taken, and the previous habit tendency. Theoretical and experimental research suggests that such a temporal difference learning mechanism underlies habit learning in the brain, with action prediction errors possibly encoded via dopamine(Bogacz, 2020; Greenstreet et al., 2025; Miller et al., 2019). At each step, H is then updated by combining the previous $H$with the action prediction error.*
>
> *The initial H is estimated from the data as the first posting latency in each user's dataset, but we confirmed that our model comparison results were robust to this approximation by repeating analyses with different initializations (Supplementary Material). The action learning rate α_A is a free parameter fitted for each participant which controls the relative influence on H of more recent actions compared with actions further in the past (Equation 4). H_t is then used as the policy (Equation 5).*
>
> *(Equation 3)* $\delta_{H^t} = \tau_{Post^t} - H_t$
>
> *(Equation 4)* $H_{t+1} \leftarrow H_t + \alpha_A * \delta_{H^t}$
>
> *(Equation 5)* $Policy_{t+1} = H_{t+1}$ " (page 32, lines 901-905)

For the RL model, we now added further explanation of the equations, and also explicitly point to the Supplementary Material which includes a detailed derivation of the model, as well as a figure explaining how the 'vigour cost constant' parameter impacts the policy (as suggested by R3.9):

> *"Single Learning Rate RL (RL1). The RL model aims to maximise the average reward rate; that is, the reward, minus costs, obtained per unit time*(Lindström et al., 2021; Niv et al., 2005, 2007)*. To do so, the model estimates the reward expected on the next post, $\hat{R}_{t+1}$, using temporal difference learning. The expected reward $\hat{R}_t$ is first compared with observed reward R_t to compute the reward prediction error, δ_{R^t}, which, like action prediction errors, is likely encoded via dopamine in the brain(Bogacz, 2020; Schultz et al., 1997) (Equation 6). This prediction error δ_{R^t} is then scaled by the reward learning rate α_R (a recency-weighting mechanism analogous to the action learning rate in the Habitual Policy model) and added to the previous expected reward to update the expectation, and result in a new expected reward, $\hat{R}_{t+1}$ (Equation 7). To compute $Policy_{t+1}$, the expected reward $\hat{R}_{t+1}$ is then combined with the subjective*

vigour cost constant C (Equation 8). Specifically, the policy is inversely proportionate to the expected reward, such that higher expected rewards produce shorter posting latencies (Equation 8).

The vigour cost constant C associates each posting action with a 'vigour cost', where the vigour cost of action τ_{Post^t} is equal to $\frac{C}{\tau_{Post^t}}$, and thus is higher for shorter posting latencies(Niv, 2007; Niv et al., 2007). If there were no (subjective) costs associated with posting, there would be no reason not to simply try to post infinitely fast, regardless of reward. However, given that each action (i.e., each post) is associated with a subjective cost, it is only worth increasing posting frequency if the corresponding expected reward from doing so justifies the increased cost.

In the real world, the 'cost' of posting might correspond to any factor which deters an individual from posting fast – such as the increased cognitive effort as well as the opportunity cost of time associated with posting more quickly instead of engaging in other activities. The 'vigour cost' of posting specifically denotes any effort and time which is greater for each post at a higher frequency than for each post at a at lower frequency – as the vigour cost per post, $\frac{C}{\tau_{Post^t}}$, is greater for smaller τ_{Post^t}. For example, posting very fast on Twitter might be more costly relative to posting slowly because posting fast requires one to actively search for new material to post about rather than simply posting whenever news comes up, as well as to post more creatively to ensure less overlap with multiple other recent posts. A figure showing how varying the vigour cost constant C affects the relationship between expected reward and policy, is contained in the Supplementary Material.

The initial values for both $Policy$ and expected reward $\hat{R}$ were estimated from the data, and we confirmed that our main model comparison results were robust to different initialization decisions by repeating model comparison with both $Policy$ and $\hat{R}$ initialized using a variety of different values (Supplementary Material). In the main analyses, $Policy$ is initialized as C divided by the mean value of Likes across all posts for that user, while $\hat{R}$ is initialized as the number of rewards on the first post. The free parameters for each user are the reward learning rate, α_R, and the vigour cost constant C.

(Equation 6) $\delta_{R^t} = R_t - \hat{R}_t$

(Equation 7) $\hat{R}_{t+1} \leftarrow \hat{R}_t + \alpha_R * \delta_{R^t}$

(Equation 8) $Policy_{t+1} = \frac{C}{\hat{R}_{t+1}}$" (page 32, lines 909-956)

R1.6

Did the authors consider alternative policy distributions other than just an (inverse) exponential, e.g. Poisson distribution?

We thank R1 for the opportunity to more stringently test the robustness of our modelling results to different analytic decisions, which we believe has strengthened our findings. The constraints for a policy distribution were that it should (1) allow a continuous outcome variable (as posting latency is continuous), (2) only allow positive outcomes (as posting latency cannot be below 0), and (3) be parameterised such that the expected value of the policy distribution is the current policy.

A Poisson distribution did not satisfy (1) as it does not have continuous outcomes. However, we repeated the model fitting with two other distributions; gamma and normal distributions. We found that model comparison was robust to both these distributions. We have added a new section in the Supplementary Material, 'Model comparison with different policy distributions', which describes this sensitivity analysis:

"

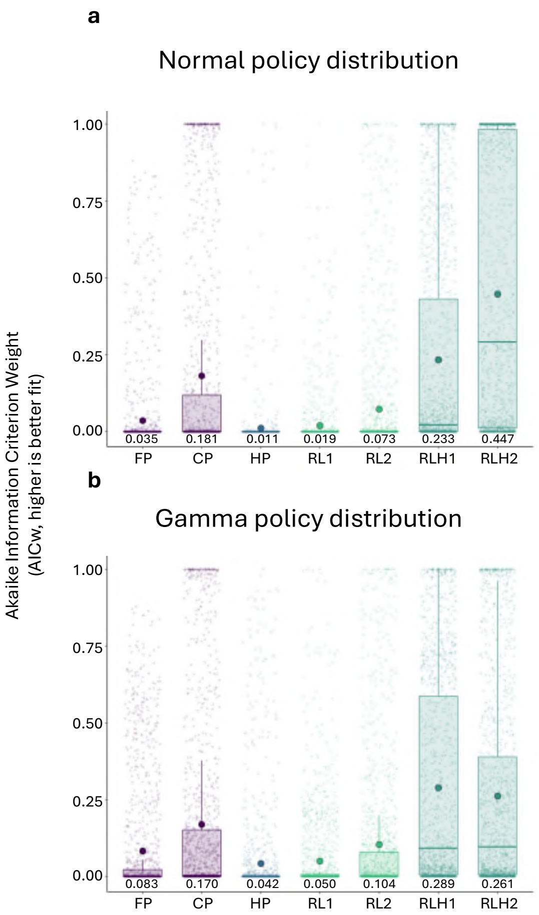

Figure R2 (Figure S8). Model comparison with different policy distributions in the confirmatory sample. *Model comparison did not differ substantially for a normal or gamma distribution, compared with the exponential distribution used in all analyses in the main paper. Specifically, hybrid RL-habit models still provided the best fit with both these variations. Higher AICw represents a better fit. Outlined circles represent the mean AICw, which is also indicated as numbers along the top of the x-axis (the criterion for which best fitting model was selected), while box plots represent the median and interquartile range for AICw, and scattered points represent the AICw for each individual user.* ***a)*** *Model comparison results for models fit with a normal policy distribution.* ***b)*** *Model comparison results for models fit with a gamma policy distribution.*

In our main analyses, we assumed that each posting latency, τ_{Post^t}*, was a random draw from an exponential distribution with expected value* $Policy_t$ *(Equation S9, which is the same as Equation 1 in the main paper):*

Equation (S9) $$\tau_{Post^t} \sim Exponential\left(\frac{1}{Policy_t}\right)$$

To test the implications of this assumption, we repeated the model fitting and model comparison with two alternative policy distributions. The constraints for the distribution were that (1) the distribution must allow a continuous outcome variable (as posting latency is continuous), (2) τ_{Post^t} *must be greater than 0, and (3) the expected value of the distribution would be* $Policy_t$*. First, we used a normal distribution with both mean (expected value) and standard deviation* $Policy_t$*, and for every* τ_{Post^t} *resampled from the distribution until* τ_{Post^t} *was greater than 0 (Equation S10).*

Equation (S10) $$\tau_{Post^t} \sim Normal\left(Policy_t\,,\; {Policy_t}^2\right)$$

Next, we used a gamma distribution with both 'shape' and 'scale' parameters set to $\sqrt{Policy_t}$ *(as the expected value of a gamma distribution is its shape parameter multiplied by its scale parameter) (Equation S11).*

Equation (S11) $$\tau_{Post^t} \sim Gamma\left(\sqrt{Policy_t}\;,\; \sqrt{Policy_t}\;\right)$$

Model comparison revealed that RL-habit hybrid models remained the winning models for both these policy distributions, suggesting that our main results do not depend on the specific policy distribution chosen (Figure S8)." (page 25, lines 527-566)

We additionally added a reference to this sensitivity analysis in the main paper:

"However, main model comparison results are not dependent on the specific policy distribution used, with both normal and gamma policy distributions yielding similar results (Supplementary Material)." (page 30, lines 856-859)

R1.7
It would be nice to have an indication for significances in the Figures. I believe that Fig 1b is not essential.

We thank R1 for this suggestion, and have added significance to all figures (see Figure R3 for an example). We have retained Figure 1b because we believe it is helpful to readers from the social media discipline who have less experience with computational models or reinforcement leaning.

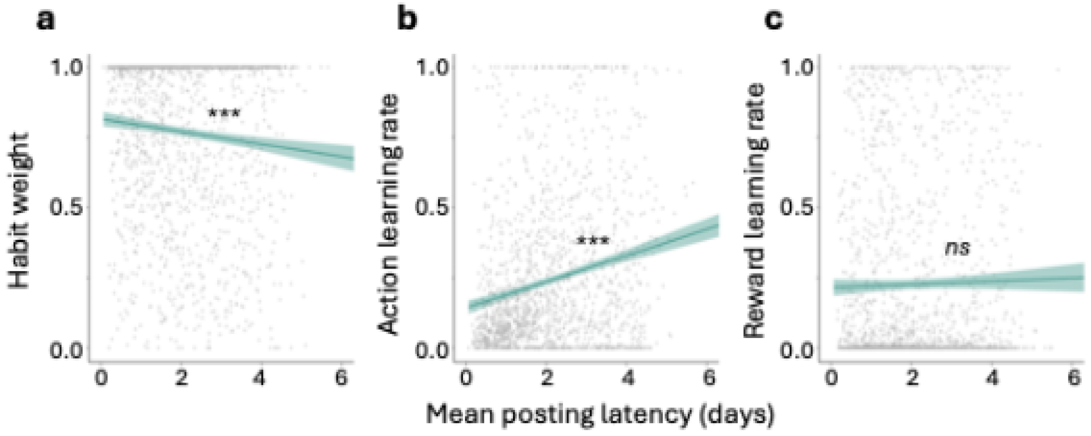

"Figure R3 (Figure 4). Relationship between posting latency and RLH1 parameters suggestive of habitual behaviour. *Scatter plots show the relationship between posting latency and an RLH1 parameter for each user. Plotted lines are lines of best fit, shaded areas represent standard error. Annotations indicate significance, according to the following scheme: ns = not significant, * = p<0.05, ** = p<0.01, *** = p<0.001.* ***a)*** *Posting latency negatively predicted habit weight (β = -0.022, p < 0.001).* ***b)*** *Posting latency positively predicted action learning rate (β = 0.047, p < 0.001).* ***c)*** *Posting latency did not significantly predict reward learning rate (β = 0.006, p = 0.343)."*

R1.8
There seems a typo for the replication sample N: Intro: N=1558, Results: N=1158

We thank R1 for pointing this out and have corrected the typo.

R1.9
I could not find any data availability statement

The data is not available to be shared due to privacy and ethical considerations. We have now explained this in a data availability statement:

"The data for this study cannot be shared because of privacy considerations. Source data are available at [link]." (page 37, lines 1061-1062)

However, code across the entire analysis pipeline from cleaning the dataset, to running and fitting the models, to computing statistics and plotting figures is freely available online on Github and the Open Science Framework. We have now also added a code availability statement:

> *"The code used for all analyses are freely available online, on Github*(Georgiaturner/Rl_twitter, n.d.) *and Open Science Framework at https://osf.io/fmuks/files/ca705561-ebc4-45a1-8ac1-d4b1e9fde007 [link]."* (page 37, lines 1065-1067)

Overall, we thank R1 for their review which gave us the opportunity to explain and justify more of our decisions in more detail, as well as conduct important analyses to ensure robustness, greatly improving the communication and validation of our results.

Reviewer #2 (Remarks to the Author):

Review: "A computational model of reward learning and habits on social media"

In this paper, the authors propose a computational model that combines reward learning and habit-like responding and compare this model to a set of alternative models in two Twitter data sets. The results indicate that Twitter users are both sensitive to rewards and tend to repeat previous actions (response latencies). They also provide evidence for some differences between men and women, and younger versus older users in the weight of the two processes. There is a lot to like about this paper: it tackles important questions and uses rigorous modeling methods, combined with model-independent analysis of reward signatures. Overall, this is very good work, which will interest a broad readership.

We thank R2 for their positive evaluation of our work.

However, I have several concerns and questions that merit clarifications or additional analysis (none of which are grounds for rejection).

R2.1
Model formulation. The authors derive their model from an earlier model developed by Niv. Specifically, they base it on the equations linking the optimal response rate to the average reward rate (which depends on expected immediate rewards, the number of responses required, costs etc) in a ratio schedule. They make a number of simplifying assumptions and end up with a model where the response latency is inversely proportional to the expected reward and directly proportional to a "cost" parameter. While this is motivated by Niv's theory, it results in a model that only cares about the average reward but is insensitive to time. This makes sense in the context of a ratio schedule but falls short in other cases. For example, consider the case where the likes a Twitter user receives depend on their posting latencies (e.g., R = 20 if 10 < latency < 20 and R = 1 otherwise, or some smoothed version thereof), I don't see how the authors model could not learn this optimal latency. The only way for it's policy to fall in the optimal range is by adjusting the cost parameter (and the initial reward), which does not seem satisfactory. The same issue would presumably arise on interval schedules. While I do not think this is a critical flaw of the paper, I do think it requires a careful description of the limits of the model, and acknowledging that even though it seems to describe user behavior quite well, it might fall short as a more complete account social media posting (I assume that humans can easily learn such things, and probably often do).

R2 is correct that our model does not explicitly calculate the dependence of R on latency, and that for this and other reasons, it does not provide a complete account of social media posting. We have therefore added a clearer discussion of this limitation to the Discussion:

> *"It is important to note, however, that as an initial step modelling multiple cognitive processes in real-world social media behaviour, both our model and dataset are simplifications of a complex process. We document the specific simplifying assumptions of our model in the Supplementary Material. For example, our model considers only two generative processes underlying posting – the habitual system and model-free RL, whereas in reality there are likely to be many more interactive cognitive processes governing posting – for example, model-based RL(Turner et al., 2024) and social learning(Brady et al., 2021). Further, our model assumes that rewards are delivered on a ratio schedule, such that the amount of Likes received does not depend on posting latency. This is unlikely to be the case in the real world, where it is possible that Likes do depend to some extent on latency (for example, because more frequent posting increases visibility via the algorithm, or because too frequent posting decreases the likelihood that followers will Like every post), and that social media users actively learn a relationship between posting latency and Likes received. Furthermore, our model assumes that all 'actions' (i.e. posts) are the same, whereas in reality, social media users may consider posts with different content as separate 'actions' which each have different reward schedules."* (page 25, lines 692-707)

However, we would also like to highlight that although the model does not explicitly learn a relationship between R and posting latency, the model is not fully insensitive to time. We note that because the effort cost increases for shorter latencies (due to $\frac{C}{\tau_{Post^t}}$, the vigour cost), the model will only decrease latency if a corresponding increase

in reward per unit time can be obtained. Therefore, when the highest reward per unit time decreases for shorter latencies, the model will converge towards a higher latency.

To illustrate this, we simulated the single learning rate RL model (RL1) with ten reward schedules which differed only in the posting latency for which the highest reward per post would be received (Figure R4a). Specifically, each reward schedule was defined using the following equation, where $R(\tau_{Post})$ is the Likes received for posting latency τ_{Post}, and $\tau_{Post_{max}}$ is the posting latency for which the reward received is the highest.

$$R(\tau_{Post}) = 3 * \ e^{-0.2 * \tau_{Post} - \tau_{Post_{max}}}$$

Here, 3 represents the maximum reward received and 0.2 represents the decay rate (λ) of the exponential. We simulated ten reward schedules where $\tau_{Post_{max}}$ took values of 3, 5, 7, 9, 11, 13, 15, 17, 19 and 21. In each case, the RL1 model was simulated with a reward learning rate parameter of 0.2 and a vigour cost constant parameter of 0.4. Figure R4a shows how in each reward schedule, the reward received for each posting latency decreases exponentially on either side of $\tau_{Post_{max}}$.

For each reward schedule, we simulated the RL1 model 1000 times, each for 5000 posts, to establish the 'policy at convergence', which we took to be the median policy at post number 5000 across each of the 1000 simulations. Figure R4b shows how the policy at convergence varies for each of the ten different values of $\tau_{Post_{max}}$. This shows that when the latency corresponding to the maximal external reward is higher, the model policy converges to a higher latency. We note that the optimal posting latency (which is represented by the policy at convergence) does not correspond to the maximal-external-reward posting latency, because as R2 rightly notes, the optimal posting latency does not maximise external reward per post, but instead maximises subjective reward rate (i.e. taking account of factors such as vigour costs which increase for lower posting latencies). In this way, the model is not fully insensitive to time, as it is able to adjust policy in the right direction when reward per unit time depends on latency (i.e., increasing policy to be relatively higher when reward rate is relatively higher for longer latencies).

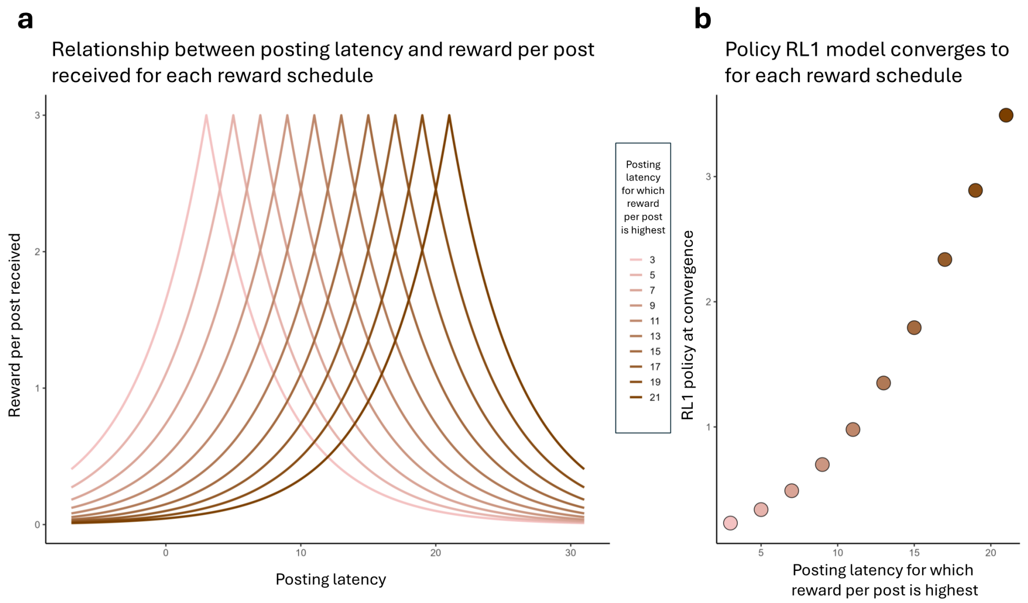

Figure R4. RL policy at convergence is sensitive to the optimal posting latency. a) *Each of the ten simulated reward schedules differed only in the posting latency for which the highest reward per post was received. In each case, reward per post decreased exponentially on either side of this maximal-external-reward posting latency.* ***b)*** *The RL1 policy converged to higher posting latencies for higher maximal-external-reward posting latency, indicating that it is not fully insensitive to time.*

Finally, we conducted an additional model comparison analysis comparing each of our models to the previous RL model of (Lindström et al., 2021), which did not make the simplifying 'ratio schedule' assumption that our model made. We found that both our RL-habit hybrid models (RLH1 and RLH2) provided better fits to our confirmatory sample than the RL model of Lindström et al, despite the ratio-schedule assumption. We have now added an additional section in the Supplementary Material ('Model comparison with the RL model of Lindström et al. (2021)) detailing this analysis:

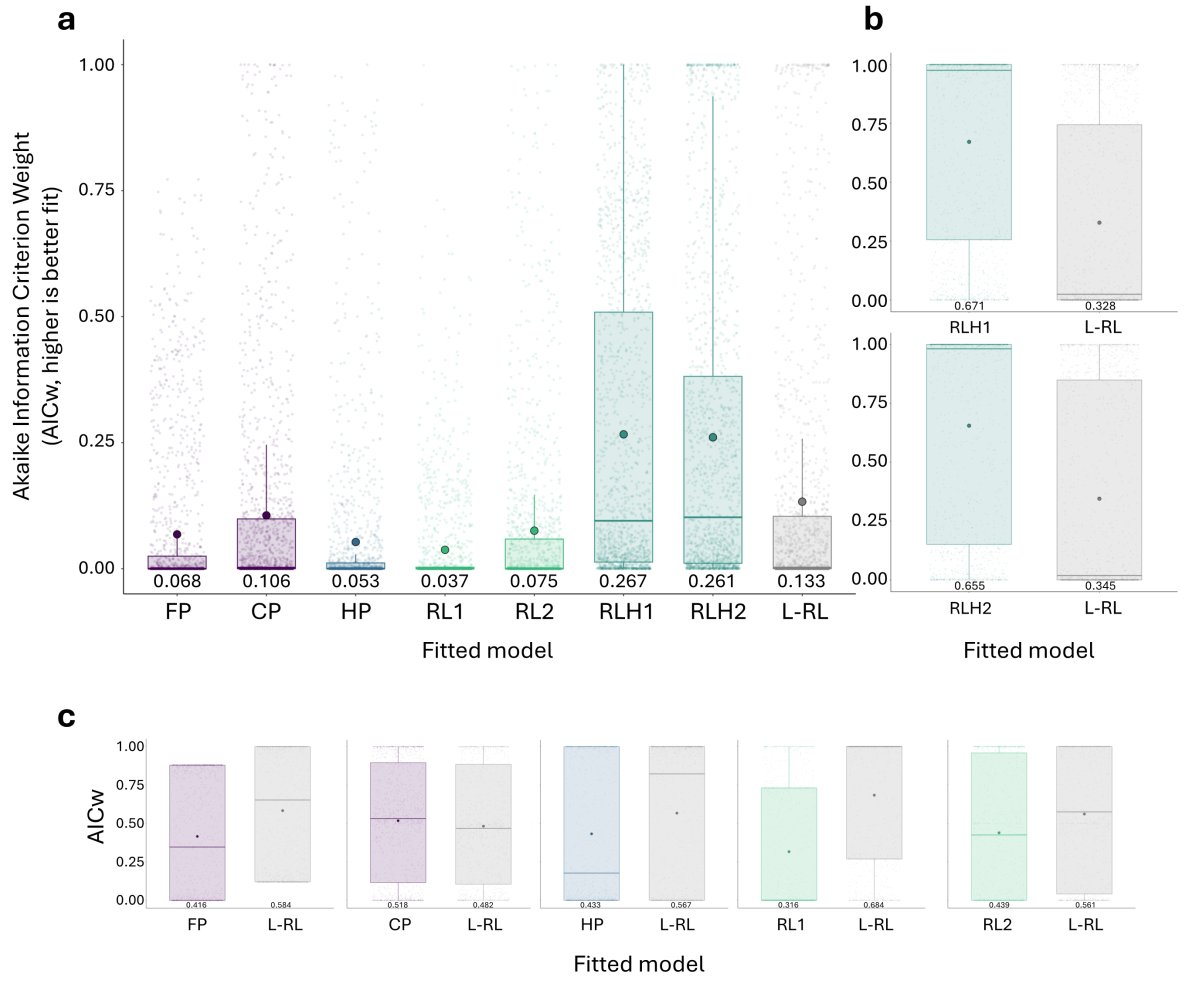

Figure R5 (Figure S10). Model comparison with the Lindström RL model. *FP = Fixed Policy, CP = Changing Policy, HP = Habitual Policy, RL1 = Pure RL, single learning rate, RL2 = Pure RL, double learning rate, RLH1 = Hybrid RL-habit, single learning rate, RLH2 = Hybrid RL-habit, double learning rate, L-RL = Lindström RL model. The RLH1 and RLH2 models provided better fits to the confirmatory sample than the Lindström RL model. Higher AICw represents a better fit. Outlined circles represent the mean AICw, which is also indicated as numbers along the top of the x-axis (the criterion for which best fitting model was selected), while box plots represent the median and interquartile range for AICw, and scattered points represent the AICw for each individual user.* ***a)*** *Model comparison results for all seven models from the main paper along with the Lindström RL model.* ***b)*** *Model comparison results for the RL-habit hybrid models each compared with the Lindström RL model, showing both of the RL-habit hybrid models provide a better fit (higher AICw).* ***c)*** *Model comparison for all other models, each compared with the Lindström RL model.*

Our modelling procedure was inspired by Lindström et al. (2021)(Lindström et al., 2021)*, who were the first, to our knowledge, to adapt a model from the cross-species literature to describe a reinforcement learning process underlying posting on social media. Although inspired by the same animal model of action speed(Niv et al., 2005, 2007), our model differs from the Lindström RL model (see 'Model derivation' above for a complete description and justification).*

To establish whether our winning model provided a better account of the cognitive processes underlying behaviour than the Lindström RL model, we fitted the Lindström RL model to our confirmatory Twitter dataset and compared its fit to all other models both together (Figure S10a) and individually (Figure S10b,c). Given that AICw identifies the best fitting model given the data and the set of candidate models, the comparison with each model individually allowed us to directly compare the performance of the Lindström model with each of our models in turn.

We first found that the Lindström RL model provided a better fit to our Twitter dataset than the Fixed Policy (FP) model. Given that the FP model is equivalent to the 'No Learning' model in Lindstrom et al. (2021)(Lindström et al., 2021)*, this result replicates the main finding of Lindström et al. that their RL model provides a better fit than a FP model, in a new social media dataset (generalising to Twitter from*

Instagram and forums) (Figure S10b). Importantly however, the Lindström RL model provided a worse fit to the data than both our RL-habit hybrid models (RLH1 and RLH2) (Figure S10c)."

R2.2
Description of habit component. The authors motivate their habit model based on the "habits without values", and refer to a "stickiness" parameter. While I do get what the authors mean, the "stickiness" parameter just determines the memory of a moving average of previous posting latencies – i.e calling it stickiness seems a bit odd. Although I am open to being convinced differently.

R2 makes a good point that 'stickiness' may not be the best way to describe the parameter. We have therefore changed the name of the parameter to 'habit weight', in all parts of the manuscript (including figures). As well as being consistent with R2's terminology (R2.7), 'habit weight' better captures the key idea that this weight determines the relative weighting of the habit compared with the goal-directed system.

R.2.3
Relatedly, the Miller model bases habits on frequencies of different actions (e.g., action A 10 times, action B 100 times). As described, the authors' model formulation simplifies this a lot with the moving average formulation, which just says that posting latencies will be similar to previous posting latencies. Alternatively, one could count the number of posts in various time bins (corresponding to actions), which would determine the habit strength. Although I guess this in practice would be quite similar. In summary, I ask the authors to be a bit more transparent in how their model and choices correspond to the motivating literature. No need to oversell.

Our habit model takes key theoretical concepts from Miller's model, and applies these to the continuous space of action latencies. Specifically, our model assumes, like Miller's model, that habitual actions are (1) similar to actions repeated in the past, and (2) learned through temporal difference learning with 'action prediction errors'. This hypothesised temporal difference learning mechanism common across both habit and reward learning, has been supported both by theoretical (Bogacz, 2020) and experimental research (Greenstreet et al., 2025).

We therefore chose a value-free learning mechanism for habits, which we directly adapted to the action latency space such that the continuous latent variable learned denoted the action latency rather than the probability of performing a given action. However, as R2 points out, in practice this would be similar to alternative mechanisms of value-free learning such as that suggested by R2.

We thank R2 for the opportunity to more precisely explain and refer to the literature in which our habit model is grounded, and have added a more comprehensive description to the Methods section:

> *"The habit model constructs a latent variable, H or 'habit tendency', an estimate of expected future action latency. H is learned using temporal difference learning with 'action prediction errors' δ_{H^t} (Equation 3). Specifically, in temporal difference learning, an action prediction error is calculated after each successive action is performed, and is the difference in latency between the action that has actually been taken, and the previous habit tendency. Theoretical and experimental research suggests that such a temporal difference learning mechanism underlies habit learning in the brain, with action prediction errors possibly encoded via dopamine(Bogacz, 2020; Greenstreet et al., 2025; Miller et al., 2019). At each step, H is then updated by combining the previous H with the action prediction error.*
>
> *The initial H is estimated from the data as the first posting latency in each user's dataset, but we confirmed that our model comparison results were robust to this assumption by repeating analyses with different initializations (Supplementary Material). The action learning rate α_A is a free parameter fit for each participant which controls the relative influence on H of more recent actions compared with actions further in the past (Equation 4). H_t is then used as the policy (Equation 5)."* (page 34, lines 884-899)

R.2.4
Model falsification. I like this part a lot. A couple of questions: how sensitive are the results to the exact definition of the reward signature (e.g., the number of trials considered for the PE)?

We appreciate R2's positive evaluation of the model falsification analysis. To show that the model falsification is robust to changing numbers of trials which contribute to the inferred reward prediction error, we repeated the analysis with two variations in numbers of trials: (1) all previous trials considered (rather than just the previous ten), and (2) just the previous five trials considered. We have added these to a new section in the Supplementary Material ('Model falsification with different definitions of reward prediction error'), and added a reference to this section in the main paper as well.

In the Supplementary Material:

> *"In the model falsification analysis in the main paper, we examined a model-agnostic statistical signature of reward learning by inferring the reward prediction error directly from the data. Specifically, we*

calculated reward prediction error as the difference between the Likes received on the previous post, and the mean number of Likes on the preceding ten posts before that (the 'prediction').

This is an approximation to the true reward prediction error, as the amount of previous rewards experienced which contributes to each reward prediction would differ for each user depending on their individual reward learning rate. Therefore, to establish whether the model falsification results were dependent on the specific form of this approximation, we repeated the model falsification analysis with two alternative definitions of reward prediction: the mean number of Likes on all preceding posts for that user, and the mean number of Likes on the five preceding posts for that user. The model falsification results did not differ substantially for each of these alternatives, confirming that they are robust to these different formulations of the reward prediction approximation (Figure S11).

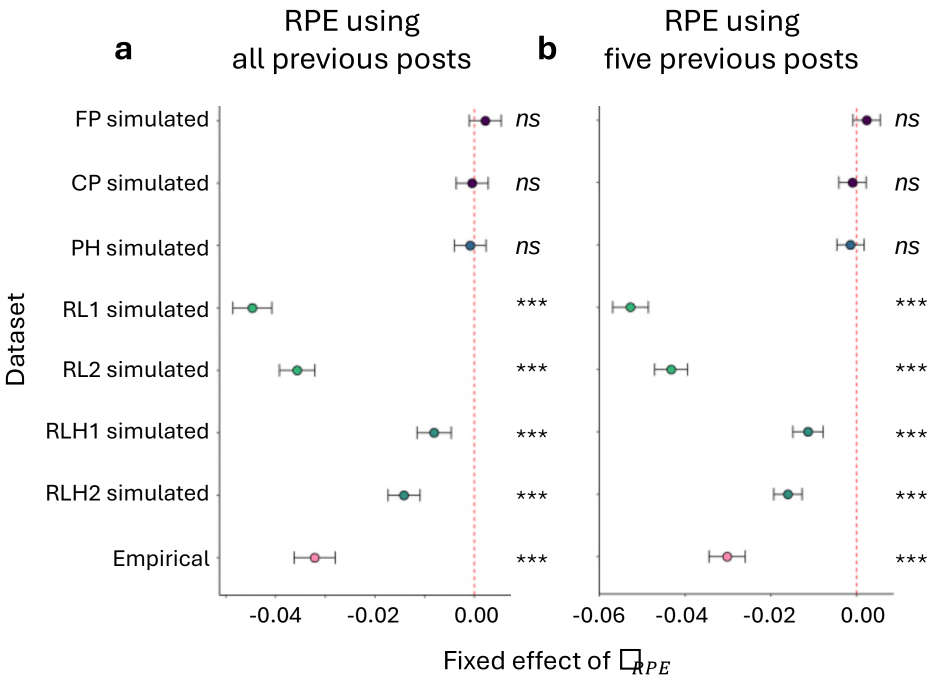

Figure R6 (Figure S11). Model falsification with different definitions of reward prediction error (RPE)*. Dots show estimates of β_{RPE}, error bars show 95% confidence intervals. FP = Fixed Policy, CP = Changing Policy, HP = Habitual Policy, RL1 = Pure RL, single learning rate, RL2 = Pure RL, double learning rate, RLH1 = Hybrid RL-habit, single learning rate, RLH2 = Hybrid RL-habit, double learning rate. Model falsification results were not affected by varying the amount of trials considered for the inferred RPE. Specifically, the empirical dataset also shows a significantly negative fixed effect of β_{RPE}, which falsifies the hypothesis that the FP, CP or HP models underlie the data.* ***a)*** *Model falsification where RPE included all previous posts for each reward prediction.* ***b)*** *Model falsification where RPE included only the five previous posts for each reward prediction."* (page 31, lines 649-677)

In the main paper:

"We note that this is an imperfect estimate of reward prediction, as the model's actual reward prediction is learned through RL and depends on an individual's reward learning rate. However the approximate nature of the reward prediction estimate does not affect results: the model falsification result is robust to different ways of estimating reward prediction, both using all preceding posts and using the five preceding posts (Supplementary Material)." (page 35, lines 1016-1021)

R2.5
The size of the fixed effects estimate in the data (-.031) seems to be more similar to the pure RL models than the RL + Habit models (-0.012 & -0.016). Could you discuss that?

As R2 suggests, the size of the fixed effects estimates suggests that the overall contribution of an RL process in the empirical dataset is underestimated by the RLH1 model simulated with the best fitting parameters across the dataset. A possible reason for this is that we simulated the RLH1 dataset using the RLH1 model, the winning model across users. However, several subjects were better fit by pure RL models: out of 1,558 users, 57 were best fit by RL1 and 132 were best fit by RL2. Therefore, simulating these subjects with the RLH1 model is likely

to have underestimated the RL component in their data. An additional potential explanation is the fact that, as discussed above (R2.1), our simplified RL model only captures ratio-schedule model-free RL, whereas there is likely to in reality be a more complex RL process underlying social media posting (such as model-free RL which takes account of interval schedules, model-based RL, etc).

We have now added an explicit discussion of this point in the main text:

> *"We also note that the size of the fixed effects estimate in the data seems to be in between the pure RL and RL-habit models, rather than the same as the RL-habit models as we might expect if the RL-habit models fully capture all RL in the data. This suggests that the RL-habit models underestimate the strength of RL in the data, consistent with the possibility that our simplified model (for example, only capturing model-free RL on a ratio schedule) does not fully capture the more complex RL process underlying social media posting (which could include, for example, interval-schedule model-free RL, model-based RL, or other RL systems)."* (page 15, lines 392-399)

R2.6

Finally, and most importantly: are there any model-independent signatures of "habitual" responding? Can you distinguish between pure RL and RL + Habit based on such a signature? It might be difficult, but would be a strong addition. However, not crucial for the success of the paper.

We thank the reviewer for this suggestion, which has prompted us to develop and validate a model-agnostic signature of habit, which we believe has greatly improved our ability to demonstrate that our model and model fitting procedure are mechanistically related to the cognitive constructs we are interested in.

Specifically, the habit component of the model represents a tendency to produce posting latencies that are similar to those in the (recent) past, irrespective of changes in expected reward. We therefore calculated a statistical signature of this process as the extent to which previous posting latencies predict future posting latencies even after controlling for similarities in expected reward at the two different timepoints. After computing this statistical signature, which we label β_{AUTO} (referring to 'autocorrelation' across posting latency policies), we found that its fixed effect was similar to the RLH1 model (Figure R6a), and also correlated positively with both the simulated habit weight in the RLH1 simulated dataset (Figure R6b) and the fitted habit weight in the empirical dataset (Figure R6c). These results confirm that the statistical signature of habit relates to the contribution of the habit system across simulated users (Figure R6b) and the recovered contribution of the habit system across users via fitted habit weight (Figure R6c).

We added a new section to the Supplementary Material ('Statistical signature of habit') explaining and displaying this new analysis:

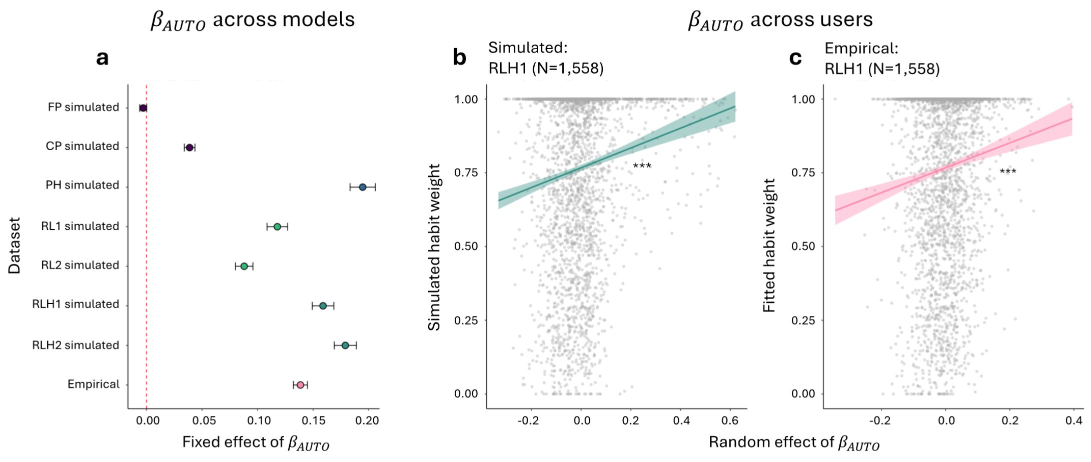

"Figure R7 (Figure S6). The statistical signature of habit, β_{AUTO}, across models and users. a) *Dots show estimates of β_{AUTO}, error bars show 95% confidence intervals. A visual representation of the fixed effects of β_{AUTO} in the simulated and empirical datasets. The pure RL models appear to have smaller fixed effects of β_{AUTO}, but are not 0 due to the approximated nature of the reward prediction being controlled for.* ***b)*** *Scatter graphs show the random effects of β_{AUTO} for each user predicting simulated or fitted habit weight. Lines of best fit are shown, with shaded regions representing standard error. Annotations indicate significance, according to the following scheme: ns = not significant, * = p<0.05, ** = p<0.01, *** = p<0.001. The random effect of β_{AUTO} in the RLH1 simulated dataset,*

simulated with the parameters of the confirmatory sample, positively predicts the simulated RLH1 habit weight across users (β = 0.336, p < 0.001). ***c)*** *The random effect of β_{AUTO} in the confirmatory sample positively predicts fitted RLH1 habit weight (β = 0.42, p < 0.001).*

To verify that the habit component of the RLH1 model was also working mechanistically as expected, we developed a statistical signature of habitual behaviour. Specifically, habit is distinguished by the tendency to repeat previous actions regardless of predicted reward. In contrast, in pure RL models RL1 and RL2, posting latency is fully determined by expected reward, therefore there should be no autocorrelation across successive posts after controlling for expected reward.

To quantify this statistical signature of habit, we used multiple linear regression to extract the coefficient β_{AUTO} denoting the extent to which posting latencies are autocorrelated across time over and above expected reward. Specifically, β_{AUTO} represents the predictive relationship between the posting latency at timepoint $t-1$, which we denote as $\tau_{Post^{t-1}}$, and the posting latency at timepoint t, $\tau_{Post^{t}}$, controlling for the predicted reward at timepoint $t-1$, $\hat{R}_{t-1}$. Because the extent to which successive posts are correlated across time over and above expected reward is a signature of habit, within the dataset simulated by the RLH1 model, we expected users with a higher habit weight to exhibit the habit signature more strongly, as indexed by a higher random effect of β_{AUTO}. Additionally, we also expected the fitted habit weight in the empirical dataset to be positively related to the random effect of β_{AUTO}.

To pre-process the variables, we first log-transformed $\tau_{Post^{t}}$ and $\tau_{Post^{t-1}}$ (because the distribution of posting latency was highly skewed) and then scaled them within users (because we were interested in within-individual relationships). We calculated $\hat{R}_{t-1}$ as the mean reward (number of Likes) on the ten preceding posts ($t-2$ to $t-11$), consistent with the inferred predicted reward we used in the statistical signature of reward learning. Importantly, the true reward prediction is an internal latent variable calculated by the model, for which the relative contribution of more recent vs. less recent posts is determined by each user's individual reward learning rate. Therefore, including only the previous ten posts, and weighting them all equally, is an imperfect estimate of the model's reward prediction. Because this is imperfect, our linear regression underestimates the shared variance between successive posts which can be attributed to predicted reward, and therefore overestimates the non-reward related autocorrelation for pure RL models. We therefore did not expect $\beta_{AUTO}$to be 0 even for pure RL models. We inputted these variables into a Multilevel Model (MLM), to relate $\tau_{Post^{t}}$ to $\tau_{Post^{t-1}}$ controlling for $\hat{R}_{t-1}$. Users were random effects, with random slopes for both predictors ($\tau_{Post^{t-1}}$ and $\hat{R}_{t-1}$).

First, we visualised the fixed effect of β_{AUTO} in datasets simulated by different models (the same datasets as those used for the statistical signature of reward learning analysis) (Figure S6a). As expected, β_{AUTO} was greater than 0 even for the pure RL models because of the approximated nature of the estimate of reward prediction $\hat{R}_{t-1}$. Next, to confirm that the habit component of our model functioned mechanistically as we expected, we examined the habit signature across users. We extracted the random effect of β_{AUTO} for each individual user, and examined how this habit signature predicted the value of the simulated habit weight parameter in the generated RLH1 dataset (the winning model in the AH confirmatory and dataset) (Figure S6b), and the fitted habit weight parameter in the empirical confirmatory sample (Figure S6c). β_{AUTO} significantly predicted simulated habit weight in the simulated RLH1 dataset (β = 0.414 [SE = 0.085], p < 0.001) and fitted habit weight in the empirical dataset (β = 0.414 [SE = 0.085], p < 0.001). This confirms that the simulated and fitted habit weight values are related to the extent to which individuals exhibit reward-independent action perseverance, a signature of habitual tendency." (page 20, lines 532-596)

We also refer to this analysis in the main paper:

"As well as reward learning, we developed a statistical signature of habit learning in which we related the simulated and fitted habit weight across users to autocorrelation across time, a signature of the perseverative nature of habits. Details of this model validation analysis are contained in the Supplementary Material." (page 20, lines 434-498)

R2.7
Habitual users. The authors note that users with more posts tend to be fit with a higher habit weight. It would be good to confirm that this is not an artifact of the fitting procedure, e.g., by simulating and fitting data based on the same parameter values, but with different number of posts. The question would then be if more posts => higher habit weight.

We thank R2 for pointing out that it is possible that the fitting procedure could produce a higher habit weight for users with more posts. To establish whether this was possible with the fitting procedure that we used, we followed R2's suggestion and simulated a dataset using the RLH1 model. So that the simulated dataset would

maximally reflect the empirical confirmatory dataset, we simulated N=1,558 agents each with the numbers of posts and of Likes on each post as each of the subjects in the AH confirmatory dataset.

We simulated every agent with the same set of parameters, each of which was the mean fitted parameter value for when the RLH1 model was fitted to the confirmatory dataset. These values were a reward learning rate of 0.23, a vigour cost constant of 4.68, an action learning rate of 0.24 and a habit weight of 0.77. We then fitted the RLH1 model back to this simulated dataset, and examined, for every parameter (i.e., habit weight as well as action learning rate, reward learning rate and vigour cost constant), whether post number was significantly related to fitted parameter value.

By running linear models where number of posts predicted each parameter, we found that number of posts per user indeed significantly predicted fitted parameter value for habit weight (β = 0.000163 [SE = 0.0000200], $p < 0.001$) and action learning rate (β = -0.0000448 [SE = 0.0000155], $p = 0.004$). Number of posts per user was not significantly related to the remaining two parameters (reward learning rate: β = 0.000361 [SE = 0.0000192], $p = 0.060$; vigour cost constant: β = -0.00326 [SE = 0.00240], $p = 0.175$).

As R2 highlights, this means that if we do not control for differences in number of posts, part of the relationship between posting frequency and habit could be explained by individual variation in number of posts. We therefore repeated all our main analyses where parameters were compared between individuals, now controlling for number of posts. All results remained significant even when controlling for number of posts, indicating that the relationship between frequency and RLH1 parameters was not an artifact of the fitting procedure. We now report this added control in each of the Results sections in which parameters are compared across individuals (in both the Main and Supplementary), as well as in the Methods. Further, we explain the control in the Preregistration Deviations table in the Supplementary Material.

We added the control to the results in the section where we relate posting latency to habit weight, action learning rate and reward learning rate:

> *"Sensitivity analyses confirm that these findings remain significant after removing posting latency outliers (Supplementary Material) as well as after controlling for number of posts per user (see Preregistration Deviations in the Supplementary Material). Specifically, after conducting multiple linear regression controlling for number of posts per user, partial regression coefficients indicated that users with lower posting latency had a higher habit weight (partial regression coefficient for posting latency: β = -0.014 [SE = 0.007], $p = 0.028$, partial regression coefficient for number of posts: β = 0.0000937 [SE = 0.0000468], $p = 0.045$) (Figure 4a) and a lower action learning rate in the confirmatory sample (partial regression coefficient for posting latency: β = 0.034 [SE = 0.006], $p < 0.001$, partial regression coefficient for number of posts: β = -0.000149 [SE = 0.0000412], $p < 0.001$) (Figure 4b). The relationship with reward learning rate remained not significant (partial regression coefficient for posting latency: β = -0.00515 [SE = 0.009], $p = 0.586$, partial regression coefficient for number of posts: β = -0.0000341 [SE = 0.0000591], $p < 0.564$) (Figure 4c).*
>
> *However, we note that posting latency did positively correlate with negative reward learning rate extracted from the RLH2 model (β = -0.014 [SE = 0.005], $p = 0.005$), suggesting that more frequent posters on Twitter are less likely to change their behaviour when they receive fewer Likes than they expected (Supplementary Material). This correlation remained significant after controlling for number of posts, using the preregistered one-tailed significance test (partial regression coefficient for posting latency: β = -0.014 [SE = 0.00613], p(one-tailed) = 0.045, partial regression coefficient for number of posts: β = -0.0000383 [SE = 0.0000437], p(one-tailed) = 0.191)."* (page 17, lines 447-460)

We then added the control to the results relating habit weight to age and gender (noting that significance does not change even for gender, as we preregistered a hypothesis that males would have a higher habit weight than females, and preregistered a one-tailed significance test for such hypotheses):

> *"Sensitivity analyses confirmed that these results remained significant after controlling for number of posts per user (see Preregistration Deviations in the Supplementary Material). Specifically, after conducting multiple linear regression, partial regression coefficients indicated that age significantly predicted habit weight (partial regression coefficient for age: β = 0.001 [SE = 0.0005], $p = 0.0217$, partial regression coefficient for number of posts: β = 0.0001 [SE = 0.00003], $p < 0.001$). Additionally, after conducting multiple linear regression using gender and number of posts to predict habit weight, and using our preregistered one-tailed significance test, partial regression coefficients indicated that males had a higher habit weight than females (partial regression coefficient for gender: β = 0.0298 [SE = 0.0156], p(one-tailed) = 0.0281, partial regression coefficient for number of posts: β = 0.0001 [SE = 0.00003], p(one-tailed) < 0.001)."* (page 19, lines 505-515)

We added the control to the results relating parameters to wellbeing:

“Sensitivity analyses controlling for number of posts confirmed that all relationships between parameters and wellbeing remained nonsignificant (see Preregistration Deviations in the Supplementary Material). Specifically, multiple linear regression controlling for number of posts confirmed there was no significant relationship between wellbeing and habit weight (partial linear coefficient for wellbeing: β *= -0.0246 [SE = 0.0800], p = 0.759, partial quadratic coefficient for wellbeing:* β *= -0.006 [SE = 0.0131], p = 0.606, partial linear coefficient for number of posts:* β *= 0.000157 [SE = 0.0000373], p < .001), nor wellbeing and action learning rate (partial linear coefficient for wellbeing:* β *= -0.0927 [SE = 0.0725], p = 0.201, partial quadratic coefficient for wellbeing:* β *= 0.0145 [SE = 0.0181], p = 0.222, partial linear coefficient for number of posts:* β *= 0.000297 [SE = 0.0000376], p < .001), nor wellbeing and reward learning rate (partial linear coefficient for wellbeing:* β *= 0.00769 [SE = 0.0123], p = 0.531, partial linear coefficient for number of posts:* β *= -0.0000364 [SE = 0.0000420], p = 0.387).” (*page 21, lines 560-572)

We then reported this control in the section ‘Statistical Inference’ in ‘Methods’:

“To control for effects of number of posts on parameter values, we repeated all individual difference analyses in the main paper (i.e., analyses relating posting latency, age, gender and wellbeing to parameter values) controlling for number of posts. This control deviated from our preregistration; for further explanation, see ‘Preregistration Deviations’ in the Supplementary Material.” (page 37, lines 1053-1058)

We also provide an explanation for the added control in a new row in the 'Preregistration Deviations' table in the Supplementary Material (page 39, line 791):

Original plan	***Revised plan***	***Justification for deviation***
We originally intended to compare individual differences (i.e., posting latency, age, gender, and wellbeing) to parameters without controlling for number of posts.	*We added sensitivity analyses controlling for number of posts per participant in all linear models relating individual differences to model parameters in the main paper.*	*We discovered in a simulated dataset that number of posts was related to both the fitted habit weight and action learning rate parameters of the RLH1 model, suggesting that relationships between frequency and other individual differences could be partly influenced by aspects of the model fitting procedure. We therefore now add new analyses to all individual-difference analyses in the main paper, controlling for number of posts to verify that our individual differences results were not driven by this artefact of the fitting procedure.*

Table R2 (Taken from Table S2). Table S2 contains Preregistration Deviations. The row relating to the control of number of posts is contained in Table R2.

R2.8
Parameter values. It would be great with a table with the average estimated parameter values for the different models and samples.

We have now added a section in the Supplementary Material with a table of average estimated parameter values for each model and sample, as shown below:

> *"Table S1 contains information about the fitted parameter values for all models in both the discovery and confirmatory samples.*

**Model**	**Parameter**	**Dataset**	**Minimum**	**Maximum**	**Mean**	**Median**	**Standard deviation**
Fixed Policy (FP)	Policy	Discovery	0.028	4.589	1.994	1.830	1.157
		Confirmatory	0.065	4.605	1.961	1.760	1.120
Changing Policy (CP)	a	Discovery	-67.059	2245.55	107.031	48.297	179.270
		Confirmatory	-53.008	4856.995	133.187	53.538	281.105
	b	Discovery	-42335.830	2245.540	52.182	48.301	1419.545
		Confirmatory	-12562.360	4855.874	121.6841	52.453	435.103
	c	Discovery	-0.119	5.285	0.067	1.28 x 10^{-5}	0.396
		Confirmatory	-0.106	4.505	0.041	1.20 x 10^{-5}	0.264
Pure Habit (PH)	Action learning rate	Discovery	4.99 x 10^{-9}	0.841	0.107	0.092	0.075
		Confirmatory	5.18 x 10^{-9}	0.711	0.114	0.098	0.083

Single learning rate RL (RL1)	Reward learning rate	Discovery	1.21×10^{-15}	0.941	0.040	0.013	0.071
		Confirmatory	8.82×10^{-16}	0.993	0.044	0.014	0.087
	Vigour cost constant	Discovery	2.76×10^{-6}	14656.469	7.495	1.482	60.552
		Confirmatory	1.70×10^{-5}	828.588	4.511	1.432	26.088
Double learning rate RL (RL2)	Positive reward learning rate	Discovery	1.66×10^{-14}	1	0.188	0.006	0.326
		Confirmatory	1.43×10^{-14}	1	0.185	0.006	0.329
	Negative reward learning rate	Discovery	2.32×10^{-11}	0.992	0.060	0.019	0.125
		Confirmatory	9.93×10^{-13}	1.00	0.061	0.019	0.119
	Vigour cost constant	Discovery	2.76×10^{-6}	4981.01	11.640	1.579	153.594
		Confirmatory	2.70×10^{-5}	672.491	5.327	1.590	22.476
Single learning rate RL-habit (RLH1)	Reward learning rate	Discovery	3.042×10^{-14}	1	0.223	0.035	0.325
		Confirmatory	2.244×10^{-14}	1	0.227	0.032	0.330
	Vigour cost constant	Discovery	1.848×10^{-10}	2243.037	9.549	1.575	85.707
		Confirmatory	8.957×10^{-10}	886.862	4.680	1.424	24.218
	Action learning rate	Discovery	2.684×10^{-10}	1	0.228	0.127	0.266
		Confirmatory	7.023×10^{-11}	1	0.242	0.136	0.272
	Habit weight	Discovery	1.281×10^{-09}	1	0.767	0.923	0.287
		Confirmatory	2.248×10^{-10}	1	0.767	0.944	0.295
Double learning rate RL-habit (RLH2)	Positive reward learning rate	Discovery	1.023×10^{-12}	1	0.262	0.015	0.361
		Confirmatory	7.510×10^{-14}	1	0.261	0.016	0.360
	Negative reward learning rate	Discovery	1.586×10^{-10}	1	0.233	0.109	0.281
		Confirmatory	9.450×10^{-11}	1	0.222	0.010	0.274
	Vigour cost constant	Discovery	5.057×10^{-5}	277.576	3.634	1.345	9.237
		Confirmatory	2.868×10^{-9}	105.552	3.731	1.407	7.123
	Action learning rate	Discovery	7.039×10^{-12}	1	0.234	0.125	0.276
		Confirmatory	6.986×10^{-11}	1	0.256	0.148	0.282
	Habit weight	Discovery	1.536×10^{-10}	1	0.762	0.960	0.304
		Confirmatory	1.633×10^{-8}	1	0.760	0.976	0.313

Table R3 (Table S1). List of parameter values for each model in the discovery and confirmatory datasets."

R2.9
Finally, I could not find any information about any start value for the policy or the reward estimate.

We have now added information about the start value for the policy for the pure RL models (RL1 and RL2), the Habitual Policy model (HP), and the hybrid RL-habit models (RLH1 and RLH2), as well as expected reward for RL1, RL2, RLH1 and RLH2, to the Methods section.

Additionally, to demonstrate that our main results are robust to different start values for policy and reward estimate, we re-ran model comparison with eight alternative ways of estimating start values, and demonstrated that the RL-habit hybrid models win every time, indicating that our results are not dependent on the specific way in which we estimated the start values for the policy and reward estimates. We added these to a new section in the Supplementary Material (‘Model comparison with different initializations’).

The information in the main paper, and references to the Supplementary Material, are below:

Habitual Policy (HP, formerly PH):

> *“The initial H is estimated from the data as the first posting latency in each user’s dataset, but we confirmed that our model comparison results were robust to this approximation by repeating analyses with different initializations (Supplementary Material).”* (page 31, lines 894-896)

Single Learning Rate RL (RL1):

> *“The initial values for both $Policy$ and expected reward $\hat{R}$ were estimated from the data, and we confirmed that our main model comparison results were robust to different initialization decisions by repeating model comparison with both $Policy$ and $\hat{R}$ initialized using a variety of different values (Supplementary Material). In the main analyses, $Policy$ is initialized as C divided by the mean value of Likes across all posts for that user, while $\hat{R}$ is initialized as the number of rewards on the first post.”* (page 33, lines 944-949)

Double Learning Rate RL (RL2):

> *“The double learning rate RL model differs from RL1 only in that rather than a single reward learning rate α_R, there are two separate reward learning rates for positive and negative prediction errors; ${\alpha_R}^P$ and ${\alpha_R}^N$ respectively.”* (page 34, lines 959-961)

Single Learning Rate RL-habit Hybrid (RLH1):

> *“The initial values for $Policy$, habit strength H and expected reward $\hat{R}$ were estimated from the data as follows. As with the pure RL models, the initial $Policy$ was set as the vigour cost constant C divided by the mean expected reward and the initial expected reward, $\hat{R}$, was set as the number of Likes on the first post, while the initial H was set as equal to the initial $Policy$. Robustness analyses confirmed that model comparison results were robust to varying these initializations (Supplementary Material).”* (page 34, lines 981-987)

The sensitivity analyses in the Supplementary Material are below:

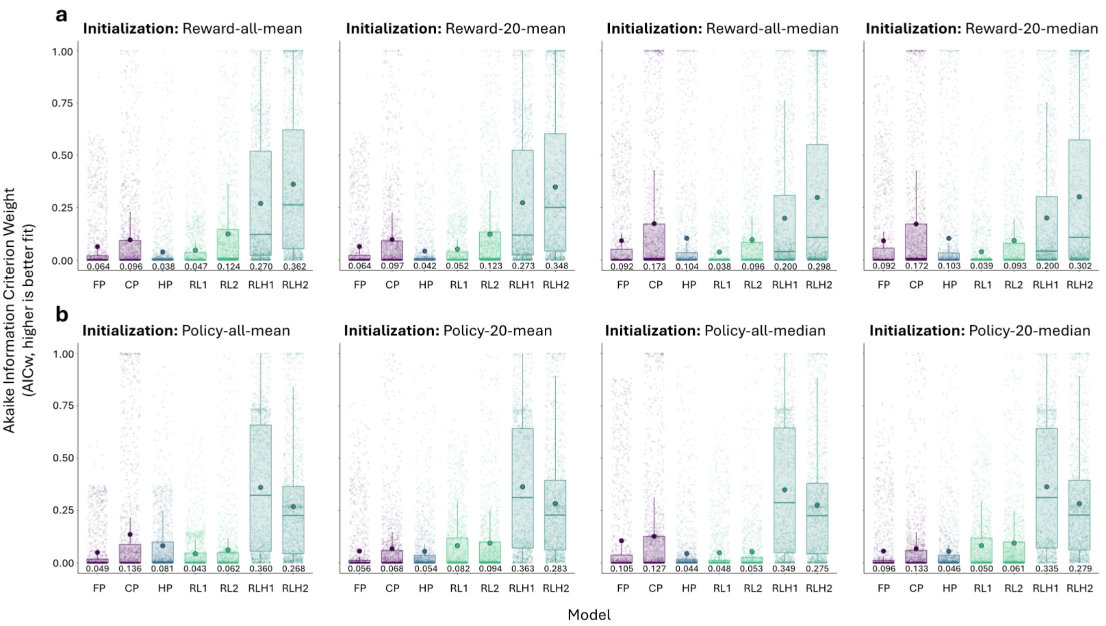

"***Figure R8 (Figure S9). Model comparison with different initializations in the confirmatory sample.*** *Model comparison did not differ substantially for eight different ways of estimating the initial policy and expected reward from the data. Higher AICw represents a better fit. Outlined circles represent the mean AICw, which is also indicated as numbers along the top of the x-axis (the criterion for which best fitting model was selected), while box plots represent the median and interquartile range for AICw, and scattered points represent the AICw for each individual user.* ***a)*** *Model comparison results for models fit using the rewards for each user to estimate starting values.* ***b)*** *Model comparison results for models fit using the posting latencies for each user to estimate starting values.*

The RL, habit and hybrid RL-habit models all estimated start values for policy and expected reward from the data. The main paper describes how these were estimated for all main analyses (Methods). Here, to test whether our results were robust to the way in which these values were initialized, we re-ran model comparison with eight different ways of estimating both policy and expected reward from the data, which vary across three dimensions: (i) whether the rewards (Likes) or the posting latencies were used in the estimate, (ii) whether all posts in a user's data or the just first 20 posts were used, and (iii) whether the mean or median of the relevant data points was used. Our results confirmed that our model comparison was robust to all these different ways of estimating initial expected reward and policy (Figure S9).

The eight different initialization schemes are as follows. In 'Reward-all-mean', the initial value for estimated reward in RL1, RL2, RLH1 and RLH2 was the mean of all rewards in that user's data. The initial policy in RL1, RL2, RLH1 and RLH2, as well as the initial habitual tendency in RLH1 and RLH2, was the vigour cost constant divided by this mean reward. The initial policy in the HP model was the same as in the main paper, the first posting latency for that user. In 'Reward-20-mean' all initializations were the same except the mean was taken of the first 20 rewards (Likes) for each user rather than all rewards. 'Reward-all-median' and 'Reward-20-median' were the same as 'Reward-all-mean' and 'Reward-20-mean', respectively, except that in each case medians were taken rather than means.

In 'Policy-all-mean', the initial value for estimated reward in RL1, RL2, RLH1 and RLH2 was the vigour cost constant divided by the mean of all posting latencies in that user's data. The initial policy in RL1, RL2, RLH1, RLH2 and HP models, as well as the initial habitual tendency in the RLH1 and RLH2 models, was also this mean of all posting latencies in that user's data. Policy-20-mean, Policy-all-median and Policy-20-median were calculated by using the mean latency of the first 20 posts instead of all posts, the median of all posts, and the median of the first 20 posts, respectively." (page 27, lines 563-605)

We thank R2 for their review, which prompted a range of sensitivity analyses and explanations which we believe have strengthened the validation of our results.

Reviewer #3 (Remarks to the Author):

This paper puts forward a hybrid model of reinforcement learning and habitual processes involved in social media posting behavior. I applaud the authors for their creative thinking and their research practices, including an

extensive (and adhered to!!!) preregistration, extensive model simulations, model comparison and model falsification. Their modeling code seems well-organized and well-commented, aiding use of their model (although I have not tried). Although I think the authors delivered a great paper already, I do have some considerations for them to make the paper clearer and even more convincing.

We are grateful to R3 for their positive comments.

Major points:

Introduction

R3.1
1. The introduction is clear and well-written. As somebody outside of the social media field, some sentences require additional explanation. For example, I can picture what you mean with "diversity" in lines 77/78 "Critically, the dose-response approach can neither account for the diversity of social media users and behavior", but in the part after that – "nor generate findings which are robust to the constantly changing social media landscape." – I struggle to visualize "the constantly changing social media landscape".

We thank R3 for their positive appraisal of our introduction. We have edited this sentence for clarification:

> *"Further, social media fluctuate in platform and algorithm design on a much faster timescale than typical scientific research(Kaye et al., 2020; Matias, 2023; Orben, 2020; Orben & Matias, 2025). Therefore, dose-response approaches which only test 'doses' of specific social media iterations will not generate findings that are robust to these changes."* (page 3, lines 78-79)

The same holds for "However, there are many ways in which this statistical signature might be operationalized" (lines 133/134) and for the "behavior-environment mismatch" on lines 89/90. Examples would help.

We thank R3 for the opportunity to clarify these points, we have edited both parts:

> *"However, there are many ways in which this statistical signature might be operationalized, making it difficult to establish which signatures truly reflect the hypothesized generative process. For example, 'rewards received' could be quantified using absolute rewards on a previous post(Anderson & Wood, 2023), or the average number of rewards received across posts on a previous day(Mirea et al., 2024)."* (page 5, lines 142-144)
>
> *"Such features could therefore produce a behaviour-environment 'mismatch', wherein human behavioural tendencies which were beneficial in the environments we evolved in become vulnerabilities in evolutionarily novel environments. For example, the human preference for low-effort rewards might have originally evolved to help conserve energy, but could become a vulnerability when online rewards such as Likes are significantly lower effort to give and receive than many offline rewards(Turner et al., 2024). This behaviour-environment mismatch could therefore drive excessive social media use, with downstream impacts on individuals and society."* (page 3, lines 91-98)

Method

R3.2
2. How were values (both in the RL part and in the Habit part of the model) initialized? It doesn't seem so straight forward to decide which initial expectation individuals have about receiving likes while this initialization can have a large effect on (asymmetric) learning rates.

We thank R3 for this important point, which was also raised by R2 (R2.9). In the response to R2.9 above, we detail how we have now added information in the main paper about how the values were initialized, and added a section in the Supplementary Material ('Model comparison with different initializations') in which we re-run model comparison with eight different initializations of expected reward and policy, showing that the key results are robust to these changes.

Regarding R3's point that initializations can have a large effect on learning rates, we note that no inference was preregistered or made about learning rates, apart from null results relating reward learning rate to habit weight and wellbeing. However, to investigate the effects of different initializations on learning rates, we correlated fitted RLH1 learning rates for each initialization with each other. All correlations between learning rates fitted with different initializations were significant at $p < 0.001$.

Results

R3.3
3. I really appreciate you preregistering the project, sticking to the preregistration, and clearly reporting deviations in the supplements. Yet, I wonder whether relating posting frequency to habitual model parameters is more of a model check then a primary goal, and should therefore have a less prominent place in the paper.

We are thankful for R3's appreciation of the preregistration. The primary goal of the paper is to propose and validate a computational model of posting behaviour which explains both reward learning and habits. We have endeavoured to make this apparent from the title, abstract and introduction of the paper. However, we also believe that the relationship between posting frequency and habit parameters is an empirical finding which helps us to understand how habits manifest in social media posting and may attract broad interest from computational scientists to practitioners. We therefore chose to retain these results in Figure 4.

R3.4
4. I believe that the evidence for a positivity bias is very limited and that claims should therefore be removed throughout. The RLH1 outperforms the RLH2 in the confirmatory sample and, judging from the plot in Figure 2 of the preregistration, the difference in learning rates in the discovery sample is negligible (as also reflected in the 0.049 p-value). Although you do state that "the findings relating to positivity bias should be interpreted with caution given that the winning model across the sample has a single learning rate (RLH1)", this is only done so in the supplements and claims about the positivity bias within the text are unnuanced.

We thank R3 for this point, and therefore as suggested we have removed references to finding a positivity bias within the Abstract and text throughout. However, we retain references to the fact that we initially tested a model with a positivity bias, to emphasise that our modelling approach allowed us to assess for the presence of a positivity bias, although we found no strong evidence for one.

In the Abstract, we removed the reference to the model as including a 'learning bias'.

In the Introduction, we emphasise that our model allows us to *test for* (not find) a positivity bias:

> *"Importantly, this novel computational model of real-world data allows us to quantify the relative contribution of the goal-directed vs. habitual system for each individual, as well as to test for the presence of a 'positivity bias', the well-documented propensity to learn at a higher rate from information which is more rewarding relative to expectations."* (page 6, lines 175-178)

In the Results, we added a sentence emphasising the lack of positivity bias in the confirmatory sample:

> *"The inconsistency across samples in whether the winning model had a valence bias likely reflects individual variation across users in whether they were better fit by RLH2 or RLH1. However, overall, given that the RLH1 model wins in the confirmatory sample, we can conclude that there is little evidence for a valence bias (or specifically for a positivity bias)."* (page 13, lines 331-335)

In the Discussion, we have added a qualifying sentence when we refer to a potential positivity bias:

> *"This is consistent with a 'positivity bias' wherein humans learn more from positive than negative rewards, even as the habitual system takes control of behaviour (Palminteri & Lebreton, 2022). However, we note that the best-fitting model across users (RLH1) did not have a positivity bias, therefore evidence for a positivity bias in this dataset is weak and future work will be needed to establish whether, and if so under what conditions, humans exhibit a positivity bias when learning from social media rewards."* (page 23, lines 624-627)

R3.5
5. This may be a plotting function issue, but the regression lines (especially in Figure 5C/5D) suggest non-linearity to me. Did you test for linear as well as non-linear relation6ships? If so, this is missing in the text. If not, why did you only do this for wellbeing?

We indeed tested for nonlinear relationships only for wellbeing for two reasons. First, we had strong prior hypotheses that wellbeing would be related to reward learning parameters building on previous work, so given that wellbeing was not related linearly in the discovery dataset, we also chose to explore nonlinear relationships. Secondly, in the discovery sample we found significant nonlinear relationships between wellbeing and both habit weight and action learning rate, therefore we preregistered that we would investigate if these replicated in the confirmatory dataset (and found they did not).

We now add this explanation to the Results section of the main paper:

> *"Because we had strong prior hypotheses that wellbeing would relate to reward learning based on previous literature(Berwian et al., 2020; Huys et al., 2016; Mirea et al., 2024), we explored for nonlinear relationships in the discovery sample. We found nonlinear (i.e., both linear and quadratic) relationships*

between wellbeing and both habit weight and action learning rate in the discovery sample. Therefore, to see if these quadratic relationships replicated, we here tested for linear and quadratic relationships between wellbeing and both habit weight and action learning rate, and just for a linear relationship between wellbeing and reward learning rate." (page 20, lines 531-538)

We also now add detail about the quadratic relationships to the caption of Figure 5:

> ***"c)** Wellbeing had no significant linear or quadratic relationship with habit weight. **d)** Wellbeing had no significant linear or quadratic relationship with action learning rate. **f)** Wellbeing had no significant linear relationship with reward learning rate."* (page 18, lines 490-493)

Discussion

R3.6
6. With the current societal discussion on individual differences in effects of social media use, I see it as a missed opportunity to not quantify individual differences in model parameters or even in the cognitive processes involved in social media use. You already have these data because you fit models individually and, judging from the model parameter plots in the supplements and from the model fit plot in Figure 3A, individual differences in both are substantial. I understand you cannot do everything in one paper, but as you do relate individual differences to demographics and well-being, mentioning individual differences in the discussion would already bring added value.

In response also to R2.8 above, we have added a new table in the Supplementary Material (Table S1, Table R3 above), which includes properties of the distribution of each parameter for each model in both the discovery and confirmatory samples, including minimum, maximum, mean, median and standard deviation. This provides more quantitative detail about the individual variation in fitted parameters across our samples.

Due to the novelty of the modelling results and individual differences without interactions, there is no capacity to add further analyses in this study. However, we have added a sentence recommending such work in future in the Discussion:

> *"Indeed, the highly-spread parameter distributions (Table S1, Supplementary Material) indicate substantial individual variation in reward learning processes which is yet to be explored and explained in future work."* (page 26, lines 720-722)

R3.7
7. I find the discussion somewhat uncritical. The title suggests that the main goal of the project is to put forward "a computational model of reward learning and habits on social media". Because of this, I expected you to discuss the shortcomings of the model. Although you do mention these, you only do so in the supplements (line 605-607: "We document the specific simplifying assumptions of our model in the Supplementary Material, so that future work can reintroduce parameters into the model to account for more complexity.").

We thank R3 for this point, which was also made by R2 (R2.1). In response to this and R2's, point, we have now edited this section of the Discussion to make the limitations of the model clearer:

> *"It is important to note, however, that as an initial step modelling multiple cognitive processes in real-world social media behaviour, both our model and dataset are simplifications of a complex process. We document the specific simplifying assumptions of our model in the Supplementary Material. For example, our model considers only two generative processes underlying posting – the habitual system and model-free RL, whereas in reality there are likely to be many more interactive cognitive processes governing posting – for example, model-based RL(Turner et al., 2024) and social learning(Brady et al., 2021). Further, our model assumes that rewards are delivered on a ratio schedule, such that the amount of Likes received does not depend on posting latency. This is unlikely to be the case in the real world, where it is possible that Likes do depend to some extent on latency (for example, because more frequent posting increases visibility via the algorithm, or because too frequent posting decreases the likelihood that followers will Like every post), and that social media users actively learn a relationship between posting latency and Likes received. Additionally, our model assumes that all 'actions' (i.e. posts) are the same, whereas in reality, social media users may consider posts with different content as separate 'actions' which each have different reward schedules."* (page 25, lines 692-707)

R3.8
8. Relatedly, you state that "model recovery was excellent" with 1000 posts. I don't think I agree. If I look at the column of the best-fitting model (RLH1) in Figure S1b, the AIC weight of the corresponding model (RLH1) is about .5. The AIC weight of the PH about .3. Dividing these values gives you that the RLH1 is only 1.66 times

more likely to be the underlying model than the PH. I wouldn't call that excellent, especially if the average number of posts is lower than 1000. I don't want to be nitpicky and I think you have already done enough simulation work, but if I'm a social media researcher without much modeling experience that wishes to apply your hybrid model, I would want to be sure that it is recoverable. If it is barely recoverable at 1000 posts, I would be worried. I suggest providing some discussion on this point. What is your feeling about the required number of posts based on your simulations?

We thank R3 for the opportunity to more explicitly discuss and specifically explain our model recovery procedure, which we agree will greatly facilitate easier reuse of our model by social media researchers.

We have first removed the statement that 'model recovery was excellent', which may appear a somewhat subjective assessment. It is unfortunate that there is indeed no current consensus on how to qualify different degrees of model recovery, similar to different degrees of other statistical measures such as test-retest reliability (Vrizzi et al., 2025).

Therefore, we opted to better discuss and comment on the objective performance of our model recovery. Specifically, we added a discussion to the section about model recovery in the Supplementary Material which considers R3's point about high evidence from model comparison for HP when RLH1 is the winning model:

> *"We first performed recovery for 1000 posts (Figure S2b). Encouragingly, this model recovery analysis reveals higher mean AICw values in the diagonals than at any other location in the matrix. This means that for each of the seven models, if that model represents the true generative process in a dataset, then that model will also be the 'best fitting' model according to our model fitting procedure (higher mean AICw for that fitting model than for any other model in each row).*
>
> *However, we also note that model recovery is not perfect. For example, the RLH1 column (Figure S2b) indicates that with 1000 posts, when RLH1 is the winning fitted model, model comparison yields a fairly high possibility that the HP model generated the underlying dataset. However, in this case, model simulation and falsification can be used to test whether the statistical signature of RL is present in a given dataset. If it is, the HP model, which includes no RL component, can be absolutely rejected, regardless of the relative evidence for HP in model comparison. This thus underscores the importance of combining model comparison, a measure of the evidence for each model relative to the set of models considered, with model falsification, an absolute criterion for model rejection(Palminteri et al., 2017). Further, we also note that in Figure S2b, the CP model is poorly recovered and is often incorrectly recovered as the FP. This is a result of the parameters that the CP model was simulated with, which resulted in a mostly consistent policy across time. As the FP and CP models were baseline models, good model recovery between these two is not important for our research questions."* (page 14, lines 293-306)

Regarding the required number of posts for future research, we note that the results of both model and parameter recovery can depend greatly on specific features of a given dataset such as parameter and reward distributions. For example, it will be much harder to recover an RL process where learning rates are typically near 0, compared to an RL process with very high learning rates, or to distinguish between models with one and two learning rates when the learning rates in the double learning rate model are very close to each other. We therefore now add a discussion about how future researchers should select their number of posts based on specific features of their dataset, with separate recommendations for researchers with more vs. less experience with modelling:

> *"We therefore suggest, from this initial validation, that 80 posts function as an initial rubric for minimum number of posts in any similar future research. However, we ideally recommend that future researchers employing our modelling procedure use a process specific to their dataset and research question to select sample size. First, model recovery with a selected number of posts should be run with parameter and reward distributions reflective of those in the specific dataset under consideration, as these variables in a dataset can have large effects on model and parameter recovery(Wilson & Collins, 2019). For example, the ability to recover models with a double learning rate depends on the distribution of the learning rate parameters: it will be easier to distinguish models with double from single learning rates in model recovery if the learning rates in the double learning rate model have a larger difference from each other. Secondly, other considerations such as minimising number of participants excluded may contribute to lowering or raising the exclusion criterion for number of posts. Third, the specific research question of interest should be considered. We were most interested in the underlying generative process for our data, so model recovery analysis was important in considering sample size. However, for example, if a researcher is more interested in how a specific parameter value changes across populations or conditions, more weight should be given to the results of a parameter recovery analysis for this parameter than model recovery in determining minimum number of posts."* (page 15, lines 320-338)

We would also like to address R3's specific points about identifying whether the RLH1 model underlies the data. We here reproduce the model recovery matrix, to which we have also added exact numbers to complement the shading, to facilitate these kinds of calculation:

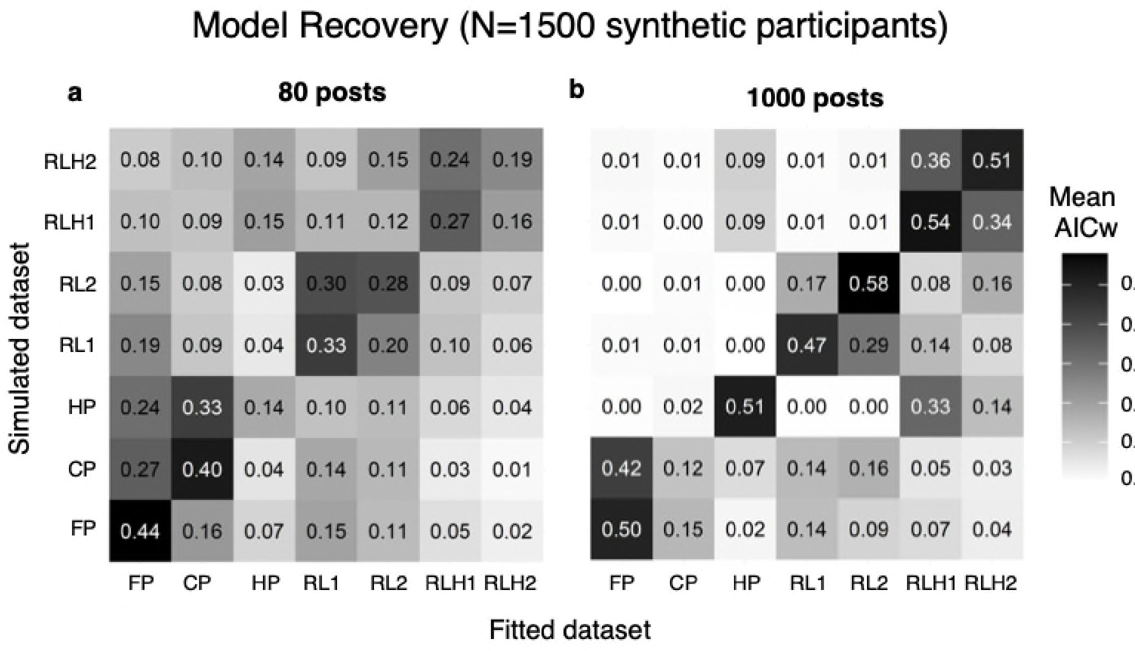

*"**Figure R9 (Figure S2). Model recovery.** FP = Fixed Policy, CP = Changing Policy, HP = Habitual Policy, RL1 = Pure RL, single learning rate, RL2 = Pure RL, double learning rate, RLH1 = Hybrid RL-habit, single learning rate, RLH2 = Hybrid RL-habit, double learning rate. Shaded colours represent mean AICw for each fitted model across each simulated dataset. Better model recovery is indicated by high mean AICw for the same model fitted to the simulated dataset (i.e., across the diagonal). **a)** Model recovery for 80 posts shows that while the correct model is not always recovered, there is a bias towards the simpler models (those with fewer parameters). Therefore, there is no bias towards the RL-habit models which won in the empirical dataset, reducing the likelihood of false positives and making our model fitting procedure a conservative test of the RL-habit models. **b)** Model recovery for 1000 posts shows excellent recovery. High mean AICws outside of the diagonal are primarily for models which share components – for example, the HP model is sometimes recovered as the RLH1 model, which makes sense as the HP model is a component contained within the RLH1 model; indeed the RLH1 model reduces to HP when habit weight is 1. There is also some confusion between the RL1 and RL2 models, and RLH1 and RLH2 models, which can be attributed to their very similar structure."*

Because AICw represents the probability that each model is the best model given the data *and the set of candidate models,* in order to quantify the evidence for a specific underlying process, it is necessary to compare the combined AICw of all models with vs. all models without that process (Wagenmakers & Farrell, 2004). When assessing the evidence for RLH1, for example, you might be interested in whether (i) a pure habit or a hybrid RL-habit model underlies the data, and (ii) whether the RL process underlying the data has one or two learning rates.

To answer (i), to compare AICw between models with and without a specific component, you can add the AICw for all models with the component and compare it to all models without the component (Wagenmakers & Farrell, 2004). So, to compare AICw between habit models with and without an RL component, regardless of learning rate asymmetry, rather than simply using AICw(RLH1), you might combine the AICw of both RLH1 and RLH2, and calculate:

$$\frac{AICw(RLH1) + AICw(RLH2)}{AICw(HP)}$$

For model recovery with 1000 posts, model comparison thus suggests that given that RLH1 is the best fitting model, then given the set of candidate models, it is (0.36 + 0.54)/0.33 = 2.73 times more likely that an RL-habit hybrid model rather than the HP model underlies the data (rather than 1.64 times from considering RLH1 alone). (Depending on the components you are interested in, you could also combine these in different ways; e.g. if you are just interested in whether an RL process is present at all, you might calculate the combined AICw of all RL and RL-habit models, compared with the combined AICw of FP, CP and HP models, etc.) Furthermore, you can then combine this conclusion with the model falsification procedure in your dataset to investigate whether the

dataset exhibits a statistical signature of RL, and if so firmly reject the hypothesis that the HP model underlies the data, regardless of its performance in model comparison.

To answer (ii), you might then compare the AICw between RL models without vs. with two learning rates:

$$\frac{AICw(RL1) + AICw(RLH1)}{AICw(RL2) + AICw(RLH2)}$$

In this way, a combination of both model comparison and model falsification would allow a researcher to firmly reject the hypothesis that the HP model underlies the data, even if focusing on model recovery alone, and comparing just RLH1 and the HP model, would not appear initially to provide as strong evidence.

Minor points:

R3.9
9. Even after reading the supplemental materials, I'm having trouble visualizing what vigour costs mean. I suppose you need the vigour cost parameter to allow for differences in the relationship between expected rewards and policies across individuals, but what this parameter captures in real life is vague to me. Please elaborate.

We thank R3 for the opportunity to further clarify both the computational and real-world interpretation of vigour cost. We have now edited the paper to provide a more in-depth explanation of this parameter. First, we have renamed the parameter from 'cost' to 'vigour cost constant', which is what it was called in the original paper by Niv (Niv et al., 2007). This better captures its mathematical function, which is the constant describing the rate at which the vigour cost of posting decreases as posting latency increases. The vigour cost constant parameter is necessary not just to allow for differences in the relationship between expected rewards and policies across individuals, but also, for each individual, to prevent the optimal policy from simply being to post infinitely fast.

We have first added a section in the original description of the models in the main text in which we describe in more detail both the computational function and real-world interpretation of this parameter:

> *"The vigour cost constant C associates each posting action with a 'vigour cost', where the vigour cost of action τ_{Post^t} is equal to $\frac{C}{\tau_{Post^t}}$, and thus is higher for shorter posting latencies[42,48]. Mathematically, the vigour cost constant represents the rate at which the vigour cost decreases as posting latency increases. If there were no (subjective) costs associated with posting, there would be no reason not to try to post infinitely fast, regardless of reward. However, given that each action (i.e., each post) is associated with a subjective cost, it is only worth increasing posting frequency if the corresponding expected reward from doing so justifies the increased cost.*
>
> *In the real world, the 'cost' of posting might correspond to any factor which deters an individual from posting fast – such as the increased cognitive effort as well as the opportunity cost of time associated with posting more quickly instead of engaging in other activities. The 'vigour cost' of posting specifically denotes any effort and time which is greater for each post at a higher frequency than for each post at a at lower frequency – as the vigour cost per post, $\frac{C}{\tau_{Post^t}}$, is greater for smaller τ_{Post^t}. For example, posting very fast on Twitter might be more costly relative to posting slowly because posting fast requires one to actively search for new material to post about rather than simply posting whenever news comes up, as well as to post more creatively to ensure less overlap with multiple other recent posts. A figure showing how varying the vigour cost constant C affects the relationship between expected reward and policy, is contained in the Supplementary Material."* (page 32, lines 923-929)

We have also added more detail and explanation to the description in the Supplementary Material where we demonstrate the mathematical function of the vigour cost constant, including a new figure:

> "Specifically, C represents the vigour cost constant, which determines the vigour cost of each action, $\frac{C}{\tau_{Post^t}}$, with the vigour cost increasing for actions with shorter latencies. In the real world, the vigour cost might represent any subjective cost which is greater for higher frequency posting, such as the increased effort required to actively come up with more varied and creative posts rather than simply wait until a good idea for a post comes along. As shown in Figure S1, for a given expected external reward per post (i.e., expected amount of Likes), a higher vigour cost constant will result in a higher policy (i.e., a slower posting latency). This is because a higher vigour cost constant translates to a higher subjective cost of posting, which decreases the subjective reward, and results in a policy to post at longer latencies (less often).

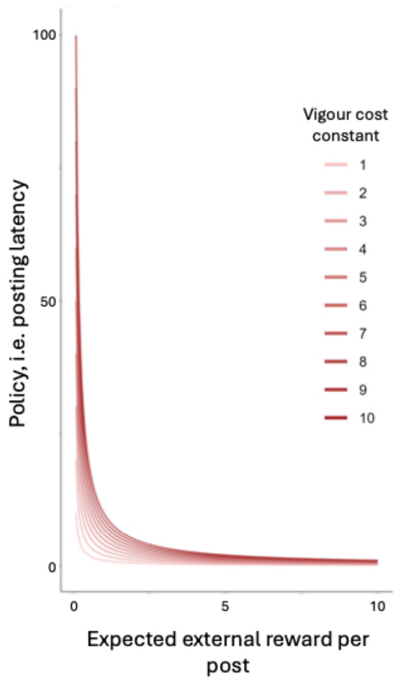

Figure R10 (Figure S1). The relationship between expected external reward (Likes) and policy in the RL model depends on the vigour cost constant. *For a higher vigour cost constant, the policy will be higher for a given expected reward."* (page 6, lines 143-160)

R3.10
10. I may have missed this but what was the average number of posts? It would help to add this to Table 1 in order to put the simulation results (in which you used 80 and 1000 posts) into perspective.

We thank R3 for this suggestion. It was also suggested by R1, and we have therefore added the minimum, maximum, mean, median and standard deviation of number of posts per person to Table 1 (also Table R1, above in response to R1.4).

R3.11
11. I would consider changing some of the model names. I (and probably other RL modelers) associate PH with the Pearce-Hall model. Besides, the P sometimes means "pure" (as in PH) and sometimes "policy" (as in CP).

We thank R3 for this point, and have changed 'Pure Habit (PH)' to 'Habitual Policy (HP)' throughout.

R3.12
12. You made lovely plots. For that reason (and for conciseness), I would remove Table 2 (which information is already in Figure 3A) and put test statistics in the plots instead of in the text, which often doubles the information in the plots (e.g., Figure 3B and the paragraph starting at line 370 "As expected…").

This is a good point which was also suggested by R1 (R1.7, above). We have now added test statistics in all figures throughout, and removed Table 2 (see Figure R3 above for an example). We have now added the statistics for Table 2 into Figure 3, see below:

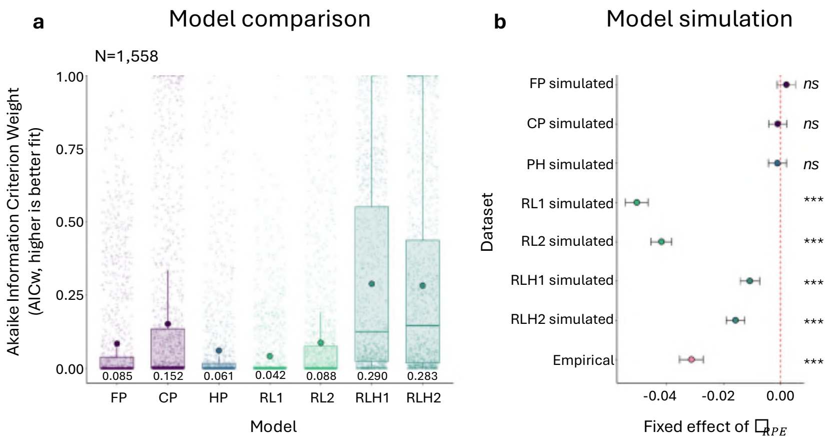

*“**Figure R11 (Figure 3). Model selection characterises the underlying learning process across users. a)** Model comparison in the confirmatory sample. FP = Fixed Policy, CP = Changing Policy, HP = Habitual Policy, RL1 = Pure RL, single learning rate, RL2 = Pure RL, double learning rate, RLH1 = Hybrid RL-habit, single learning rate, RLH2 = Hybrid RL-habit, double learning rate. Higher Akaike Information Criterion weight (AICw) represents a better fit. Outlined circles represent the mean AICw, which is also indicated as numbers along the top of the x-axis (the criterion for which best fitting model was selected), while box plots represent the median and interquartile range for AICw, and scattered points represent the AICw for each individual user. Model comparison reveals that RLH1 provided the best description of behaviour. **b)** Model simulation reveals that an RL component is present in the empirical confirmatory sample. Dots show estimates of β_{RPE}, error bars show 95% confidence intervals, the column on the right indicates significance, according to the following scheme: ns = not significant, * = p<0.05, ** = p<0.01, *** = p<0.001. The simulated datasets without an RL component do not show a significantly negative fixed effect of , while the simulated datasets with an RL component do. As the empirical dataset also shows a significantly negative fixed effect of β_{RPE}, this falsifies the hypothesis that the FP, CP or HP models underlie the data.”*

R3.13
13. The reason for investigating how individual differences in model parameters relate to age, gender, and wellbeing is unclear to me. Why these variables and why no interactions for example?

Age, gender and wellbeing were all variables that are known to relate to variation in both reward learning strategies and social media use. We therefore chose to investigate these variables initially. We did not find interactions in our discovery sample, therefore we did not preregister to test for interactions. However, we hope that our proof-of-concept for the RL modelling approach will prompt future researchers to investigate further variables as well as interactions.

We have added a more explicit justification of this decision as well as a suggestion that future research investigates interactions, to the Discussion:

> *“While we initially focused on age, gender and wellbeing due to previous literature demonstrating that these variables relate to differences in both reward learning*(Altikulaç et al., 2019; Huys et al., 2016; Nussenbaum & Hartley, 2019) *and social media use(Fassi et al., 2023; Orben et al., 2022), we hope that this proof-of-concept for identifying individual differences in social media use will prompt future researchers to investigate other individual differences as well as interactions.”* (page 26, lines 722-726)

R3.14
14. Some typos I noticed: line 244 (Hih), line 440 (interquartlie), line 453 (“paired-samples” should be “independent-samples”?), line 708 (from from), Figure S4 (y-axis should be “Random” instead of “Fixed” effect?) and below that on line 301 (sample sample).

We thank R3 for their close attention to our work, and have now corrected all these typos.

To promote constructive reviewing and transparency, I always sign my reviews,
Jessica Schaaf

Reviewer #3 (Remarks on code availability):

A quick glance suggests the code is well-organized and well-commented. README file is present. I did carefully

compare the preregistration to the manuscript. A breath of fresh air. Precise and extensive. Almost no deviations, and if there were, they were outlined including reasoning in the supplements.

We thank R3 for their thorough review of our work, and suggestions which we believe have greatly improved the paper.

Reference list

Altikulaç, S., Bos, M. G. N., Foulkes, L., Crone, E. A., & van Hoorn, J. (2019). Age and Gender Effects in Sensitivity to Social Rewards in Adolescents and Young Adults. *Frontiers in Behavioral Neuroscience*, *13*. https://www.frontiersin.org/articles/10.3389/fnbeh.2019.00171

Anderson, I. A., & Wood, W. (2023). Social motivations' limited influence on habitual behavior: Tests from social media engagement. *Motivation Science*, *9*, 107–119. https://doi.org/10.1037/mot0000292

Berwian, I. M., Wenzel, J. G., Collins, A. G. E., Seifritz, E., Stephan, K. E., Walter, H., & Huys, Q. J. M. (2020). Computational Mechanisms of Effort and Reward Decisions in Patients with Depression and Their Association with Relapse after Antidepressant Discontinuation. *JAMA Psychiatry*, *77*(5), 513–522. Scopus. https://doi.org/10.1001/jamapsychiatry.2019.4971

Bogacz, R. (2020). Dopamine role in learning and action inference. *eLife*, *9*, e53262. https://doi.org/10.7554/eLife.53262

Brady, W. J., McLoughlin, K., Doan, T. N., & Crockett, M. J. (2021). How social learning amplifies moral outrage expression in online social networks. *Science Advances*, *7*(33), eabe5641. https://doi.org/10.1126/sciadv.abe5641

Camerer, C., Xin, Y., & Zhao, C. (2024). A neural autopilot theory of habit: Evidence from consumer purchases and social media use. *Journal of the Experimental Analysis of Behavior*, *121*(1), 108–122. https://doi.org/10.1002/jeab.897

da Silva Pinho, A., Céspedes Izquierdo, V., Lindström, B., & van den Bos, W. (2024). Youths' sensitivity to social media feedback: A computational account. *Science Advances*, *10*(43), eadp8775. https://doi.org/10.1126/sciadv.adp8775

Daw, N. D., Gershman, S. J., Seymour, B., Dayan, P., & Dolan, R. J. (2011). Model-based influences on humans' choices and striatal prediction errors. *Neuron*, *69*(6), 1204–1215. https://doi.org/10.1016/j.neuron.2011.02.027

Daw, N. D., & O'Doherty, J. P. (2014). Chapter 21—Multiple Systems for Value Learning. In P. W. Glimcher & E. Fehr (Eds.), *Neuroeconomics (Second Edition)* (pp. 393–410). Academic Press. https://doi.org/10.1016/B978-0-12-416008-8.00021-8

Dickinson, A. (1985). Actions and Habits: The Development of Behavioural Autonomy. *Philosophical Transactions of the Royal Society of London. Series B, Biological Sciences*, *308*(1135), 67–78.

Dolan, R. J., & Dayan, P. (2013). Goals and Habits in the Brain. *Neuron*, *80*(2), 312–325. https://doi.org/10.1016/j.neuron.2013.09.007

Doll, B. B., Simon, D. A., & Daw, N. D. (2012). The ubiquity of model-based reinforcement learning. *Current Opinion in Neurobiology*, *22*(6), 1075–1081. https://doi.org/10.1016/j.conb.2012.08.003

Fassi, L., Thomas, K., Parry, D. A., Leyland-Craggs, A., Ford, T. J., & Orben, A. (2023). *Social media use and internalising symptoms in clinical and community adolescent samples: A systematic review and meta-analysis* (p. 2023.09.12.23295355). medRxiv. https://doi.org/10.1101/2023.09.12.23295355

georgiaturner/rl_twitter: Code for 'A computational model of reward learning and habits on social media'. (n.d.). Retrieved 7 November 2024, from https://github.com/georgiaturner/rl_twitter

Greenstreet, F., Vergara, H. M., Johansson, Y., Pati, S., Schwarz, L., Lenzi, S. C., Geerts, J. P., Wisdom, M., Gubanova, A., Rollik, L. B., Kaur, J., Moskovitz, T., Cohen, J., Thompson, E., Margrie, T. W., Clopath, C., & Stephenson-Jones, M. (2025). Dopaminergic action prediction errors serve as a value-free teaching signal. *Nature*, 1–10. https://doi.org/10.1038/s41586-025-09008-9

Gremel, C. M., & Costa, R. M. (2013). Orbitofrontal and striatal circuits dynamically encode the shift between goal-directed and habitual actions. *Nature Communications*, *4*(1), 2264. https://doi.org/10.1038/ncomms3264

Hull, C. L. (1943). *Principles of behavior: An introduction to behavior theory* (pp. x, 422). Appleton-Century.

Huys, Q. J. M., Maia, T. V., & Frank, M. J. (2016). Computational psychiatry as a bridge from neuroscience to clinical applications. *Nature Neuroscience*, *19*(3), 404–413. https://doi.org/10.1038/nn.4238

Kaye, L., Orben, A., A. Ellis, D., C. Hunter, S., & Houghton, S. (2020). The Conceptual and Methodological Mayhem of "Screen Time". *International Journal of Environmental Research and Public Health*, *17*(10), Article 10. https://doi.org/10.3390/ijerph17103661

Lindström, B., Bellander, M., Schultner, D. T., Chang, A., Tobler, P. N., & Amodio, D. M. (2021). A computational reward learning account of social media engagement. *Nature Communications*, *12*(1), Article 1. https://doi.org/10.1038/s41467-020-19607-x

Matias, J. N. (2023). Humans and algorithms work together—So study them together. *Nature*, *617*(7960), 248–251. https://doi.org/10.1038/d41586-023-01521-z

Miller, K. J., Shenhav, A., & Ludvig, E. A. (2019). Habits without Values. *Psychological Review*, *126*(2), 292–311. https://doi.org/10.1037/rev0000120

Mirea, D.-M., Mildner, J., Kelley, S., Gillan, C., Nook, E., & Niv, Y. (2024). *Depression is associated with higher sensitivity to social media rewards*. OSF. https://doi.org/10.31234/osf.io/4ynbc

Niv, Y. (2007). Cost, Benefit, Tonic, Phasic. *Annals of the New York Academy of Sciences*, *1104*(1), 357–376. https://doi.org/10.1196/annals.1390.018

Niv, Y., Daw, N. D., Joel, D., & Dayan, P. (2007). Tonic dopamine: Opportunity costs and the control of response vigor. *Psychopharmacology*, *191*(3), 507–520. https://doi.org/10.1007/s00213-006-0502-4

Niv, Y., Daw, N., & Dayan, P. (2005). *How fast to work: Response vigor, motivation and tonic dopamine*.

Nussenbaum, K., & Hartley, C. A. (2019). Reinforcement learning across development: What insights can we draw from a decade of research? *Developmental Cognitive Neuroscience*, *40*, 100733. https://doi.org/10.1016/j.dcn.2019.100733

Orben, A. (2020). The Sisyphean Cycle of Technology Panics. *Perspectives on Psychological Science*, *15*(5), 1143–1157. https://doi.org/10.1177/1745691620919372

Orben, A., & Matias, J. N. (2025). Fixing the science of digital technology harms. *Science*, *388*(6743), 152–155. https://doi.org/10.1126/science.adt6807

Orben, A., Przybylski, A. K., Blakemore, S.-J., & Kievit, R. A. (2022). Windows of developmental sensitivity to social media. *Nature Communications*, *13*(1), 1649. https://doi.org/10.1038/s41467-022-29296-3

Palminteri, S., & Lebreton, M. (2022). The computational roots of positivity and confirmation biases in reinforcement learning. *Trends in Cognitive Sciences*, *26*(7), 607–621. https://doi.org/10.1016/j.tics.2022.04.005

Palminteri, S., Wyart, V., & Koechlin, E. (2017). The Importance of Falsification in Computational Cognitive Modeling. *Trends in Cognitive Sciences*, *21*(6), 425–433. https://doi.org/10.1016/j.tics.2017.03.011

Schultz, W., Dayan, P., & Montague, P. R. (1997). *A Neural Substrate of Prediction and Reward*. 7.

Thorndike, E. L. (1911). *Animal intelligence: Experimental studies* (pp. viii, 297). Macmillan Press. https://doi.org/10.5962/bhl.title.55072

Turner, G., Ferguson, A. M., Katiyar, T., Palminteri, S., & Orben, A. (2024). Old Strategies, New Environments: Reinforcement Learning on Social Media. *Biological Psychiatry*, *0*(0). https://doi.org/10.1016/j.biopsych.2024.12.012

Vrizzi, S., Najar, A., Lemogne, C., Palminteri, S., & Lebreton, M. (2025). Behavioral, computational and self-reported measures of reward and punishment sensitivity as predictors of mental health characteristics. *Nature Mental Health*, *3*(6), 654–666. https://doi.org/10.1038/s44220-025-00427-1

Wagenmakers, E.-J., & Farrell, S. (2004). AIC model selection using Akaike weights. *Psychonomic Bulletin & Review*, *11*(1), 192–196. https://doi.org/10.3758/bf03206482

Wilson, R. C., & Collins, A. G. (2019). Ten simple rules for the computational modeling of behavioral data. *eLife*, *8*, e49547. https://doi.org/10.7554/eLife.49547

Wood, W., Mazar, A., & Neal, D. T. (2022). Habits and Goals in Human Behavior: Separate but Interacting Systems. *Perspectives on Psychological Science*, *17*(2), 590–605. https://doi.org/10.1177/1745691621994226

A computational model of reward learning and habits on social media

Georgia Turner, Lukas J. Gunschera, Shashanka Subrahmanya, Aadesh Salecha, Johannes C. Eichstaedt, Stefano Palminteri* and Amy Orben*
* = joint senior authorship

Nature Communications: Reviewer Response

Reviewer #1 (Remarks to the Author):

I thank the authors for their thorough revision of the manuscript and for addressing all issues that I've raised previously.

We thank R1 for their thoughtful review which previously greatly improved the paper and for their appreciation of our previous response.

Reviewer #2 (Remarks to the Author):

The authors have done a great job overall with the review, and generally, all my original questions have been addressed. Congrats on a great paper!

Regarding the model formulation point, I have an additional concern:

The authors now fit the model described in Lindström et al 2021 to their data, and found their RL+Habit model to be better. The "Lindström model" included a parameter to set the initial policy, while the RL+Habit models did not (where the initial policy depends on the initial reward estimate, which the authors defined in a variety of different ways). This seems like an unfair comparison - it would therefore be instructive to fit the "Lindström model" using the same/similar approaches to initialize the policy as the RL+Habit models, thereby reducing the number of free parameters.

We thank R2 for pointing out a way in which we could test the robustness of the comparison to the Lindström model to variations of the model formulation. We followed their suggestion and fitted a new version of the Lindström model with one fewer parameter. However we found that this new version of the Lindström model provided a worse rather than better fit to the data than the original Lindström model. We have added this additional analysis to the section where we report the Lindström analysis in the Supplementary Material:

> *"We demonstrated that this finding that our RL-habit models fitted the data better than the Lindström RL model was robust to different modelling decisions, by creating a new variant of the Lindström RL model (henceforth: 'new Lindström RL model') which estimated initial policy from the data, by setting the initial policy as the mean of all posting latencies in each participant's dataset. Given that the original Lindström RL model fitted policy as a free parameter, this new variant had one fewer parameter than the original Lindström RL model, which might have been expected to give it more of an advantage in model fitting because the model fitting procedure, using Akaike Weights, penalised for each free parameter[24].*
>
> *However, the new Lindström RL model fitted the data less well than the original Lindström RL model. Specifically, when fitting only the original and new Lindström RL models to the data, results were as follows: original: AICw = 0.648; new: AICw = 0.352. By extension, this new Lindström model also performed worse than both the RLH1 and RLH2 models when compared to each of them separately in turn. Comparing the new Lindström RL model to our RLH1 resulted in AICw = 0.787 for the RLH1 model compared with AICw = 0.213 for the new Lindström RL model, while comparing the new Lindström RL model to our RLH2 resulted in AICw = 0.761 for the RLH2 model compared with AICw = 0.239 for the new Lindström RL model. In summary, our models still fitted the data better than the Lindström RL model, even after removing a free parameter from the Lindström RL model."* (pages 30-31, lines 664-685)

Reviewer #3 (Remarks to the Author):

Thank you for the thorough revision. You have addressed all my points adequately. I don't have additional ones. It has become (and already was) a great paper. You may all be very proud of this work that hopefully sets the stage to increasingly use RL models to investigate real-life learning behavior.

We thank R3 for their positive comments and for their very helpful suggestions on the original manuscript.
